# Asymmetric Synthesis of Stereogenic Phosphorus P(V) Centers Using Chiral Nucleophilic Catalysis

**DOI:** 10.3390/molecules26123661

**Published:** 2021-06-15

**Authors:** Ahmed Numan, Matthew Brichacek

**Affiliations:** Department of Chemistry, University of Maine, Orono, ME 04469, USA; Ahmed.Numan@maine.edu

**Keywords:** asymmetric synthesis, nucleophilic catalyst, phosphorylation, stereogenic phosphorus, dynamic kinetic resolution

## Abstract

Organophosphates have been widely used in agrochemistry, as reagents for organic synthesis, and in biochemistry. Phosphate mimics possessing four unique substituents, and thereby a chirality center, are useful in transition metal catalysis and as nucleotide therapeutics. The catalytic, stereocontrolled synthesis of phosphorus-stereogenic centers is challenging and traditionally depends on a resolution or use of stochiometric auxiliaries. Herein, enantioenriched phosphorus centers have been synthesized using chiral nucleophilic catalysis. Racemic *H*-phosphinate species were coupled with nucleophilic alcohols under halogenating conditions. Chiral phosphonate products were synthesized in acceptable yields (33–95%) and modest enantioselectivity (up to 62% *ee*) was observed after identification of an appropriate chiral catalyst and optimization of the solvent, base, and temperature. Nucleophilic catalysis has a tremendous potential to produce enantioenriched phosphate mimics that could be used as prodrugs or chemical biology probes.

## 1. Introduction

**Figure 1:**
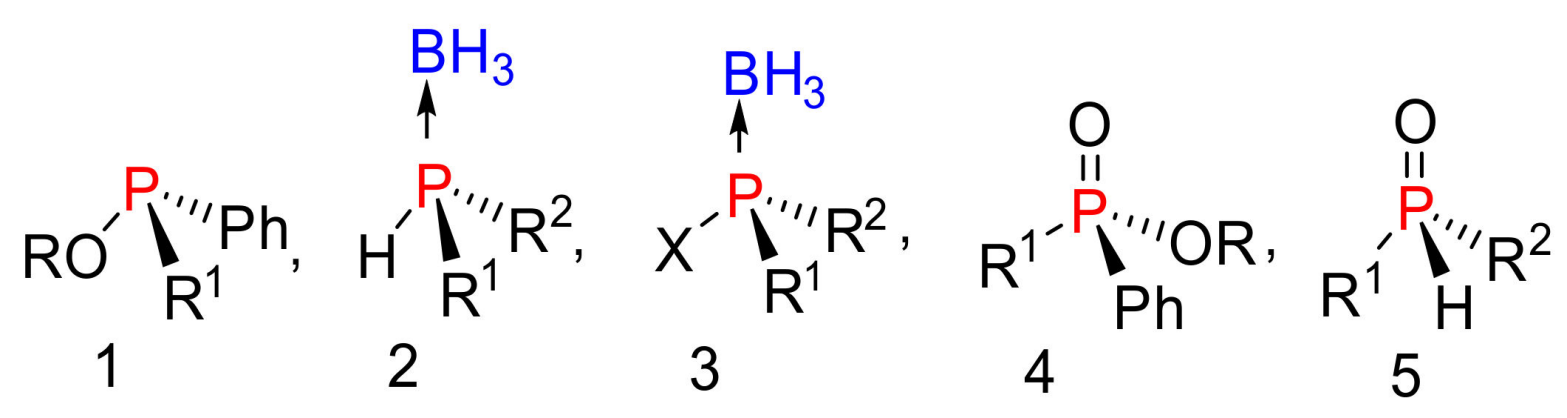
Structure of asymmetric phosphate mimics.

Organophosphorus compounds are common motifs in nucleotides, [1,2,3] pesticides, [4,5] herbicides, [6,7] and flame retardants [8]. Commonly, phosphate mimics with tri, tertra, or pentacoordinate geometries, with unique substitutions, create a chirality center on the phosphorus atom as shown in Figure 1. These compounds normally have stable configurations depending on the nature of the substitution around the phosphorus center [9]. Chiral phosphate mimics have been extensively utilized in transition metal catalysts, [10] as chiral reagents, [11,12,13] and in coordination chemistry [14,15]. Furthermore, compounds with stereogenic phosphonate centers are of great interest in biological applications [16]. For example, using phosphorus-stereogenic analogues in antisense therapy improves potency, stability against enzymatic degradation, and bioavailability [17]. However, current nucleotide therapeutics are stereoisomeric mixtures. Previous studies have shown that nucleotide stereoisomers at phosphorus possess distinct therapeutic activities and physical properties [18,19,20,21].

**Scheme 1:**
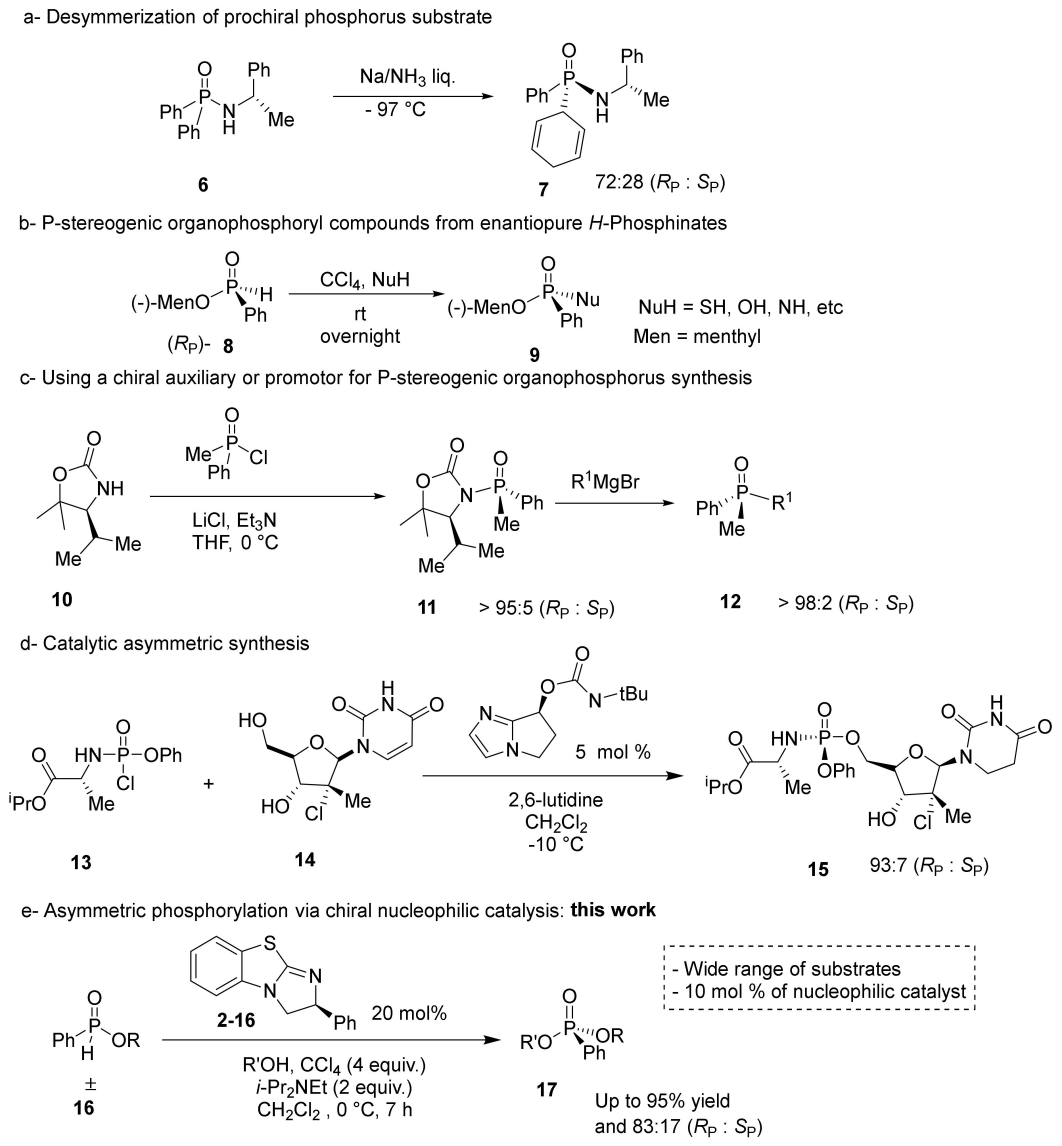
Synthetic approaches towards P-stereogenic organophosphorus compounds. (**a**) Desymmerization of prochiral phosphorus substrate, (**b**) P-stereogenic organophosphoryl compounds from enantiopure *H*-phosphinates, (**c**) using a chiral auxiliary or promotor for P-stereogenic organophosphorus synthesis, (**d**) catalytic asymmetric synthesis, (**e**) asymmetric phosphorylation via chiral nucleophilic catalysis.

Despite the importance of enantiopure P-stereogenic compounds, the chemical synthesis remains unsolved. Various strategies have been pursued using P-O, P-N, or P-C bond formations to create a stereogenic center [9]. Traditionally, P-stereogenic centers have been synthesized using multistep approaches involving the transfer of achiral phosphorus groups to chiral-pool amines and alcohol, as seen in Scheme 1a. However, the desymmetrization of prochiral substrates is limited by substrate scope and modest stereoselectivity [22].

Another approach to chiral phosphorus centers involves the transformation of nonracemic *H*-phosphinates (**8**) to corresponding P-stereogenic compounds (**9**) through a dehydrogenative coupling known as the Atherton–Todd reaction (Scheme 1b). Although *H*-phosphonates cleanly transfer the stereochemistry to the product, construction of the enantiopure substrate often requires multiple synthetic steps [23]. Alternatively, a racemic phosphorus center can be coupled with a chiral auxiliary or promotor, as shown Scheme 1c. However, a stoichiometric amount of chiral auxiliary is always required to achieve high stereoselectivity in the products. Moreover, a chiral auxiliary is covalently bonded to the product, necessitating subsequent chemical reactions and purifications to remove it from the desired product [24].

In 2010, Jordan et al. synthesized a phosphate moiety through a highly selective kinetic resolution using a chiral, peptide-based phosphorylation catalyst [25]. The chiral peptide was able to efficiently mimic enzyme catalysts that phosphorylate nucleotides using nucleophilic activation, leaving group activation, and oxyanion-hole transition state stabilization [26,27]. However, this approach is limited in scope because each peptide is optimized for a single substrate. Based on this work, DiRoco et al. designed a general chiral nucleophilic catalyst for the synthesis of enantiopure prodrugs via a dynamic kinetic asymmetric transformation (DKAT), as seen in in Scheme 1d [28]. The prodrug moiety contains an amino acid that makes hydrogen bonds to the nucleophilic catalyst to stabilize the transition state. Therefore, DiRoco’s catalyst is limited to phosphates with amino acid substituents [28]. Other approaches to produce P-stereogenic compounds, which are not shown in Scheme 1, include chiral-stationary phase separation and resolution using physical properties [9,29,30,31,32].

To overcome the synthetic limitations found in the synthesis of P-stereogenic phosphate mimics, a DKAT utilizing a chiral nucleophilic catalyst was pursued (Scheme 1e). A racemic *H*-phosphinate moiety (**16**) can be coupled to nucleophilic alcohols under halogenation conditions to produce chiral phosphonate products, as shown in Scheme 1e. This general approach would utilize a catalytic amount of a chiral substance, and a wide range of racemic phosphorus substrates could be used, including nucleotides or prodrug structures.

## 2. Results and Discussion

*H*-phosphinates and *H*-phosphonates are useful precursors to a wide range of P-stereogenic compounds [33,34,35]. Therefore, the racemic *H*-phosphinate moiety (**18**) was an ideal substrate to investigate the proposed DKAT using chiral nucleophilic catalysis. To obtain substrate **18**, esterification of phenyl phosphinic acid in neat isopropanol at an elevated temperature (138 °C) was performed and provided the product in 70% yield. Coupling of *H*-phosphinate **18** with phenol (**19**) under halogenation conditions (CCl_4_, 4 equiv.) provides the phosphonate product (**20**) in 76% yield. Stoichiometric amounts of *N,N*-diisopropylethylamine are included to consume the HCl byproduct of the halogenation process.

**Scheme 2:**
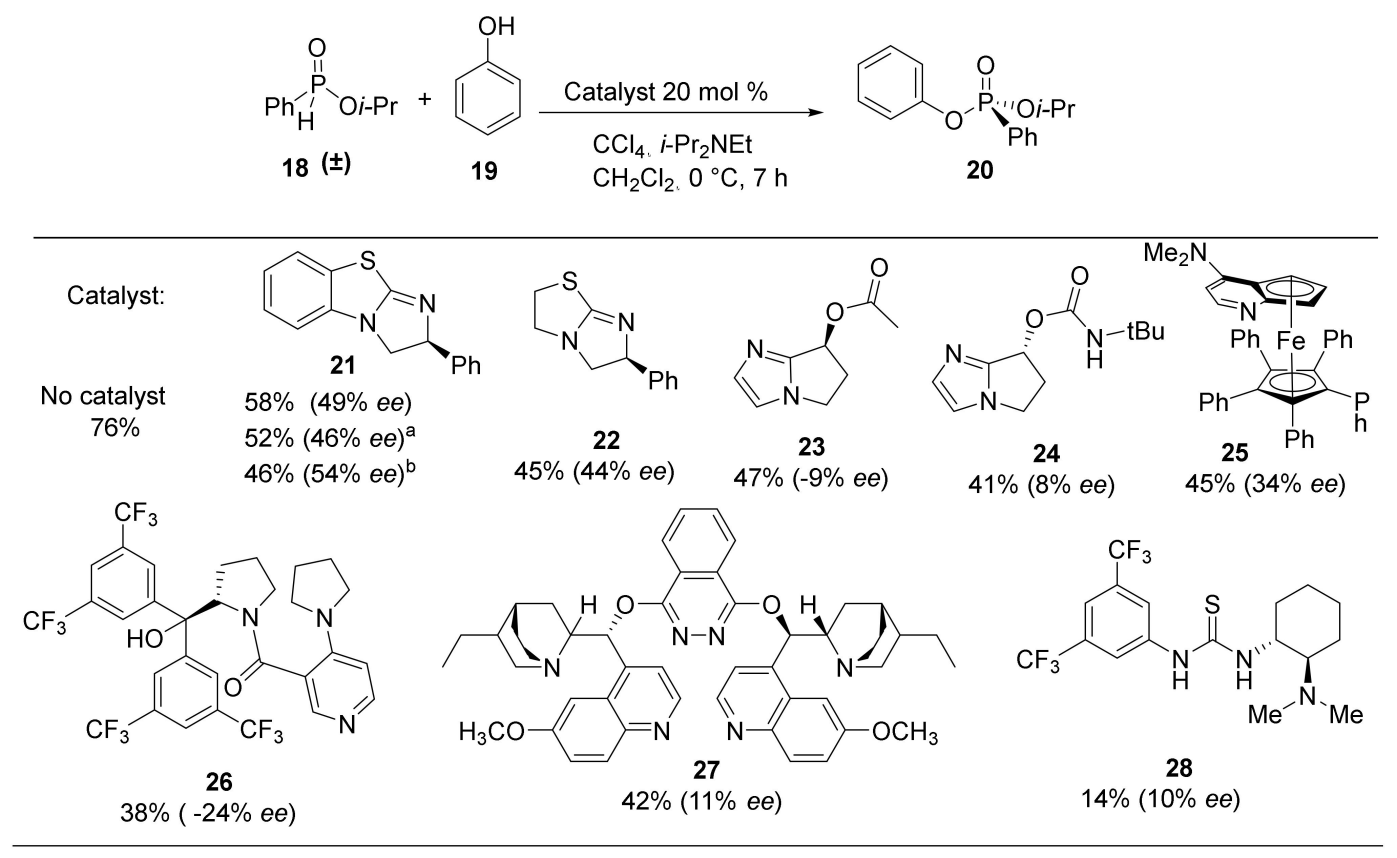
Nucleophilic catalysts investigated. Yield and enantiomeric excess of isolated products are given. Conditions: **21** (1 equiv.), CCl_4_ (4 equiv.), PhOH (0.5 equiv.) *i*-Pr_2_NEt (2 equiv.), Cat. 20 mol%, CH_2_Cl_2_ (0.1 M solution), 7 h; enantiomeric excess (*ee*) was determined by HPLC analysis; ^a^ catalyst 10 mol%. ^b^ catalyst 50 mol%.

Using optimized conditions for the racemic formation of **20**, a wide a variety of chiral nucleophilic catalysts were screened to quantify the enantiomeric enrichment of the phosphorus center (Scheme 2). Using the catalyst developed by DiRoco et al. (**23** and **24**), minimal stereoselectivity (−9% *ee* and 8% *ee*, respectively) was observed [28]. The suboptimal enantioselectivities are likely due to the remote chiral site being unable to effectively induce asymmetry in substrates that do not possess a rigid H-bonding functionality. Planar-chiral catalyst **25**, known as Fu’s catalyst, is a ferrocene analogue of 4-Diethylaminopyridine (DMAP) with high nucleophilicity and low steric hindrance at the nucleophilic nitrogen atom [36]. The product **20** was obtained with an improved selectivity of 34% *ee* and a moderate yield of 45%. Alternative heterocyclic nucleophilic catalysts **26** and **27** provide inferior enantioselectivities (-24% and 11% *ee*) and chemical yields (38% and 42%). Chiral thiourea **28**, which does not operate as a nucleophilic catalyst, does not induce significant levels of stereoselectivity (10% *ee*) while resulting in a dramatically diminished yield of 14%. Despite limited success with common chiral catalysts, the results demonstrate the enantiomeric enrichment was significantly impacted by catalyst structure.

Benzotetramisole (BTM, 21) was originally developed by Briman et al. for the kinetic resolution of alcohols and is now commercially available [37,38,39]. BTM was tested in the standard *H*-phosphinate coupling of **18** and **19** under halogenation conditions and was found to produce the highest levels of enantioselectivity. Specifically, 20 mol% of (*S*)-BTM produced phosphonate **20** in 58% yield and 49% *ee* (Scheme 2). Increasing the catalyst loading from 10 mol% to 20 mol% and to 50 mol% resulted in small increases in enantioselectivity of 46%, 49%, and 54% *ee*, respectively. Structurally similar Tetramisole (22) is a capable catalyst providing **20** in 45% yield and 44% *ee.* Mechanistically, we envisioned the nucleophilic BTM catalyst becoming covalently bonded to the phosphoryl center while facilitating racemization to produce the observed enantioenrichment. Consequently, BTM was identified as the optimal catalyst for the DKAT of racemic *H*-phosphinates into chiral phosphonates.

In order to optimize the yield and enantioselectivity of **20**, the effect of the base was investigated, and results are shown in Table 1. As established in the catalyst screen, *N,N*-diisopropylethylamine (DIPEA) has the highest enantioselectivity (49% *ee*) and a respectable yield of 58% (Table 1, Entry 7). Among the investigated bases, potassium *tert*-butoxide (KOtBu) provided the highest chemical yield of 63%, but with near-complete loss of enantioselectivity (7% *ee*). Non-nucleophilic amine bases, including DBU, ABCO, and DABCO, provided **20** with diminished enantioselectivities of 20%, 30%, and 33% *ee*, respectively. The starting material was recovered, and no product was observed when using hindered bases such as proton sponge (1,8-bis(dimethylamino)naphthalene) or di-*tert*-butyl pyridine (Table 1, Entries 5 and 6). All other reactions showed complete consumption of the *H*-phosphinate starting material and no ongoing product formation as judged by ^31^P NMR. 

After identifying *N,N*-diisopropylethylamine as the optimal base, the effect of solvent and temperature were evaluated, and results are shown in Table 2. The nonpolar solvent toluene results in nearly identical enantioselectivity when compared to the standard solvent methylene chloride, except with a dramatic decrease in yield to 17%. In contrast, acetonitrile reduced the enantioselectivity to 26% *ee* but formed the product in similar efficiency (47%) to methylene chloride. Interestingly, the production of **20** is nearly eliminated when using tetrahydrofuran (THF) with respectable recovery of the starting material. 

After confirming dichloromethane as a suitable solvent for the DKAT, temperature effects on enantioselectivity were explored. Phosphorylation of **19** at −40 °C, 0 °C, and 25 °C resulted in enantioselectivities of 44%, 49%, and 45% *ee*, respectively. Therefore, the optimal temperature was determined to be 0 °C, but a smaller than expected influence on enantioselectivity was observed. In addition, the product was obtained in the highest yield at 0 °C (58%), while increasing or decreasing the temperature resulted in a reduction in yield.

To assess the *H*-phosphinate substrate scope, the alcohol and the aryl group were varied and coupled with phenol under the optimized reaction conditions (Table 3). Of note, the experiments in Table 3 were performed with an altered stoichiometry (0.5 equiv. of alcohol) to improve the enantioselectivity of the transformation at the expense of chemical yield. The products (**30**–**33**) of ethyl-substituted *H*-phosphinates are obtained in comparable enantioselectivities but with typically higher yield than **20** due to the decreased steric environment around the phosphorus center (Table 3, Entries 3–6). As an aside, the HPLC elution order of methyl-substituted phosphonate (**29)** was reversed compared to other synthetic phosphonate analogues due to indeterminate interactions with the stationary phase (Figure S58 in Supplementary Materials). Modulation of the aromatic group on the *H*-phosphinate was also investigated. When dramatically altering the steric environment around the phosphorus by replacing the phenyl group with an o-tolyl (Entries 3 and 4), the yield is unsurprisingly decreased from 95% to 67%. However, the increased steric hindrance did not improve the enantioselectivity. Additionally, electronic effects were studied by substituting the phenyl group with a 4-methoxy- and a 4-fluoro-phenyl group, respectively. A decline in enantioselectivity to 22% *ee* was obtained with both the electron-withdrawing and -donating substituents as shown in Table 3, Entries 5 and 6. The substrate scope for the *H*-phosphinate coupling was found to be very sensitive to the steric and electronic environment around the phosphorus center with improved yields and enantioselectivities observed when compared to the model substrate (**18**), but with varied results even under the optimized conditions. 

**Figure 2:**
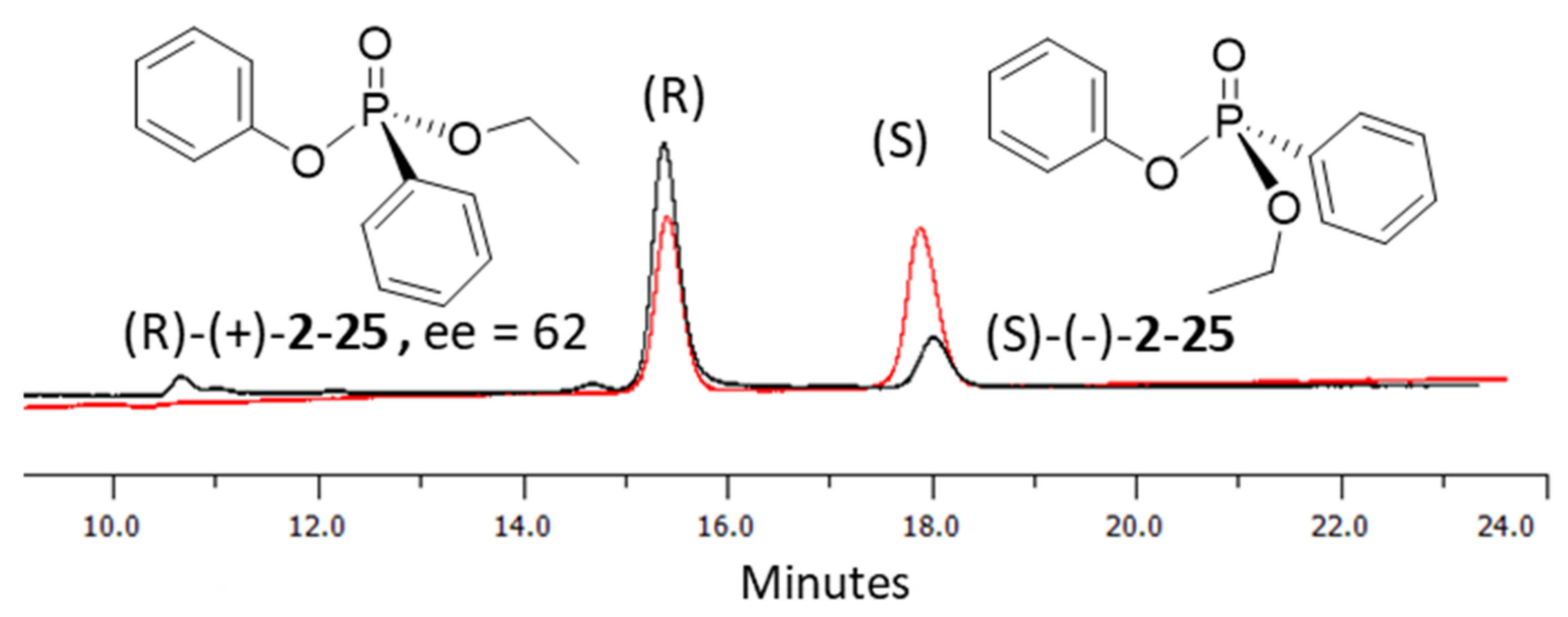
HPLC chromatograms of racemic (red) and chiral phosphonate analogues (black) of **30** using (*S*)-**21**. *Conditions.* Phenomenex Lux amylose-2 chiral column (250 × 4.6 mm), eluent = hexane: ethanol (15:95 to 20:80) with 0.1% TFA, detection = 254 nm, flow rate = 0.5 mL/min, temperature = 25 °C.

In the process of exploring the substrate scope in Table 3, known compounds **29** and **30** were prepared to correlate the observed enantioselectivity to an absolute configuration. High performance liquid chromatography (HPLC) on a chiral stationary phase was used to measure all enantioselectivities reported. As seen in Figure 2, using a normal phase eluent of hexane and ethanol with 0.1% trifluoroacetic acid (TFA) baseline resolves a racemic sample of **30** (red trace) formed when no chiral catalyst is used. The enantioenriched product from Table 3, Entry 3, shows the correlating imbalance in peak areas (62% *ee*) when using (−)- or (*S*)-benzotetramisole (Figure 2, black trace). Correspondingly, **30** has a measured ${\left[\alpha \right]}_{D}^{20}$ of +21.7° (c 0.43, CHCl_3_). Using the published data and sign for (*R*)-**30** (${\left[\alpha \right]}_{D}^{20}$ + 34.4° (c 2.16, CHCl_3_), we observe that (*S*)-BTM produces the (*R*) enantiomer of **30** as the major product [44]. In addition, (*R*)-**29** was produced using (*S*)-BTM because the specific rotation agrees with reported values [44]. Finally, when the enantiomer of the catalyst ((*R*)-**21**) was used to synthesize **20**, a near-equal yield, but with opposite enantioselectivity (−54% *ee*), was observed, as expected.

Using catalyst (*S*)-21, a number of alcohols were tested as nucleophiles in the phosphorylation reaction to produce the corresponding phosphonates in Table 4. Our standard phosphorylation reaction utilized an aromatic alcohol (19) as a nucleophile, so it was encouraging that BTM was an effective catalyst for alcohols such as allyl, benzyl, methyl, or phenethyl alcohol. Under the optimized conditions, allyl alcohol, which differs greatly in structure from phenol, reacted with isopropyl-*H*-phosphinate in similar efficiency (49% yield and 43% *ee*). Small decreases in enantioselectivity (28% and 35% *ee*) were observed when using phenethyl alcohol or benzyl alcohol as nucleophiles, but comparable yields were obtained. Under the optimized reaction conditions, (*S*)-BTM (21) provided the same stereochemical outcome as the (*R*)-phosphonate analogues in Table 4. However, due to Cahn–Ingold–Prelog rules, the priority of the substituents on phosphorus changed and resulted in the formation of (*S*)-34 through (*S*)-37. Finally, even when using sterically unencumbered methanol, the product (37) yield and enantioselectivity were not decreased (56% yield and 51% *ee*). Similar to compound **29**, the HPLC elution order of the methyl phosphonate analogue (37) was reversed from the normal pattern (Supplementary Materials, Figure S70). Moreover, (*S*)-37 had a positive specific optical rotation which is consistent with analogous phosphonates [45].

**Scheme 3:**
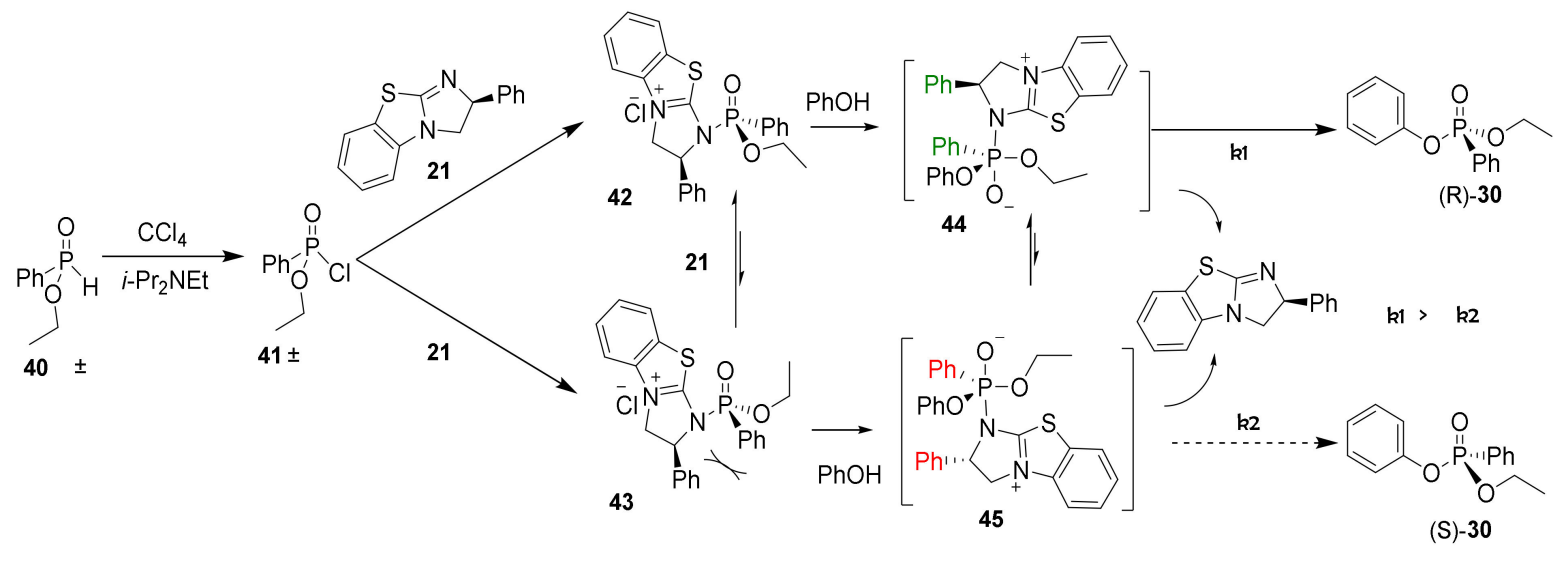
Proposed asymmetric phosphorylation mechanism based on the dynamic kinetic asymmetric transformation of *H*-phosphinate **40** to phosphonate (*R*)-**30** and (*S*)-**30** using chiral nucleophilic catalyst **21**.

Based on the Atherton–Todd reaction, a mechanism is proposed that explains the formation of enantioenriched phosphonates (**30**) using the chiral nucleophilic catalyst **21** [46]. First, the chlorination of the *H*-phosphinate moiety (**40**) using tetrachloromethane as the stoichiometric oxidant results in the formation of racemic phosphonochloridate **41** (Scheme 3). While monitoring the reaction using ^31^P NMR, a species (29.3 ppm) consistent with a phosphonochloridate (**41**) was observed with concomitant consumption of the *H*-phosphinate [47]. Next, catalyst (*S*)-(**21**) attacks the phosphonochloridate and results in diastereomers **42** and **43** after substitution of the chlorine. Unfortunately, ^31^P NMR did not allow unambiguous identification of an intermediate where the BTM catalyst was bonded to the phosphoryl center. Substitution reactions at phosphorus centers typically involve either 1) a dissociative S_N_1-type mechanism through the formation of stable metaphosphate (PO_3_^−^) or 2) an associative, two-step addition-elimination mechanism through the formation of a phosphorane intermediate [48]. In this case, the elementary steps of the substitution process are inconsequential to the product outcome.

Nucleophilic addition by phenol forms pentavalent intermediates **44** and **45**. Additional pentavalent intermediate isomers are likely formed in the reaction but not shown in Scheme 3. Elimination of the BTM catalyst from the apical position of intermediate **44** provides the observed product **30** in the observed (*R*) configuration [48]. Elimination from pentavalent **44** is preferable to elimination from **45** because steric interactions between the phenyl groups on the phosphonate and the catalyst (*S*)-(**21**) are avoided. Alternatively, the stereoselectivity could be a result of π-π or π-cation interactions between the catalyst and phosphorus substrate, analogous to previously published acylation studies with **21** [37]. It is known from the literature that trigonal bipyramidal phosphorus centers, such as **44** and **45**, readily undergo pseudorotation and provide an equilibration mechanism consistent with the dynamic kinetic transformation observed [48]. Alternatively, diastereomers **42** and **43** are potentially equilibrating during the reaction process, but as mentioned above, this could not be spectroscopically observed. Thus, enantiomeric enrichment is a result of the faster elimination (k1 > k2) of **44** and constitutes the stereodetermining step.

Collectively, the reaction stereoselectivity was dependent on the solvent, catalyst structure and loading, and the nature of the base. The proposed mechanism explains the enantioselectivity dependence on the reaction conditions perturbing the relative rates of elimination (k1, k2). The similarity of these rates of elimination results in the modest stereoselectivity observed in this study. In contrast, the enantioselectivity was largely unaffected by the reaction temperature, indicating that kinetic control was observed as opposed to thermodynamic control. Finally, the proposed reaction mechanism in Scheme 3 shows the selective formation of the (*R*)-phosphonate using (*S*)-(−)-benzotetramisole catalyst (**21**) consistent with optical rotations and absolute configurations [44]. Modifying the BTM catalyst by increasing the steric hindrance of the phenyl group or electronically perturbing the fused benzo system may provide an opportunity to improve the stereoinduction.

## 3. Materials and Methods

### General Information

All reactions were carried out under a nitrogen atmosphere in oven-dried glassware. The products were purified by flash column chromatography using silica gel 60 (230–400 mesh Silicycle, Quebec City, Quebec, Canada). Solvent, reagents, and chemicals were received from a commercial vendor and further dried under activated 4 Å molecular sieves. Methylene chloride and THF were dried using the PURE SOLV MICRO purification system. Thin-layer chromatography (TLC) analysis was performed on Merck 0.25 mm silica gel 60 F254 plates. ^1^H NMR was recorded using an Oxford Varian Unity Instrument (Palo Alto, Santa Clara, CA, USA) at 400 MHz and referenced using the residual solvent peak of CDCl_3_ at 7.26 ppm. ^13^P NMR spectra were acquired at 162 MHz and externally referenced to 85% H_3_PO_4_ at (0 ppm). The ^13^C NMR were acquired at 101 MHz and referenced to 77.1 ppm for the CDCl_3_ solvent. The NMR data was processed with MestReNova11 NMR software. HPLC was performed using a Gilson GX-271 instrument (Middleton, Wisconsin, United States of America) at 254 nm with Trilution LC V3 software. Analytical HPLC was carried out using a 5 μm 250 × 4.6 mm Phenomenex Lux chiral column (Amylose-2 or Cellulose-2). In analytical HPLC, the normal phase separation method used was: eluent = hexane: ethanol (15:95 to 20:80) with 0.1% TFA, detection (254 nm), flow rate (0.5 mL/min), temperature (25 °C), and run time (20 min). Mass spectra were measured by the University of Illinois Mass Spectrometry Laboratory using the electrospray ionization technique (ESI). The optical rotations were measured for purified samples using the digital polarimeter JASCO (model DIP-370). Specific rotation (${\left[\alpha \right]}_{D}^{20}$) was reported for samples in solution at 20 °C in which the symbol D represents the sodium D line (589 nm) and concentration in units of g/100 mL.

*Isopropyl (phenyl)-H-phosphinate (***18***)*

A mixture of phosphinic acid (1.00 g, 7.04 mmol, 1.0 equiv.) and isopropanol (10 mL, 0.131 mol, 18.6 equiv.) was stirred in a well-sealed, 20 mL vial for 15 h at 138 °C. Then, the solvent was evaporated under vacuum. The crude product was purified using silica gel column chromatography with 5% MeOH: 95% CH_2_Cl_2_ as an eluent to provide a colorless oil in 70% yield (0.90 g). The NMR data matched previously published results [49]. ^1^H NMR (400 MHz, CDCl_3_) δ 7.81–7.70 (m, 2H), 7.59 (d, *J* = 558.7 Hz, 1H, PH), 7.60–7.52 (m, 1H), 7.52–7.44 (m, 2H), 4.73–4.67 (m, 1H, OCH), 1.41 (d, *J* = 6.1 Hz, 3H, CH_3_), 1.33 (d, *J* = 6.1 Hz, 3H, CH_3_). ^13^C NMR (101 MHz, CDCl_3_) δ 133.1 (d, *J* = 2.9 Hz), 131.0 (d, *J* = 11.9 Hz), 130.6 (d, *J* = 133.0 Hz), 128.8 (d, *J* = 13.9 Hz), 71.5 (d, *J* = 5.5 Hz), 24.4 (d, *J* = 4.5 Hz), 24.0 (d, *J* = 4.2 Hz). ^31^P NMR (162 MHz, CDCl_3_) δ 22.5 (d, *J* = 21.5 Hz).

*Ethyl (phenyl)-H-phosphinate (***40***)*

A mixture of phosphinic acid (1.00 g, 7.04 mmol, 1.0 equiv.) in ethanol (10 mL, 0.171 mol, 24.4 equiv.) was stirred in a well-sealed, 20 mL vial for 15 h at 133 °C. Then, the solvent was evaporated under vacuum. The product was purified by flash chromatography on silica gel using 5% MeOH: 95% CH_2_Cl_2_ as an eluent to produce a colorless oil in 55% yield (0.65 g). The NMR data matched previously published results [33]. ^1^H NMR (400 MHz, CDCl_3_) δ 7.74 (m, 2H), 7.59–7.51 (m, 1H), 7.54 (d, *J* = 562.5 Hz, 1H, PH), 7.50–7.42 (m, 2H), 4.17–4.05 (m, 2H), 1.33 (t, *J* = 7.1 Hz, 3H). ^13^C NMR (101 MHz, CDCl_3_) δ 133.2 (d, *J* = 2.8 Hz), 131.0 (d, *J* = 11.9 Hz), 129.9 (d, *J* = 132.3 Hz), 128.9 (d, *J* = 13.9 Hz), 62.3 (d, *J* = 6.5 Hz), 16.5 (d, *J* = 6.5 Hz). ^31^P NMR (162 MHz, CDCl_3_) δ 24.96 (d, *J* = 19.3 Hz).

*Methyl (phenyl)-H-phosphinate (***46***)*

A mixture of phosphinic acid (1.00 g, 7.04 mmol, 1.0 equiv.) in methanol (15 mL, 0.370 mol, 52.9 equiv.) was stirred in a well-sealed, 20 mL vial for 15 h at 125 °C. Then, the solvent was evaporated under vacuum. The crude product was purified by flash chromatography on silica gel using 5% MeOH: 95% CH_2_Cl_2_ as an eluent to produce a colorless oil in 62% yield (0.68 g). The NMR data matched previously published results [50]. ^1^H NMR (400 MHz, CDCl_3_) δ 7.85–7.73 (m, 2H), 7.65–7.56 (m, 1H), 7.56 (d, *J* = 565.8 Hz, 1H, PH), 7.55–7.47 (m, 2H), 3.79 (d, *J* = 12.0 Hz, 3H). ^13^C NMR (101 MHz, CDCl_3_) δ 133.4 (d, *J* = 2.8 Hz), 131.2 (d, *J* = 11.9 Hz), 130.3 (d, *J* = 47.2 Hz), 129.0 (d, *J* = 13.8 Hz), 52.3 (d, *J* = 6.6 Hz). ^31^P NMR (162 MHz, CDCl_3_) δ 27.46 (d, *J* = 21.6 Hz).

*Ethyl (2-methylphenyl)-H-phosphinate (***47***)*

A solution of P(OEt)_3_ (0.970 g, 5.84 mmol, 1.0 equiv.) in diethylether (0.71 mL) was added dropwise under N_2_ to a solution of o-tolyl magnesium bromide (5.14 mL, 2 M, 10.28 mmol, 1.76 equiv.) in diethyl ether. After stirring the mixture at 34 °C for 5 h, a solution of 1 M HCl was added at room temperature until the pH of the solution was 2. After stirring the mixture for another hour, the mixture was extracted with ethyl acetate (2 mL × 4). The combined organic layers were dried over MgSO_4_. The solvent was removed under reduced pressure and purified by flash chromatography on silica gel using 5% MeOH: 95% CH_2_Cl_2_ to give an oily product in 63% yield (0.675 g). ^1^H NMR (400 MHz, CDCl_3_) δ 7.77 (m, 1H), 7.60 (d, *J* = 555.1 Hz, 1H, PH), 7.48–7.40 (m, 1H), 7.29 (m, 1H), 7.26–7.19 (m, 1H), 4.13 (m, *J* = 8.8, 7.1 Hz, 2H), 2.53 (s, 3H), 1.34 (t, *J* = 7.1 Hz, 6H). ^13^C NMR (101 MHz, CDCl_3_) δ 141.2 (d, *J* = 10.8 Hz), 133.1 (d, *J* = 3.2 Hz), 132.1 (d, *J* = 7.2 Hz), 131.3 (d, *J* = 5.0 Hz), 128.2 (d, *J* = 131.1 Hz), 126.0 (d, *J* = 14.1 Hz), 62.2 (d, *J* = 6.0 Hz), 20.1 (d, *J* = 6.9 Hz), 16.5 (d, *J* = 3.1 Hz). ^31^P NMR (162 MHz, CDCl_3_) δ 25.93 (d, *J* = 14.8 Hz).

*Ethyl (4-methoxyphenyl)-H-phosphinate (***48***)*

A solution of P(OEt)_3_ (0.100 g, 0.602 mmol, 1.0 equiv.) in THF (1.2 mL) was added dropwise under N_2_ to a solution of 4-methoxyphenylmegnesium-bromide (1.8 mL, 0.5 M, 0.900 mmol, 1.5 equiv.) in THF. After stirring the mixture at 66 °C for 5 h, a solution of 1 M HCl was added at room temperature until the pH of the solution was 2. After stirring the mixture for another hour, the mixture was extracted with ethyl acetate (2 mL × 4). The combined organic layers were dried over MgSO_4_. The solvent was removed under reduced pressure and purified by flash chromatography on silica gel using 5% MeOH: 95% CH_2_Cl_2_ to give an oily product in 87% yield (105 mg). The NMR data matched previously published results [33]. ^1^H NMR (400 MHz, CDCl_3_) δ 7.76–7.64 (m, 2H), 7.54 (d, *J* = 561.7 Hz, 1H, HP), 7.00 (m, 2H), 4.16–4.08 (m, 2H), 3.85 (s, 3H), 1.36 (t, *J* = 7.1 Hz, 3H). ^13^C NMR (101 MHz, CDCl_3_) δ 163.5 (d, *J* = 3.2 Hz), 133.2 (d, *J* = 13.2 Hz), 121.3 (d, *J* = 138.8 Hz), 114.5 (d, *J* = 14.9 Hz), 61.9 (d, *J* = 6.4 Hz), 55.6 – 55.5 (m), 16.6 (d, *J* = 6.6 Hz). ^31^P NMR (162 MHz, CDCl_3_) δ 23.68 (d, *J* = 17.1 Hz).

*Ethyl (4-fluorophenyl)-H-phosphinate (***49***)*

A solution of P(OEt)_3_ (0.500 g, 3.01 mmol, 1.0 equiv.) in THF (3.76 mL) was added dropwise under N_2_ to solution of 4-fluoro magnesium bromide (4.5 mL, 0.8 M, 3.60 mmol, 1.2 equiv.) in THF. After stirring the mixture at 66 °C for 12 h, a solution of 1 M HCl was added at room temperature until the pH of the solution was 2. After stirring the mixture for another hour, the mixture was extracted with ethyl acetate (2 mL × 4). The combined organic layers were dried over MgSO_4_. The solvent was removed under reduced pressure and purified by flash chromatography on silica gel using 5% MeOH: 95% CH_2_Cl_2_ to give an oily product in 27% yield (153 mg). The NMR data matched previously published results [33]. ^1^H NMR (400 MHz, CDCl_3_) δ 7.81–7.73 (m, 2H,), 7.55 (d, *J* = 567.1 Hz, 1H, PH) 7.18 (m, 2H), 4.27–4.06 (m, 2H, OCH_2_), 1.36 (t, *J* = 7.1 Hz, 3H, CH_3_). ^13^C NMR (101 MHz, CDCl_3_) δ 167.2 (d, J_CP_ = 3.2 Hz), 164.7 (d, JCF = 3.4 Hz), 133.8 (dd, JCP = 22.2 Hz JCF = 4.1 Hz), 127.6 (dd, JCP = 7.3 Hz, JCF = 7.9 Hz), 116.4 (dd, JCP = 36.7 Hz, JCF = 6.5 Hz), 62.4 (d, JCP = 6.2 Hz), 16.5 (d, JCP = 6.6 Hz). ^31^P NMR (162 MHz, CDCl_3_) δ 23.48 (d, *J* = 18.0 Hz).

*General procedure for isopropyl (alkyl)-phenyl-phosphonate*

Isopropyl phenyl-*H*-phosphinate (**18**) (0.100 g, 0.543 mmol, 1.0 equiv.) and carbon tetrachloride (0.21 mL, 2.170 mmol, 4.0 equiv.) were added to a 7-mL reaction vial under an atmosphere of N_2_. Next, anhydrous CH_2_Cl_2_ (0.3 M solution) was added, and the solution was cooled to 0 °C. A solution of *N,N*-diisopropylethylamine (0.14 g, 1.080 mmol, 2 equiv.), benzotetramisole (**21**) (0.03 g 0.110 mmol, 0.2 equiv.), and an alcohol (0.270 mmol, 0.5 equiv.) that was previously dried over 4 Å molecular sieves, in anhydrous CH_2_Cl_2_ (0.3 mL), was prepared. The alcohol solution was added dropwise to the original phosphinate solution and stirred for 9 h at 0 °C. The mixture was quenched with 1M HCl (1.0 mL) and extracted three times with ethyl acetate and then dried using NaSO_4_. The product was purified by silica gel chromatography with ethyl acetate: hexane (30:70) as an eluent. The following products were synthesized.

*(R)-Isopropyl (phenyl)-phenyl-phosphonate (***20***)*

Yield 35 mg (47%). The NMR data matched for previously published results [16]. ^1^H NMR (400 MHz, CDCl_3_) δ 7.89–7.82 (m, 2H), 7.56–7.50 (m, 1H), 7.47–7.41 (m, 2H), 7.25 (t, *J* = 7.9 Hz, 1H), 7.16–7.12 (m, 2H), 7.12 – 7.06 (m, 3H), 4.91–4.81 (m, 1H), 1.35 (d, *J* = 6.2 Hz, 3H), 1.30 (d, *J* = 6.2 Hz, 3H). ^13^C NMR (101 MHz, CDCl_3_) δ 150.8 (d, *J* = 7.3 Hz), 132.7 (d, *J* = 7.9 Hz), 132.1 (d, *J* = 16.1 Hz), 129.7 (d, *J* = 7.7 Hz), 128.6 (d, *J* = 25.9 Hz), 128.6 (dd, *J* = 108.8, 82.8 Hz), 120.8 (d, *J* = 2.6 Hz), 72.3 (d, *J* = 5.9 Hz), 24.1 (d, *J* = 2.3 Hz), 24.0 (d, *J* = 5.6 Hz). ^31^P NMR (162 MHz, CDCl_3_) δ 14.92. ${\left[\alpha \right]}_{D}^{20}$ + 15.1° (c 0.26, CHCl_3_, 55% *ee*). The absolute configuration of compound **20** is designated the (*R*)-configuration based on the optical rotation of the previously published analogue **30**.

*(S)-Isopropyl (allyl)-phenyl-phosphonate (***34***)*

Yield 31.8 mg (49%). ^1^H NMR (400 MHz, CDCl_3_) δ 7.85–7.74 (m, 2H), 7.56–7.49 (m, 1H), 7.47–7.39 (m, 2H), 5.90 (m 2H), 5.30 (dq, *J* = 17.1, 1.6 Hz, 2H), 5.18 (dq, *J* = 10.4, 1.4 Hz, 1H), 4.73 (m, 1H), 4.58–4.41 (m, 1H), 1.30 (dd, *J* = 48.2, 6.2 Hz, 6H). ^13^C NMR (101 MHz, CDCl_3_) δ 133.1 (d, *J* = 6.5 Hz), 132.5 (d, *J* = 2.2 Hz), 131.9 (d, *J* = 10.0 Hz), 130.0 (s), 128.6 (d, *J* = 15.2 Hz), 71.3 (d, *J* = 6.6 Hz), 66.4 (d, *J* = 4.7 Hz), 24.2 (d, *J* = 3.6 Hz), 24.0 (d, *J* = 4.9 Hz). ^31^P NMR (162 MHz, CDCl_3_) δ 18.53. HRMS (ES): *m/z* [M + H]^+^: calcd for C_12_H_18_O_3_P 241.0994; found: 241.0983. ${\left[\alpha \right]}_{D}^{20}$ − 1.3° (c 0.23, CHCl_3_). The absolute configuration of compound **34** is designated to be the (*S*)-configuration based on the analogous reactivity of **30**.

*(S)-Phenethyl (isopropyl)-phenyl-phosphonate (***35***)*

Yield 55.9 mg (68%). ^1^H NMR (400 MHz, CDCl_3_) δ 7.76–7.68 (m, 2H), 7.54–7.48 (m, 1H), 7.44–7.36 (m, 2H), 7.30–7.14 (m, 5H), 4.73–4.60 (m, 1H), 4.30–4.08 (m, 2H), 2.97 (t, *J* = 7.1 Hz, 2H), 1.27 (dd, *J* = 41.1, 6.2 Hz, 6H). ^13^C NMR (101 MHz, CDCl_3_) δ 137.5 (s), 132.4 (d, *J* = 6.9 Hz), 131.9 (d, *J* = 8.0 Hz), 129.2 (d, *J* = 2.8 Hz), 129.0 (d, *J* = 188.6 Hz), 128.6 (d, *J* = 2.2 Hz), 128.5 (d, *J* = 9.6 Hz), 126.8 (d, *J* = 2.8 Hz), 71.2 (d, *J* = 14.7 Hz), 66.4 (d, *J* = 5.6 Hz), 37.1 (d, *J* = 6.7 Hz), 24.2 (d, *J* = 9.0 Hz), 24.0 (d, *J* = 9.9 Hz). ^31^P NMR (162 MHz, CDCl_3_) δ 18.15. HRMS (ES)+: *m/z* [M + H]^+^ calcd for C_17_H_22_O_3_P: 305.1307; found: 305.1302. ${\left[\alpha \right]}_{D}^{20}$ − 2.9° (c 0.68, CHCl_3_). The absolute configuration of compound **35** is designated to be the (*S*)-configuration based on the analogous reactivity of **30**.

*(S)-Benzyl (isopropyl)- phenyl-phosphonate (***36***)*

Yield 40.0 mg (51%). ^1^H NMR (400 MHz, CDCl_3_) δ 7.84–7.77 (m, 2H), 7.55–7.50 (m, 1H), 7.43 (m, 2H), 7.35–7.26 (m, 5H), 5.04 (m 2H), 4.73 (m, 1H), 1.29 (dd, *J* = 37.2, 6.2 Hz, 6H). ^13^C NMR (101 MHz, CDCl_3_) δ 136.5 (d, *J* = 7.2 Hz), 132.5 (d, *J* = 3.0 Hz), 131.9 (d, *J* = 9.9 Hz), 129.0 (d, *J* = 189.1 Hz), 128.6 (s), 128.5 (s), 128.4 (s), 127.9 (s), 71.4 (d, *J* = 4.0 Hz), 67.4 (d, *J* = 5.1 Hz), 24.2 (d, *J* = 4.1 Hz), 24.0 (d, *J* = 4.9 Hz). ^31^P NMR (162 MHz, CDCl_3_) δ 19.25. HRMS (EI) *m/z* [M + H]^+^ calcd for C_16_H_19_O_3_P: 290.10719; found: 290.10667. ${\left[\alpha \right]}_{D}^{20}$ − 6.3 (*c* 0.44, CHCl_3_). The absolute configuration of compound **36** is designated to be the (*S*)-configuration based on the analogous reactivity of **30**.

*(S)-Isopropyl (methyl)-phenyl-phosphonate (***37***)*

Yield 32.4 mg (56%). The NMR data matched previously published results [16]. ^1^H NMR (400 MHz, CDCl_3_) δ 7.80–7.73 (m, 2H), 7.54–7.48 (m, 1H), 7.45–7.38 (m, 2H), 4.70 (m *J* = 2H), 3.68 (d, *J* = 11.2 Hz, 3H), 1.35 (d, *J* = 6.2 Hz, 3H), 1.22 (d, *J* = 6.2 Hz, 3H). ^13^C NMR (101 MHz, CDCl_3_) δ 132.5 (d, *J* = 2.9 Hz), 131.9 (d, *J* = 9.8 Hz), 128.6 (d, *J* = 15.0 Hz), 128.6 (d, *J* = 188.7 Hz), 71.3 (d, *J* = 5.4 Hz), 52.5 (d, *J* = 2.8 Hz), 24.2 (d, *J* = 3.9 Hz), 24.0 (d, *J* = 4.9 Hz). ^31^P NMR (162 MHz, CDCl_3_) δ 19.12. ${\left[\alpha \right]}_{D}^{20}$ + 0.19° (c 0.54, CHCl_3_). The absolute configuration of compound **37** is designated to be the (*S*)-configuration based on the analogous structure (*S*)-Ethyl (Methyl)-phenyl-phosphonate [45].

*(R)-Methyl (phenyl)-phenyl-phosphonate (***29***)*

Methyl phenyl-*H*-phosphinate (**46**) (0.100 g, 0.641 mmol, 1.0 equiv.) and carbon tetrachloride (0.23 mL, 2.422 mmol, 3.8 equiv.) were added to a 7-mL reaction vial under an atmosphere of N_2_. Next, anhydrous CH_2_Cl_2_ (0.3 M solution) was added, and the solution was cooled to 0 °C. A solution of *N,N*-diisopropylethylamine (0.16 g, 1.211 mmol, 2.0 equiv.), benzotetramisole (*S*)-**21** (0.031 g, 0.121 mmol, 0.19 equiv.), and phenol (28.2 mg, 0.300 mmol, 0.47 equiv.) that was previously dried over 4 Å molecular sieves was prepared in anhydrous CH_2_Cl_2_ (0.3 mL). The alcohol solution was added dropwise to the phosphinate solution and stirred for 9 h at 0 °C. The mixture was quenched with 1 M HCl (1 mL) and extracted three times with ethyl acetate then dried with Na_2_SO_4_. The product was purified by silica gel chromatography with ethyl acetate: hexane (50:50) as eluent. The NMR data matched previously published results [16]. Yield 24.6 mg, (33%). ^1^H NMR (400 MHz, CDCl_3_) δ 7.86 (m, 1H), 7.59–7.52 (m, 2H), 7.50–7.42 (m, 1H), 7.29–7.23 (m, 2H), 7.17–7.07 (m, 5H), 3.84 (d, *J* = 11.3 Hz, 1H). ^13^C NMR (101 MHz, CDCl_3_) δ 150.7 (d, *J* = 7.2 Hz), 133.1 (d, *J* = 3.0 Hz), 132.2 (d, *J* = 10.1 Hz), 129.8 (s), 128.8 (d, *J* = 15.5 Hz), 127.1 (d, *J* = 191.0 Hz), 125.1 (s), 120.7 (d, *J* = 4.5 Hz), 53.31 (d, *J* = 3.5 Hz). ^31^P NMR (162 MHz, CDCl_3_) δ 17.39 (s). ${\left[\alpha \right]}_{D}^{20}$ + 19.7° (c 0.14, CHCl_3_). The absolute configuration of compound **29** was determined to be (*R*)-configuration based on the previously published optical rotation of the **29** (${\left[\alpha \right]}_{D}^{20}$ + 38.1° (c 1.45, CHCl_3_)), which was defined using chiral shift reagents [44].

*General procedure for synthesis Ethyl (Aryl)-phenyl-phosphonate*

Ethyl aryl-*H*-phosphinate (**11**) (0.100 g, 0.588 mmol, 1.0 equiv.) and carbon tetrachloride (0.21 mL, 2.170 mmol, 3.7 equiv.) were added to a 7-mL reaction vial under an atmosphere of N_2_. Next, anhydrous CH_2_Cl_2_ (0.3 mL solution) was added, and the solution was cooled to 0 °C. A solution of *N,N*-diisopropylethylamine (0.140 g, 1.100 mmol, 1.9 equiv.), benzotetramisole (*S*)-**21** (0.027 g 0.110 mmol, 0.19 equiv.), and phenol (0.024 g, 0.25 mmol, 0.43 equiv.) that was previously dried over 4 Å molecular sieves was prepared in anhydrous CH_2_Cl_2_ (0.3 mL). The alcohol solution was added dropwise to the phosphinate solution and stirred for 9 h at 0 °C. The mixture was quenched with 1 M HCl (1 mL), extracted three times with ethyl acetate, and then dried with Na_2_SO_4_. The product was purified by silica gel chromatography with ethyl acetate: hexane (30:70) as the eluent. The following products were synthesized.

*(R)-Ethyl (phenyl)-phenyl-phosphonate (***30***)*

Yield 62.3 mg (95%). The NMR data matched with previously published results [16]. ^1^H NMR (400 MHz, CDCl_3_) δ 7.91–7.81 (m, 2H), 7.59–7.51 (m, 1H), 7.50–7.40 (m, 2H), 7.28–7.23 (m, 2H), 7.16–7.07 (m, 3H), 4.29–4.18 (m, 2H), 1.34 (t, *J* = 7.1 Hz, 3H). ^13^C NMR (101 MHz, CDCl_3_) δ 150.7 (d, *J* = 7.0 Hz), 132.9 (s), 132.19 (d, *J* = 10.1 Hz), 128.7 (d, *J* = 15.5 Hz), 127.4 (d, *J* = 485.6 Hz), 126.9 (s), 124.1 (s), 120.7 (d, *J* = 4.5 Hz), 63.0 (d, *J* = 5.6 Hz), 16.5 (d, *J* = 7.3 Hz). ^31^P NMR (162 MHz, CDCl_3_) δ 14.43 (d, *J* = 12.7 Hz). ${\left[\alpha \right]}_{D}^{20}$ + 21.7° (c 0.43, CHCl_3_). The absolute configuration of compound **30** was determined to be the (*R*)-configuration based on the previously published optical rotation of **30** (${\left[\alpha \right]}_{D}^{20}$ + 34.4° (c 2.16, CHCl_3_)), which was defined using chiral shift reagents [44].

*(R)-Ethyl (2-methylphenyl)-phenyl-phosphonate (***31***)*

Yield 46.3 mg (67%). ^1^H NMR (400 MHz, CDCl_3_) δ 7.91–7.82 (m, 2H), 7.29–7.23 (m, 2H), 7.17–7.07 (m, 5H), 4.29–4.18 (m, 2H), 1.33 (td, *J* = 7.1, 0.6 Hz, 3H). ^13^C NMR (101 MHz, CDCl_3_) δ 167.0 (d, *J* = 3.9 Hz), 164.4 (d, *J* = 3.8 Hz), 150.6 (d, *J* = 7.4 Hz), 134.8 (dd, *J* = 11.6, 9.0 Hz), 129.8 (s), 125.1 (s), 120.7 (d, *J* = 4.6 Hz), 116.1 (dd, *J* = 21.5, 16.8 Hz), 63.2 (d, *J* = 6.1 Hz), 16.5 (d, *J* = 6.5 Hz). ^31^P NMR (162 MHz, CDCl_3_) δ 14.78. HRMS (EI) *m/z* [M + H]^+^ calcd for C_15_H_17_O_3_P: 276.09154; found: 276.09189; ${\left[\alpha \right]}_{D}^{20}$ + 17.4 (*c* 0.55, CHCl_3_). The absolute configuration of compound **31** is designated to be the (*R*)-configuration based on the analogous reactivity of **30**.

*(R)-Ethyl (4-methoxyphenyl)- phenyl- phosphonate (***32***)*

Yield 64.3 mg (88%). ^1^H NMR (400 MHz, CDCl_3_) δ 8.01–7.92 (m, 1H), 7.43 (m, 1H), 7.29–7.22 (m, 5H), 7.15 (m, 1H), 7.13–7.07 (m, 1H), 4.24 (m, 2H), 2.65 (d, *J* = 1.7 Hz, 3H), 1.33 (t, *J* = 7.1 Hz, 3H). ^13^C NMR (101 MHz, CDCl_3_) δ 155.1 (d, *J* = 6.9 Hz), 146.4 (d, *J* = 10.4 Hz), 138.6 (d, *J* = 10.6 Hz), 137.3 (s), 135.8 (d, *J* = 15.3 Hz), 134.1 (s), 130.0 (d, *J* = 15.4 Hz), 129.2 (s), 124.8 (d, *J* = 4.4 Hz), 67.7 (d, *J* = 6.0 Hz), 25.8 (d, *J* = 3.4 Hz), 20.7 (d, *J* = 6.4 Hz). ^31^P NMR (162 MHz, CDCl_3_) δ 16.84. HRMS (EI) *m/z* [M + H]^+^ calcd for C_15_H_17_O_4_P: 292.08645; found: 292.08532. ${\left[\alpha \right]}_{D}^{20}$ + 9.7 (*c* 0.6713, CHCl_3_). The absolute configuration of compound **32** is designated to be the (*R*)-configuration based on the analogous reactivity of **30**.

*(R)-Ethyl (4-fluorophenyl)-phenyl-phosphonate (***33***)*

Yield 65.3 mg (93%). ^1^H NMR (400 MHz, CDCl_3_) δ 7.78 (m, 2H), 7.27–7.21 (m, 2H), 7.15–7.04 (m, 3H), 6.97–6.91 (m, 2H), 4.26–4.15 (m, 2H), 3.81 (s, 3H), 1.32 (t, *J* = 7.1 Hz, 3H). ^13^C NMR (101 MHz, CDCl_3_) δ 163.3 (d, *J* = 3.4 Hz), 150.9 (d, *J* = 7.0 Hz), 134.2 (d, *J* = 11.6 Hz), 129.8 (s), 124.9 (s), 120.8 (d, *J* = 4.4 Hz), 119.0 (d, *J* = 198.1 Hz), 114.3 (d, *J* = 16.6 Hz), 62.8 (d, *J* = 5.5 Hz), 55.5 (d, *J* = 2.3 Hz), 16.5 (d, *J* = 6.6 Hz). ^31^P NMR (162 MHz, CDCl_3_) δ 16.88. HRMS (EI)^+^ *m/z* [M + H]^+^: calcd for C_14_H_14_O_3_PF: 280.06646; found: 280.06528. ${\left[\alpha \right]}_{D}^{20}$ + 3.8 (*c* 0.55, CHCl_3_). The absolute configuration of compound **33** is designated to be the (*R*)-configuration based on the analogous reactivity of **30**.

## 4. Conclusions

A dynamic kinetic transformation that produces stereogenic-phosphorus centers using an asymmetric nucleophilic catalyst was developed. Alcohols, both aliphatic and aromatic, were coupled to racemic *H*-phosphinates under halogenation conditions. After the optimization of the solvent, base, temperature, and catalyst loading, benzotetramisole was observed to provide asymmetric phosphonates in acceptable yield (33%–95%) with moderate enantioselectivity (up to 62% *ee*). The reaction conditions were tolerant to a variety of alcohol and *H*-phosphinate substrates. Furthermore, a mechanism of selective phosphorylation was proposed that accounts for the stereoselectivity. Future development of the benzotetramisole catalyst could improve enantioselectivity and enable the synthesis of prodrugs for pharmaceutical or biological application.

## Figures and Tables

**Table 1:**
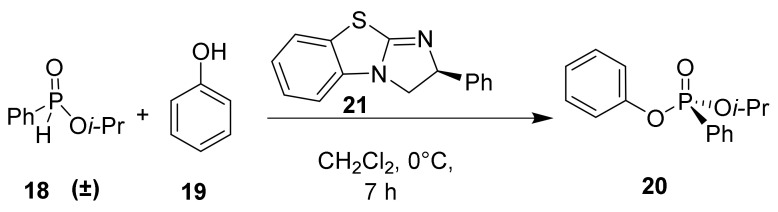
Base effects on the reaction conditions.

Entry ^a^	Base	*pKa in DMSO	Yield (%) ^b^	*ee* (%) ^c^
1	KOtBu	19.2 [40]	63	7
2	DBU	12 [40]	60	20
3	ABCO	9.8 [41]	50	32
4	DABCO	2.97, 8.9 [41,42]	15	33
5	Proton Sponge	7.50 [41]	0	---
6	DTBP	0.81 [43]	0	---
7	DIPEA	9 [40]	58	49

^a^ Conditions: **18** (1 equiv.), CCl_4_ (4 equiv.), PhOH (1 equiv.), Base (2 equiv.), **21** (20 mol%), CH_2_Cl_2_ (0.1 M solution), 7 h; ^b^ yields were determined by HPLC analysis. ^c^ *ee* value was determined by HPLC analysis. KOtBu = Potassium *tert*-butoxide; DBU = 1,8-diazabicyclo [5.4.0] undec-7-ene; ABCO = Quinuclidine; DABCO = 1,4-diazabicyclo[2.2.2]octane; Proton Sponge = 1,8-Bis(dimethylamino)naphthalene; DTBP = Di-*tert*-butyl pyridine; DIPEA = *N,N*-diisopropylethyl amine. *pKa for conjugate acid.

**Table 2:**
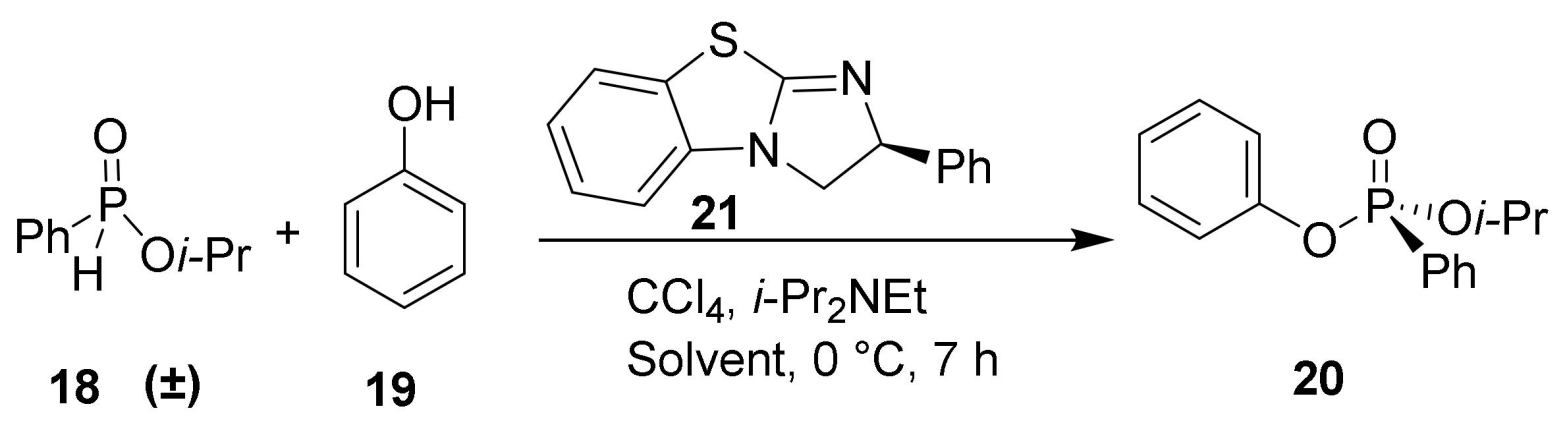
The effects of solvent and temperature on the phosphorylation reaction.

Entry ^a^	Solvent	T (°C)	Yield (%) ^b^	*ee* (%) ^c^
1	THF	0	4	55
2	Toluene	0	17	51
3	CH_3_CN	0	47	26
4	CH_2_Cl_2_	0	58	49
5	CH_2_Cl_2_	25	53 ^d^	45
6	CH_2_Cl_2_	−40	7 ^e^	44

^a^ Conditions: **18** (1 equiv.), CCl_4_ (4 equiv.), PhOH (1 equiv.), *i*-Pr_2_NEt (2 equiv.), **21**. (20%), solvent (0.1 M solution), 7 h; ^b^ yields were determined by HPLC analysis; ^c^*ee* value was determined by HPLC analysis; ^d^ 2hours; ^e^ 16 h.

**Table 3:**
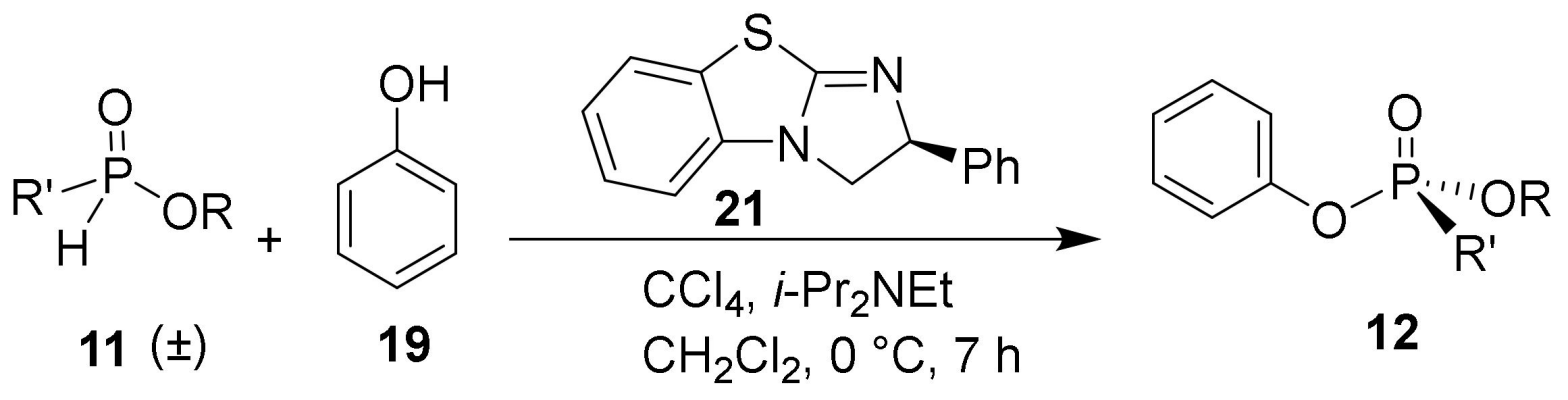
Base effects of electrophilic on phosphorylation reaction.

Entry ^a^	R	R’	Product	Yield (%) ^b^	*ee* (%) ^c^
1	*i*-Pr	Ph	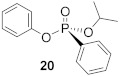	47 40	55 −54 ^d^
2	Me	Ph	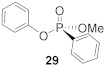	33	61
3	Et	Ph	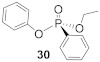	95	62
4	Et	2-MePh	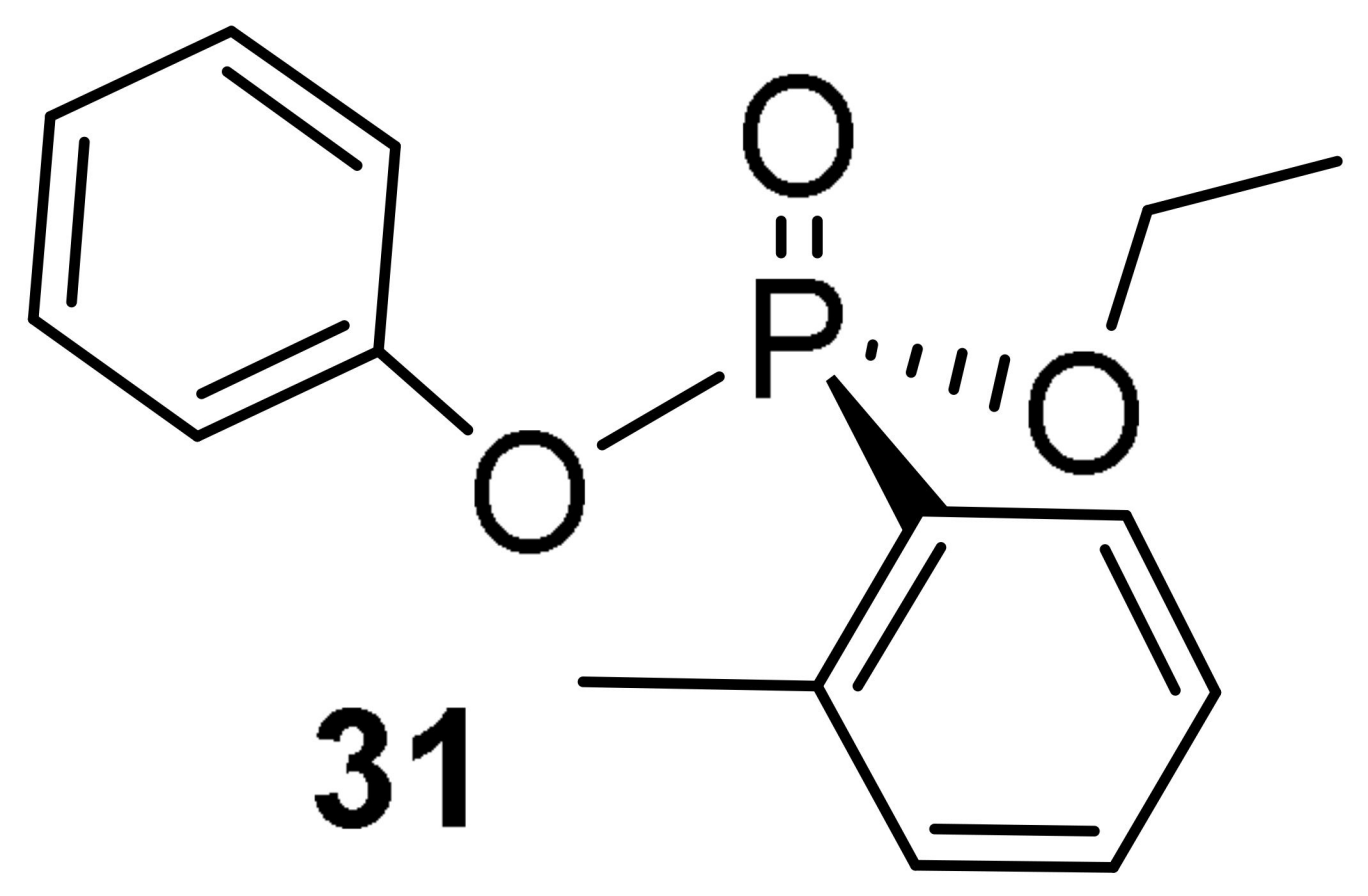	67	48
5	Et	4-OMePh	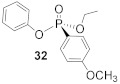	88	22
6	Et	4-F-Ph	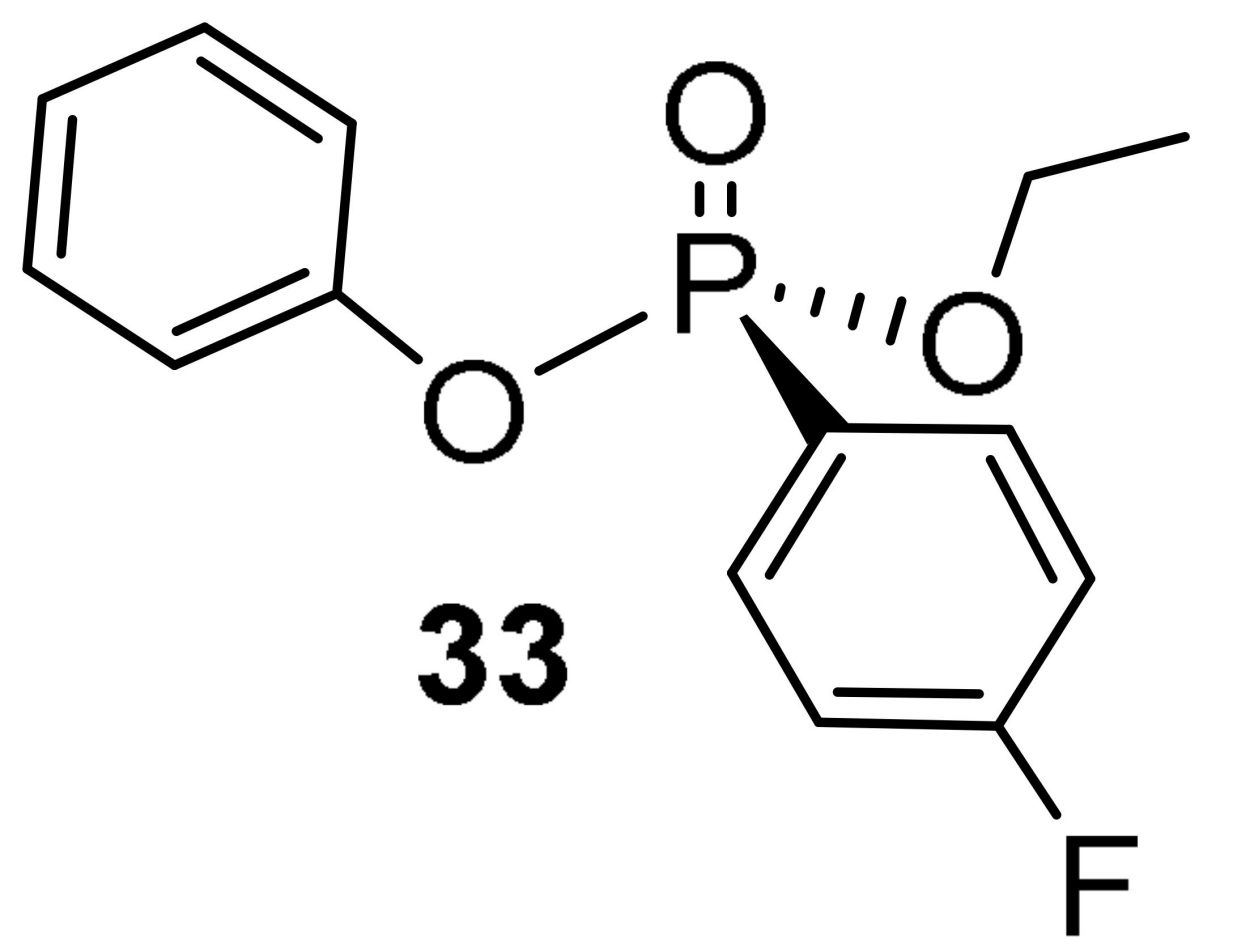	93	22

^a^ Conditions: **18** (1 equiv.), CCl_4_ (4 equiv.), PhOH (0.5 equiv.), *i*-Pr_2_NEt (2 equiv.), **21**. 20 mol%, CH_2_Cl_2_ (0.1 M solution), 7 h; ^b^ Isolated yield; ^c^ *ee* value was determined by HPLC analysis; ^d^ (*R*)-(**21**) was used.

**Table 4:**
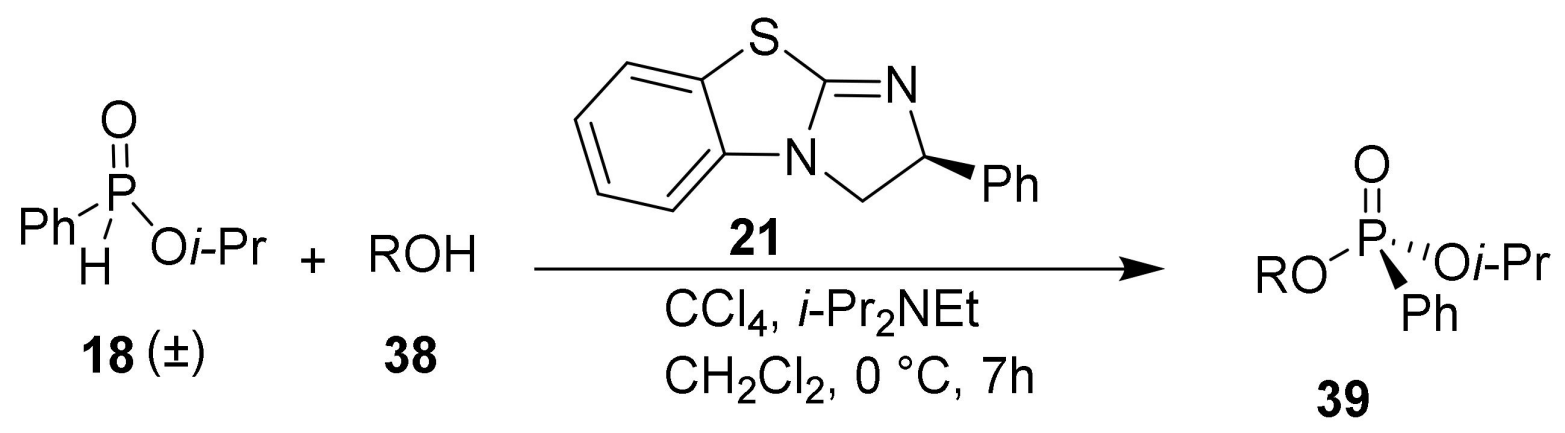
Effects of the nucleophile on the phosphorylation reaction.

Entry ^a^	ROH	Product	Yield (%) ^b^	*ee* (%) ^c^
1	Phenol	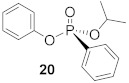	47	55
2	allyl alcohol	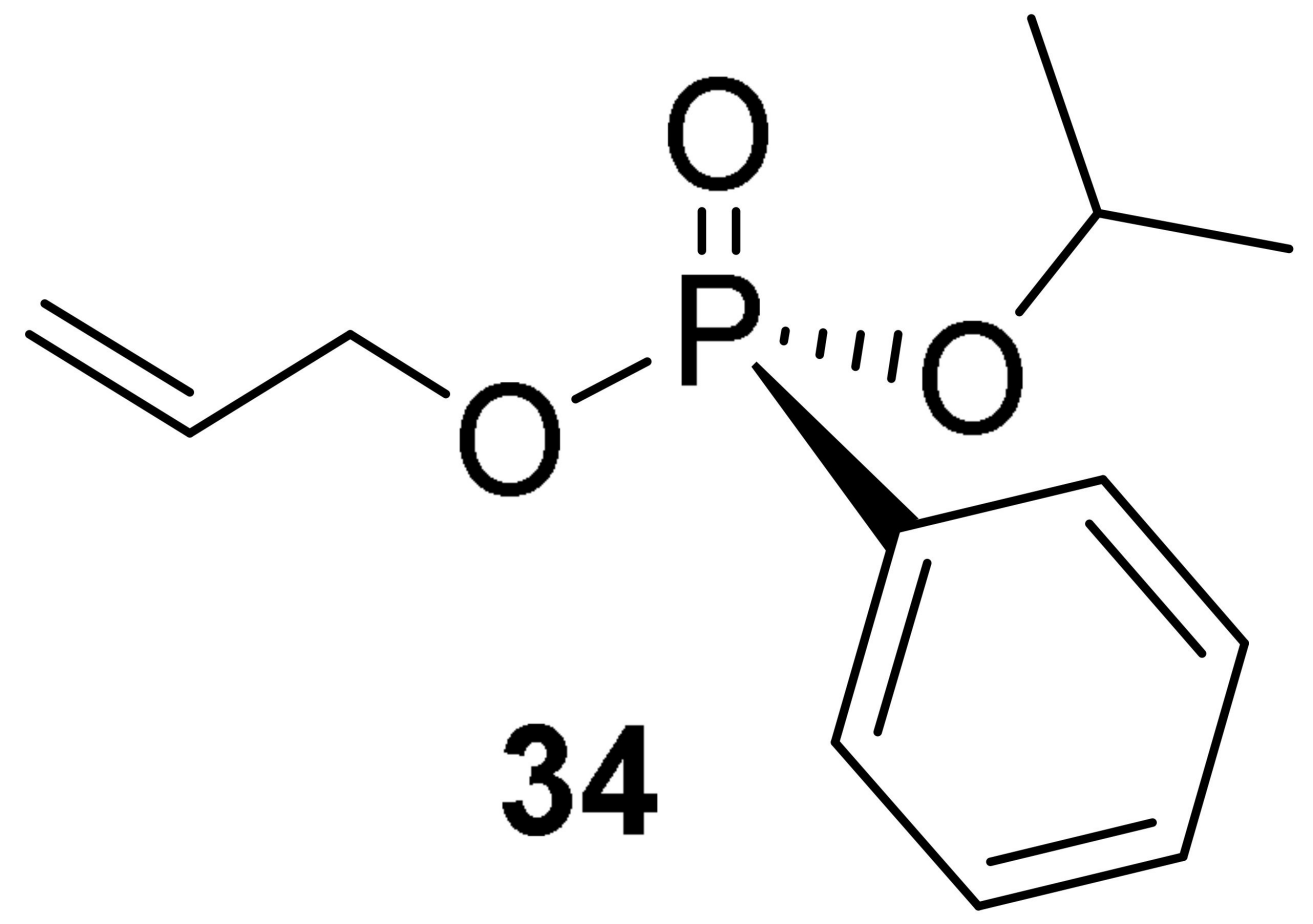	49	43
3	2-phenethyl alcohol	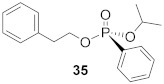	68	28
4	Benzyl alcohol	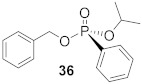	51	35
5	Methanol	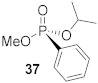	56	51

^a^ Conditions: **18** (1 equiv.), CCl_4_ (4 equiv.), alcohols (0.5 equiv.), *i*-Pr_2_NEt (2 equiv.), **21** (20 mol%), CH_2_Cl_2_ (0.1 M solution), 7h; ^b^ Isolated yield; ^c^ *ee* was determined by HPLC analysis.

## Data Availability

The following are available on at the Supplementary Materials. (1) ^1^H, ^13^C, and ^31^P NMR spectra; (2) HPLC chromatograms; and mass spectra of the obtained compounds.

## Supplementary Materials

The following are available online. Copies of the ^1^H, ^13^C, and ^31^P NMR spectra for all compounds are available online. HPLC of racemic, chiral products and HRMS of novel compounds (**31**–**36**) are also available online.

## Sample Availability

Samples of the compounds (**20**, **29**–**37**) are available from the authors.
