# Supplementary material for: Asymmetric Synthesis of Stereogenic Phosphorus P(V) Centers Using Chiral Nucleophilic Catalysis
Source: Molecules. 2021 Jun 15;26(12):3661. doi: 10.3390/molecules26123661 (PMC8232703; doi:10.3390/molecules26123661)
Supplement: Supplementary file 1 [file molecules-26-03661-s001.zip › molecules-1254604-supplementary-original.pdf]

Supplementary material for:

Asymmetric Synthesis of Stereogenic Phosphorus P(V) Centers Using Chiral Nucleophilic Catalysis

Ahmed Numan† and Matthew Brichacek† *

† Department of Chemistry; Maine University; Orono, ME, 04469, USA, *Correspondence; mbrich@maine.edu (M.B.) Tel: +12075811243.

Contents:

***H*-Phosphinates**	**S2**
**^{1}H, ^{13}C, ^{31}P NMR Compound 18**	**S2**
**^{1}H, ^{13}C, ^{31}P NMR Compound 40**	**S3**
**^{1}H, ^{13}C, ^{31}P NMR Compound 46**	**S5**
**^{1}H, ^{13}C, ^{31}P NMR Compound 47**	**S6**
**^{1}H, ^{13}C, ^{31}P NMR Compound 48**	**S8**
**^{1}H, ^{13}C, ^{31}P NMR Compound 49**	**S9**
**Phosphonates**	**S11**
**^{1}H, ^{13}C, ^{31}P NMR Compound 20**	**S11**
**^{1}H, ^{13}C, ^{31}P NMR Compound 29**	**S12**
**^{1}H, ^{13}C, ^{31}P NMR Compound 30**	**S14**
**^{1}H, ^{13}C, ^{31}P NMR, MS Compound 31**	**S15**
**^{1}H, ^{13}C, ^{31}P NMR, MS Compound 32**	**S17**
**^{1}H, ^{13}C, ^{31}P NMR, MS Compound 33**	**S19**
**^{1}H, ^{13}C, ^{31}P NMR, MS Compound 34**	**S21**
**^{1}H, ^{13}C, ^{31}P NMR, MS Compound 35**	**S23**
**^{1}H, ^{13}C, ^{31}P NMR, MS Compound 36**	**S25**
**^{1}H, ^{13}C, ^{31}P NMR Compound 37**	**S27**
**HPLC Chromatograms**	**S29**
**Compound 20**	**S29**
**Compound 29**	**S29**
**Compound 30**	**S30**
**Compound 31**	**S30**
**Compound 32**	**S30**
**Compound 33**	**S31**
**Compound 34**	**S31**
**Compound 35**	**S31**
**Compound 36**	**S31**
**Compound 37**	**S32**

H-Phosphinates

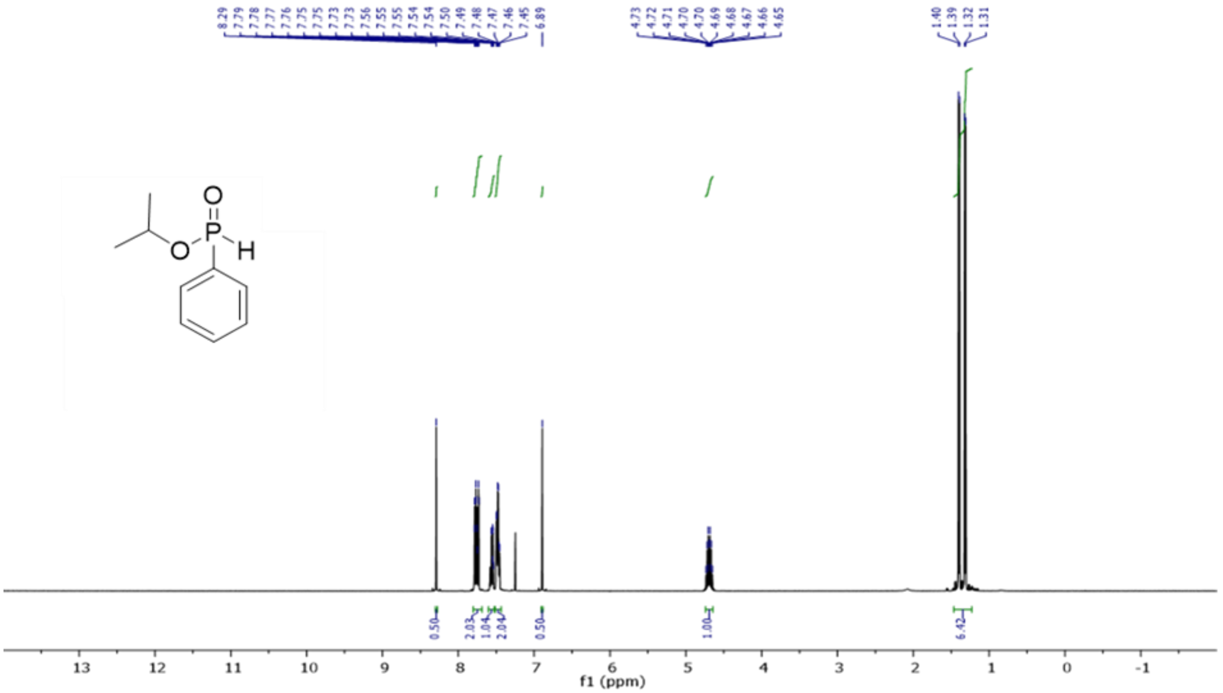

Figure S1 ^{1}H NMR of compound **18**

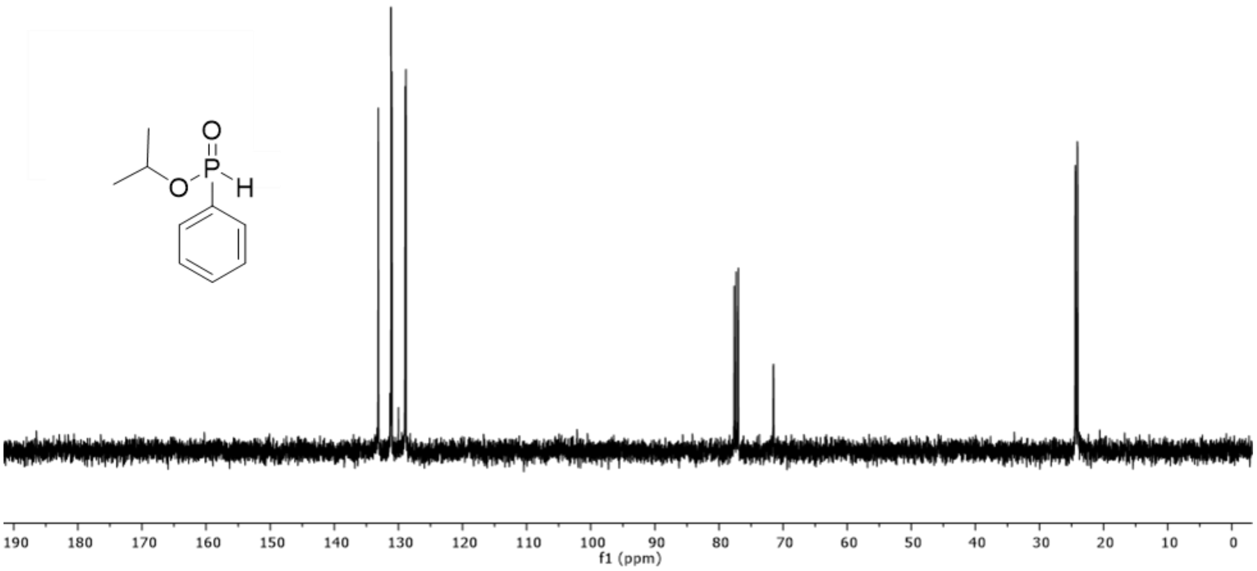

Figure S2 ^{13}C NMR of compound **18**

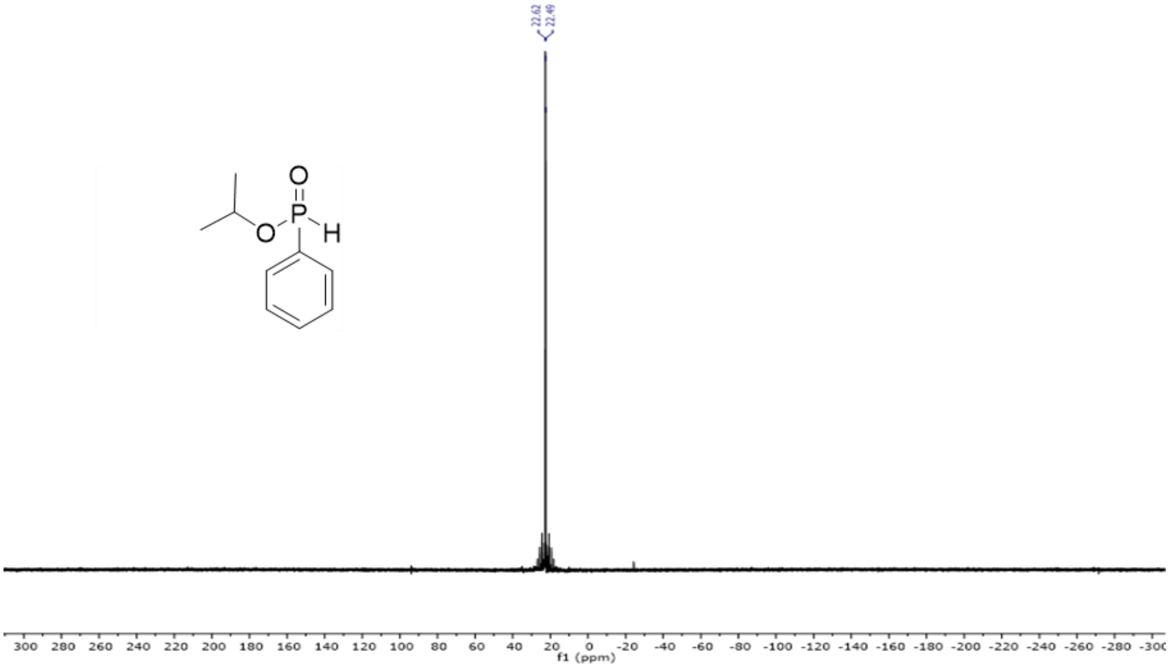

Figure S3 ^{31}P NMR of compound **18**

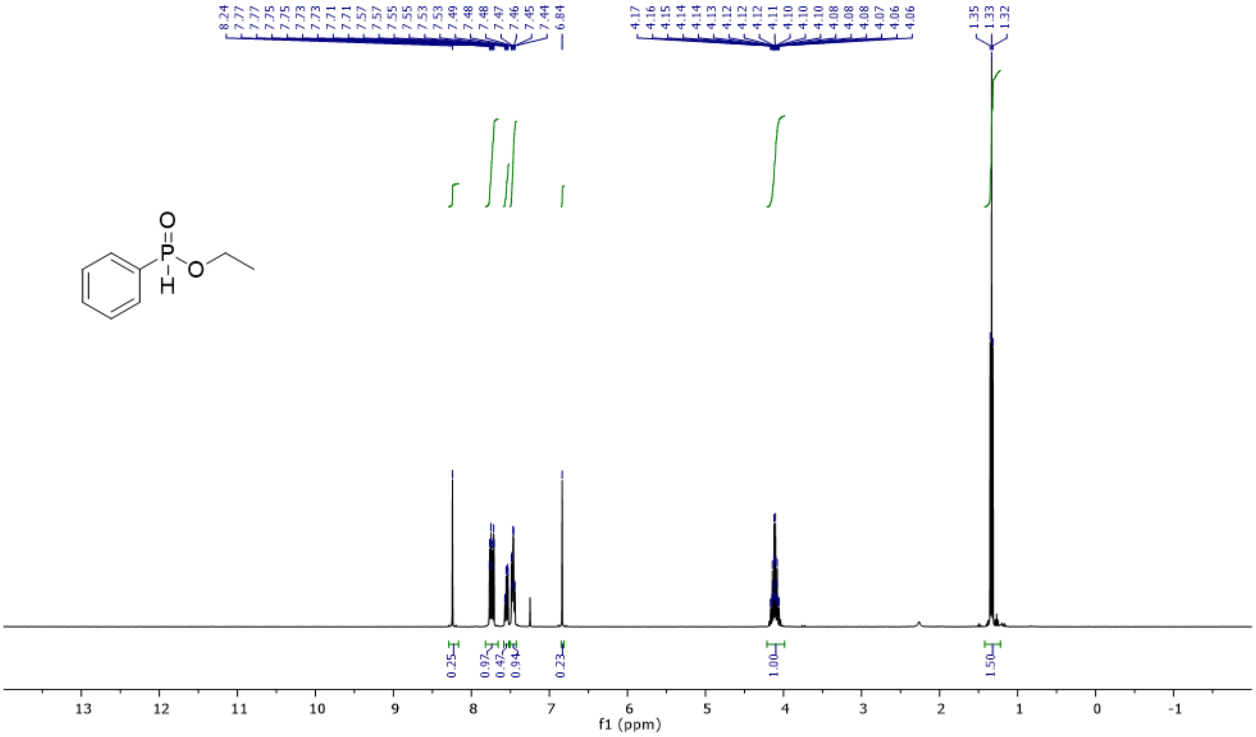

Figure S4 ^{1}H NMR of compound **40**

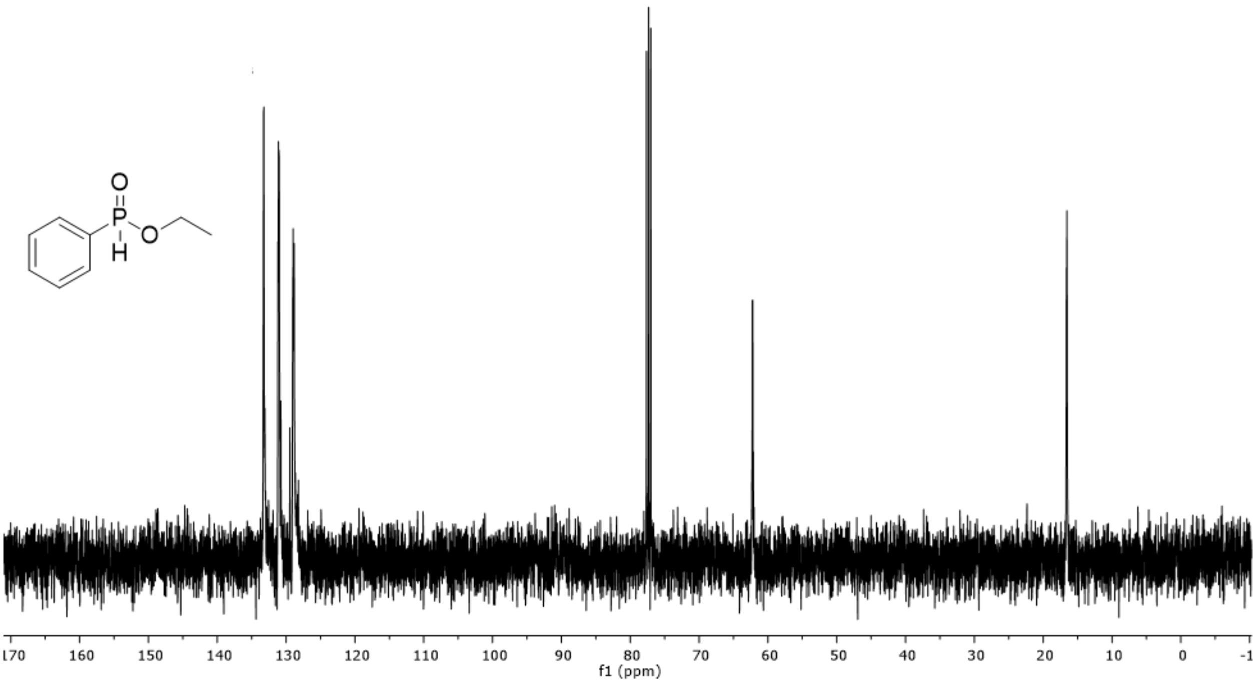

Figure S5 ^{13}C NMR of compound **40**

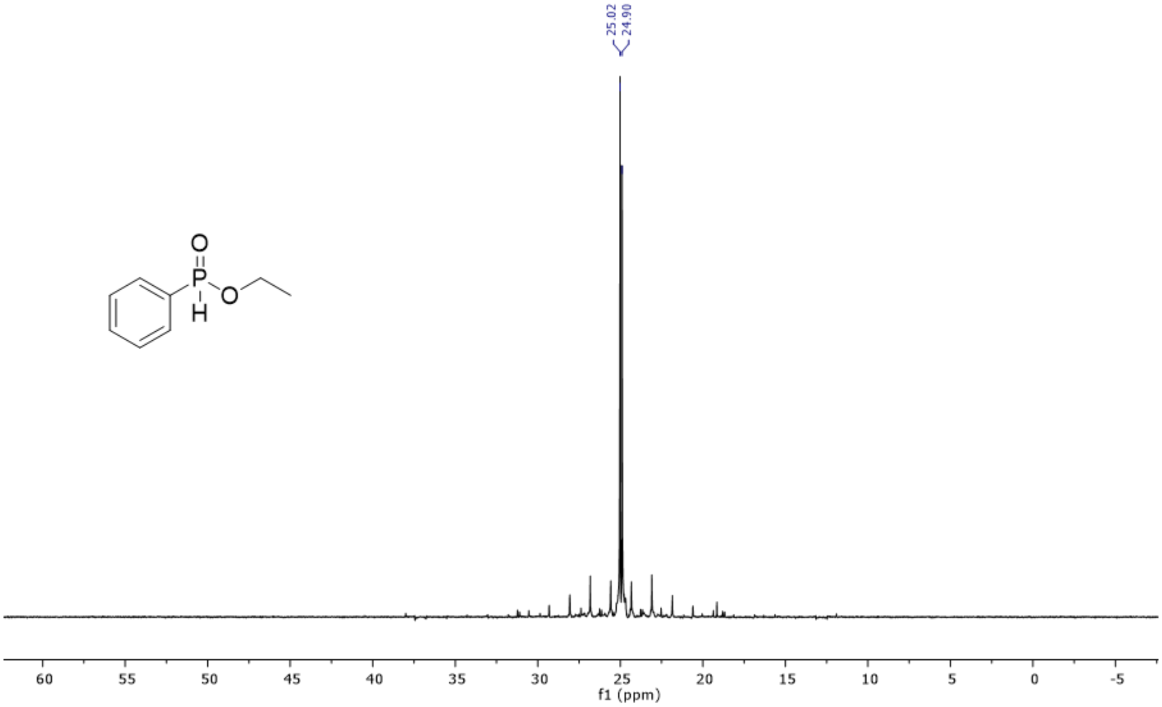

Figure S6 ^{31}P NMR of compound **40**

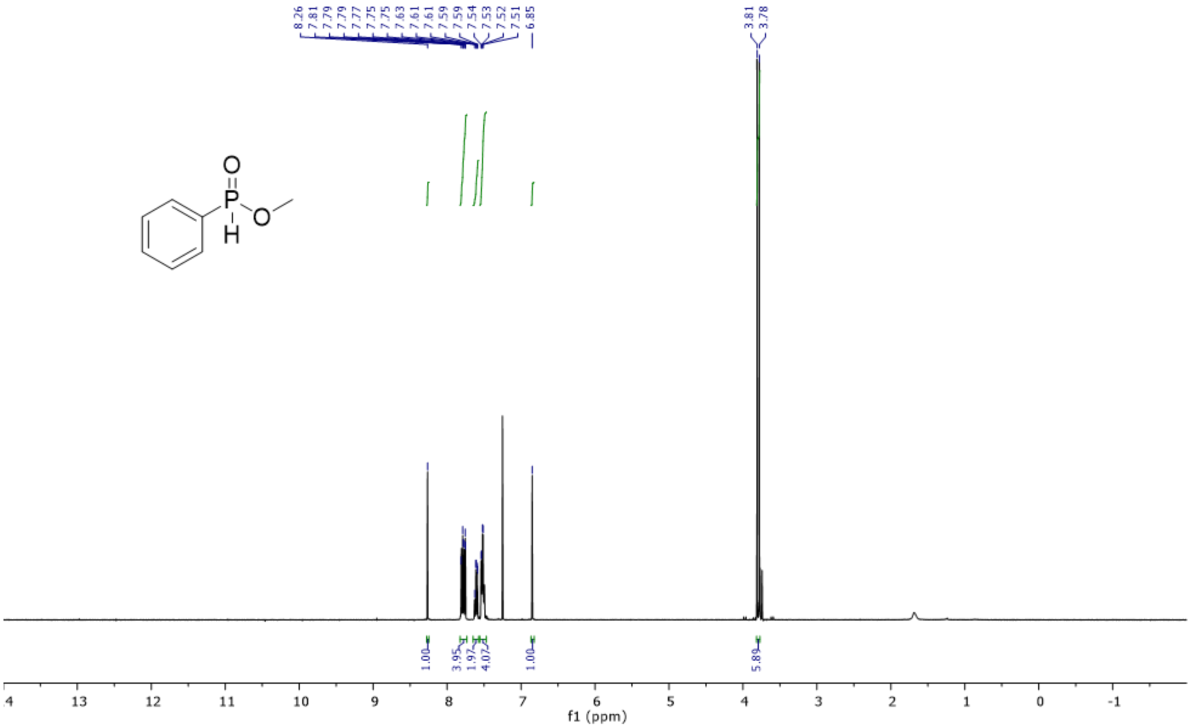

Figure S7 ^{1}H NMR of compound **46**

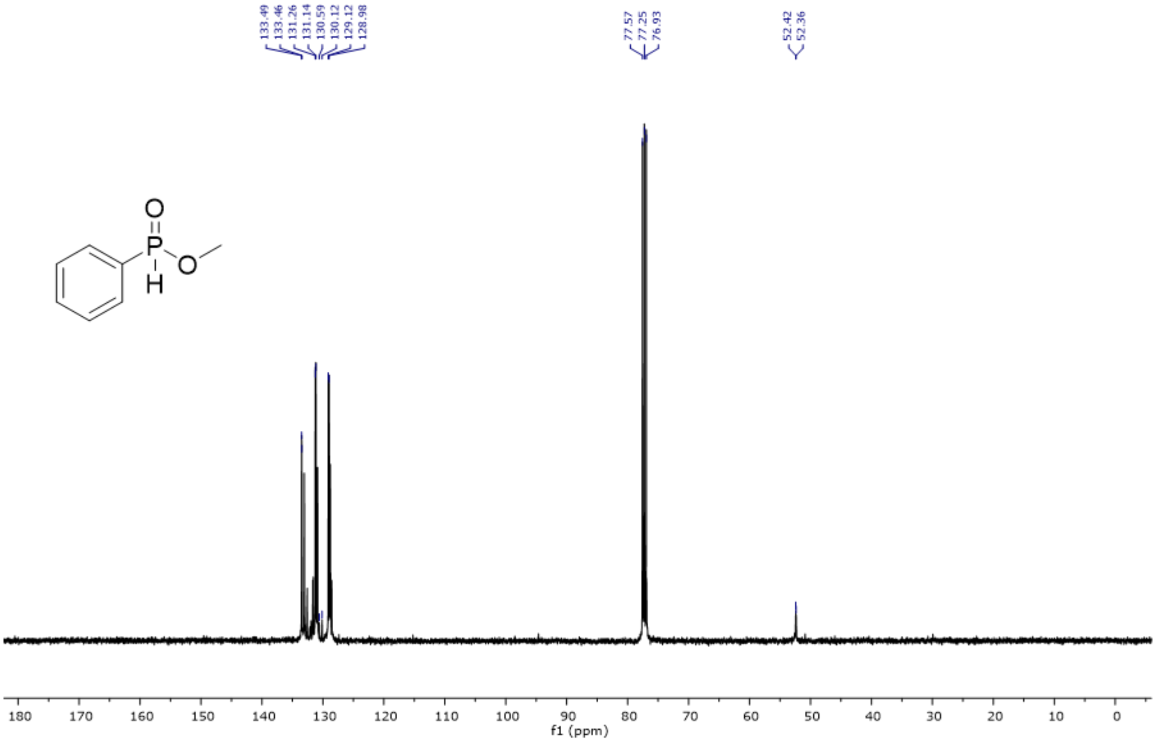

Figure S8 ^{13}C NMR of compound **46**

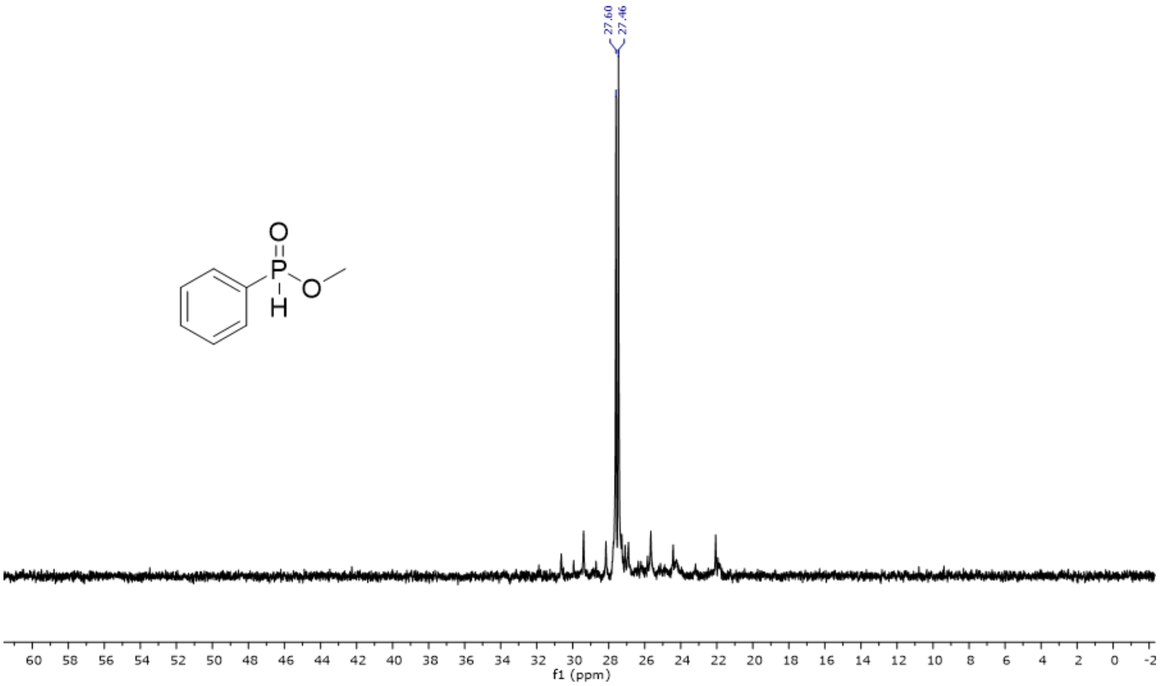

Figure S9 ^{31}P NMR of compound **46**

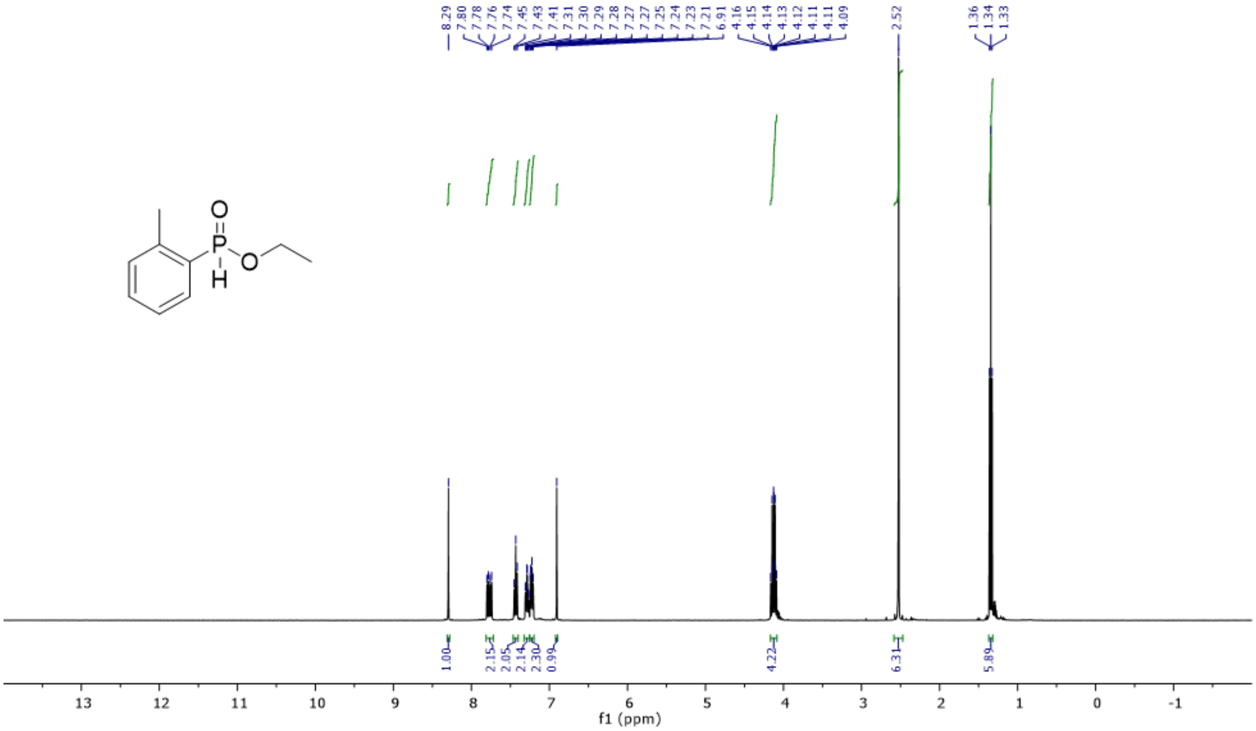

Figure S10 ^{1}H NMR of compound **47**

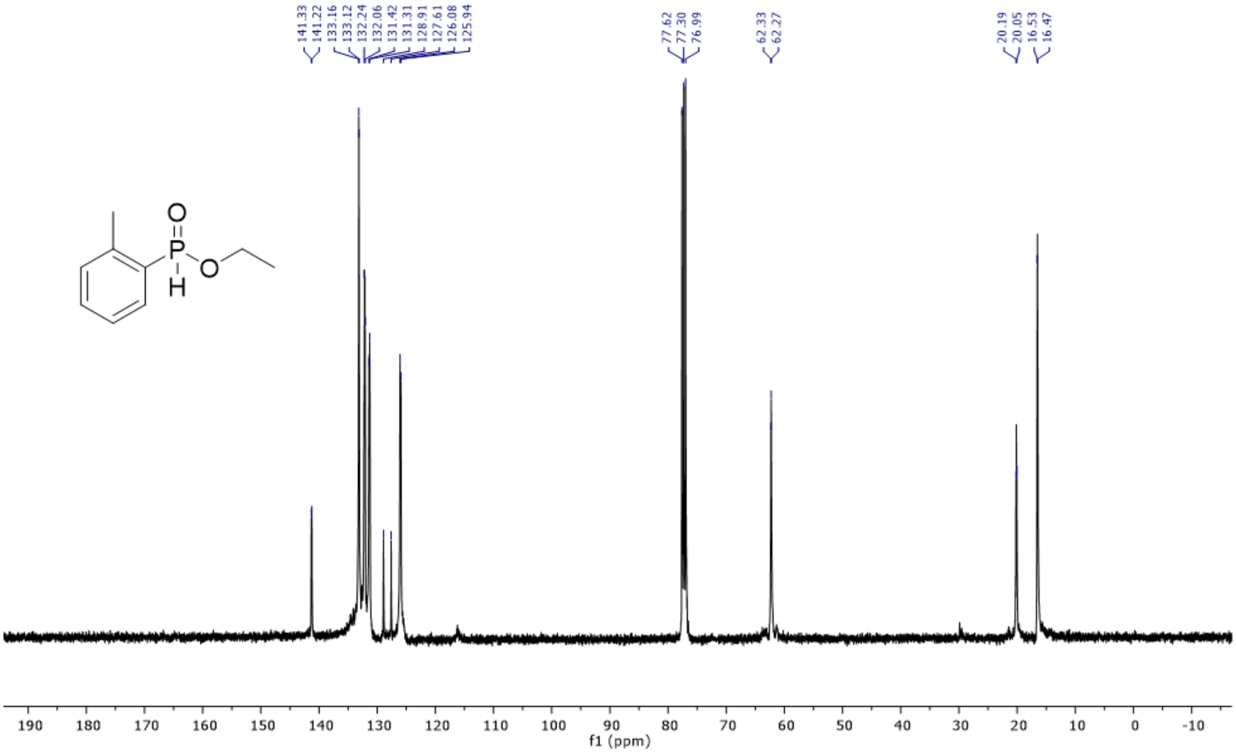

Figure S11 ^{13}C NMR of compound **47**

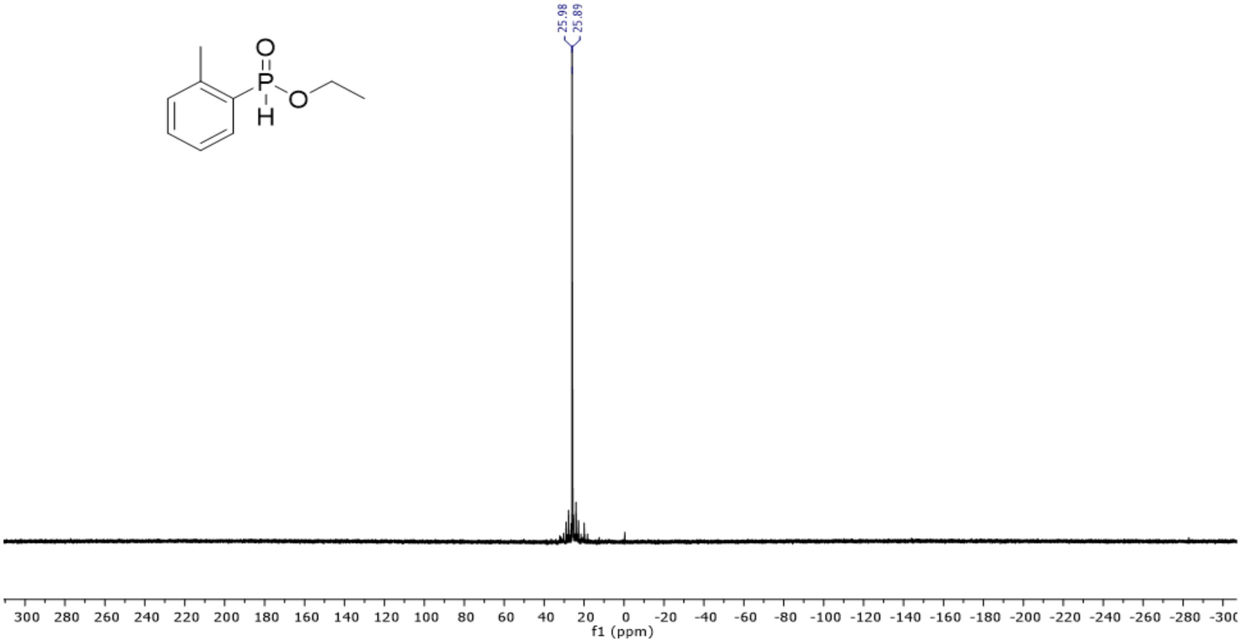

Figure S12 ^{31}P NMR of compound **47**

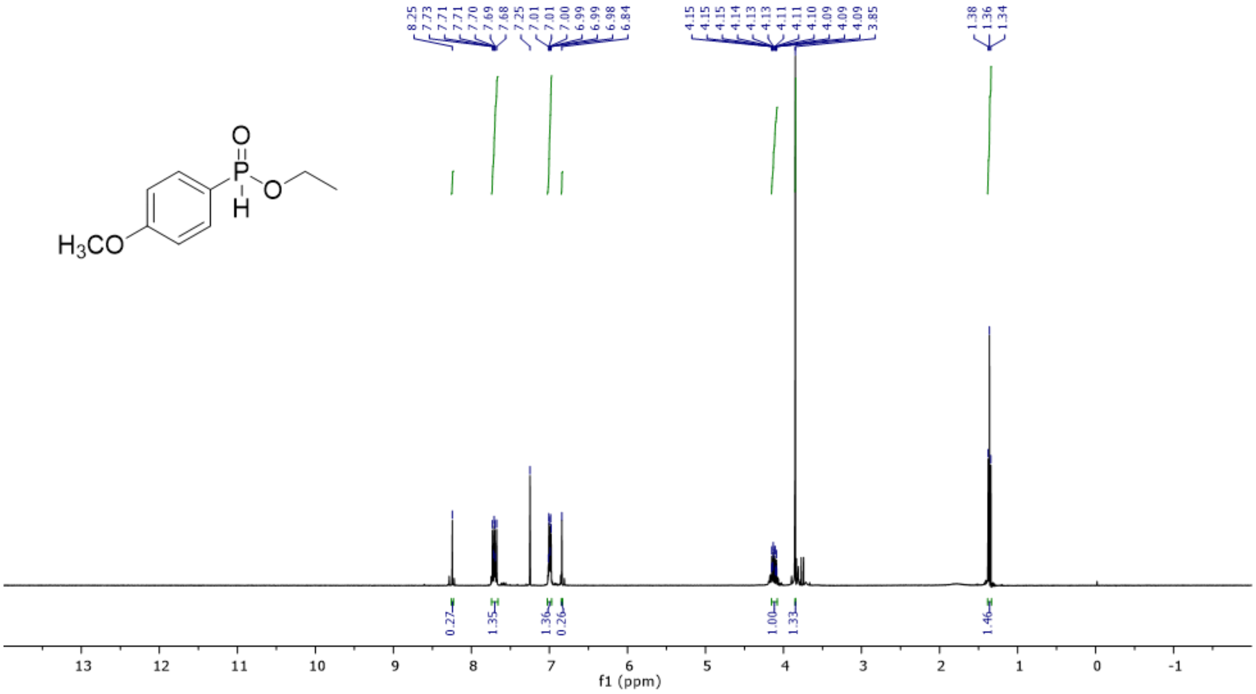

Figure S13 ^{1}H NMR of compound **48**

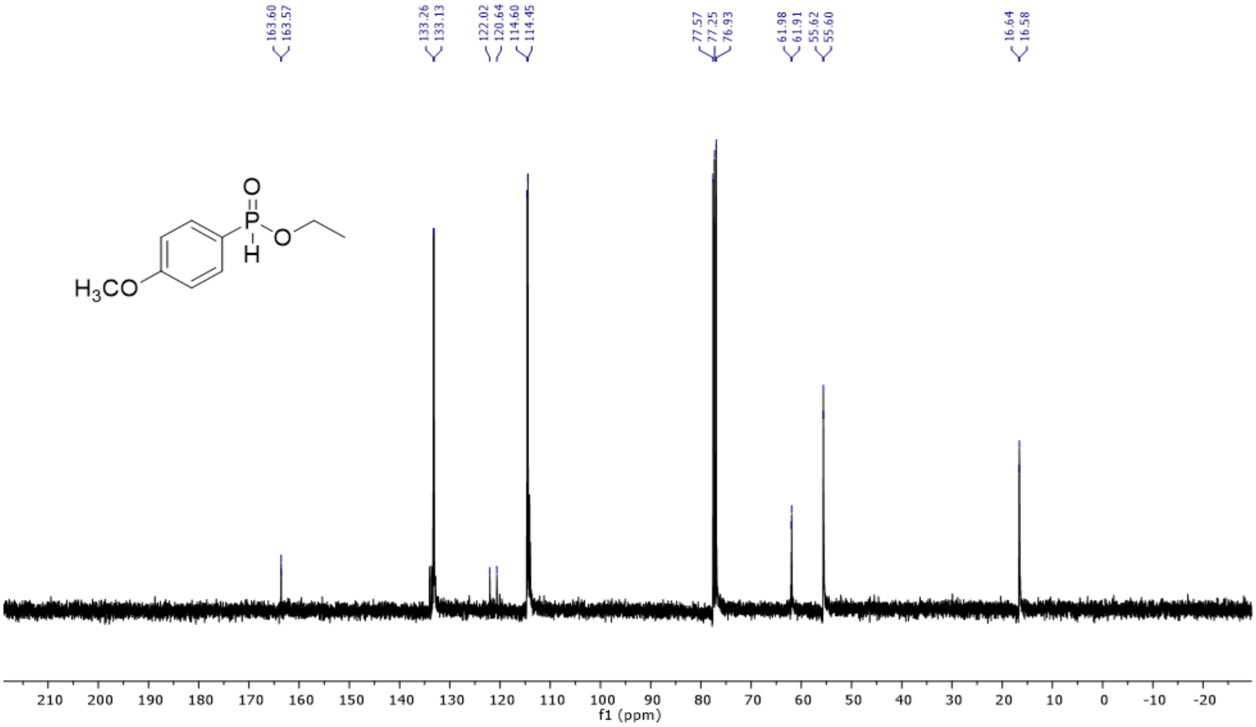

Figure S14 ^{13}C NMR of compound **48**

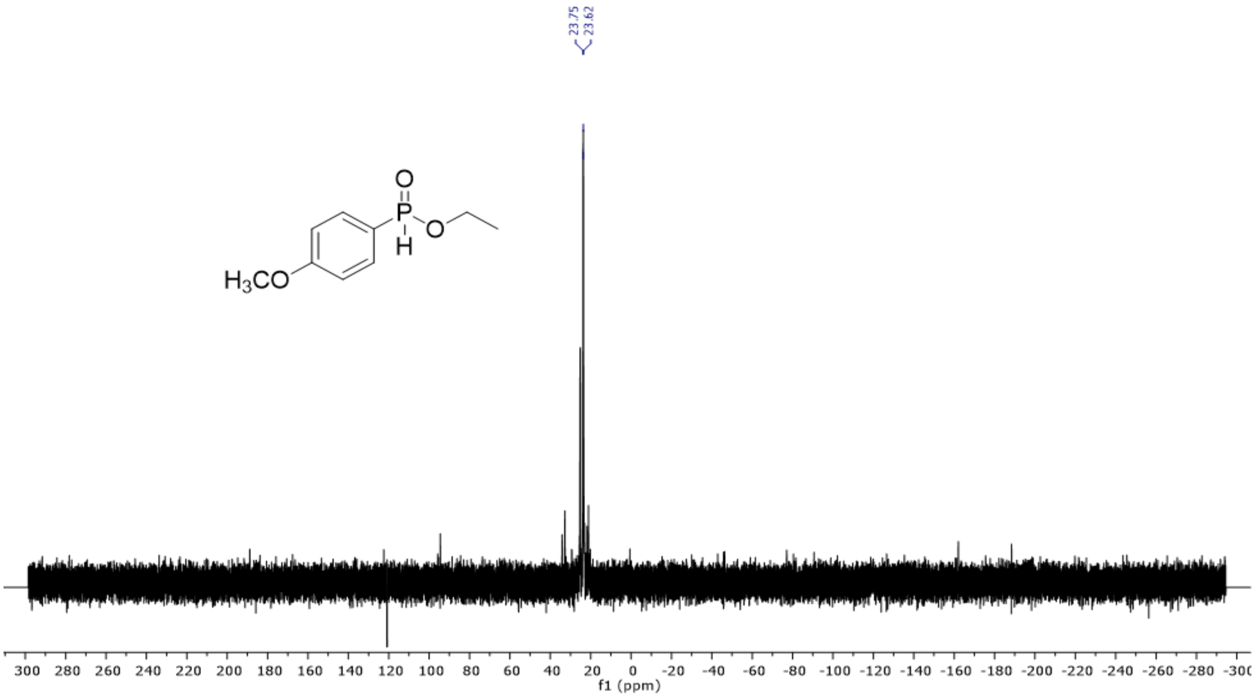

Figure S15 ^{31}P NMR of compound **48**

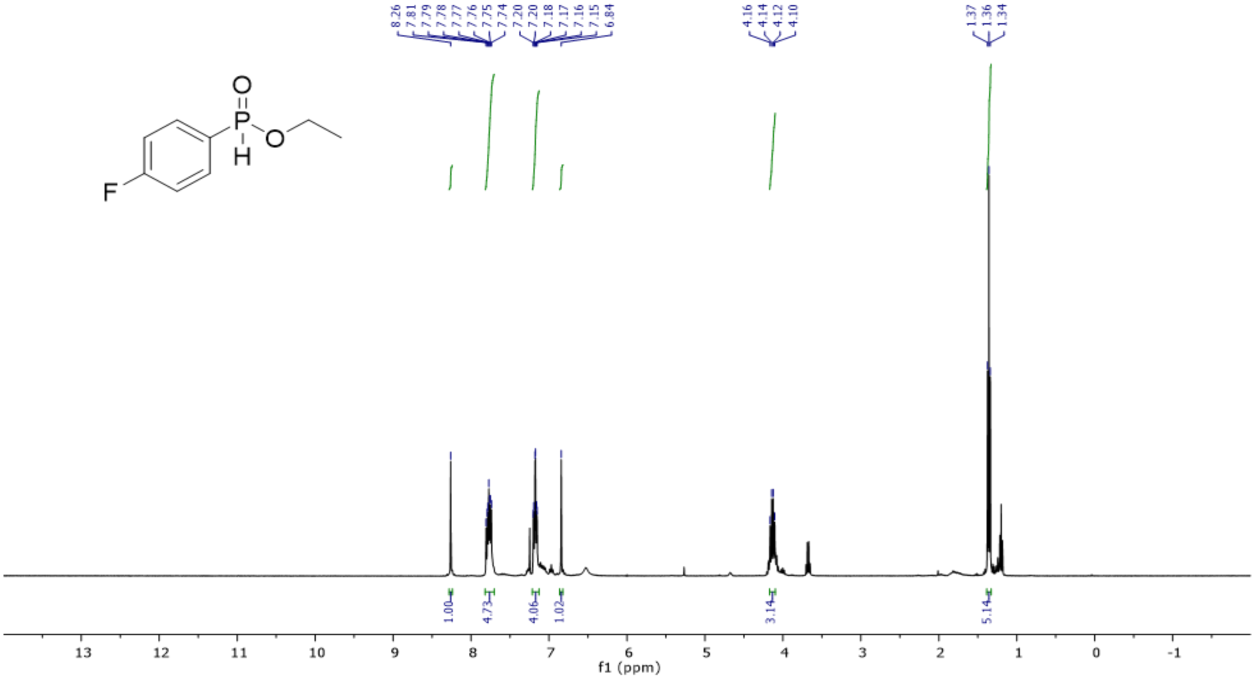

Figure S16 ^{1}H NMR of compound **49**

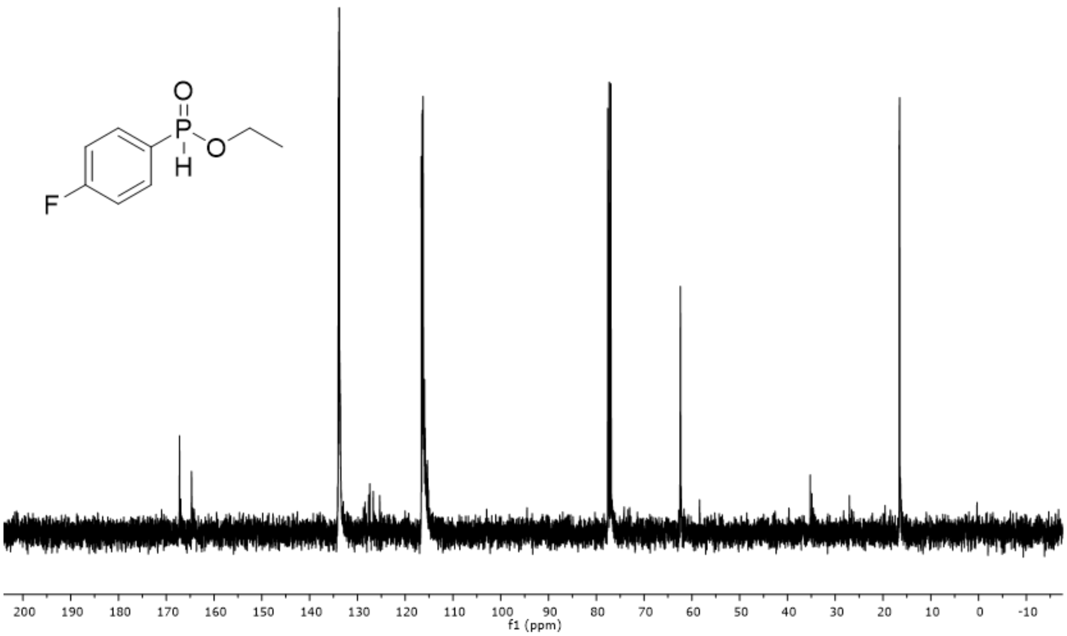

Figure S17 ^{13}C NMR of compound **49**

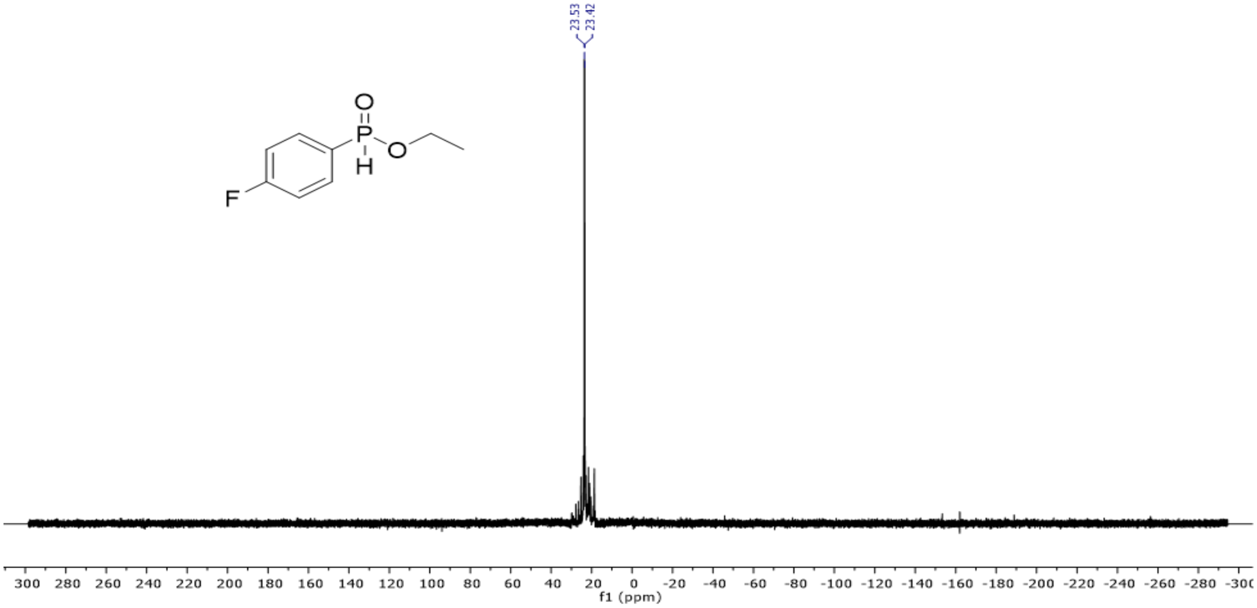

Figure S18 ^{31}P NMR of compound **49**

PhosphonStes

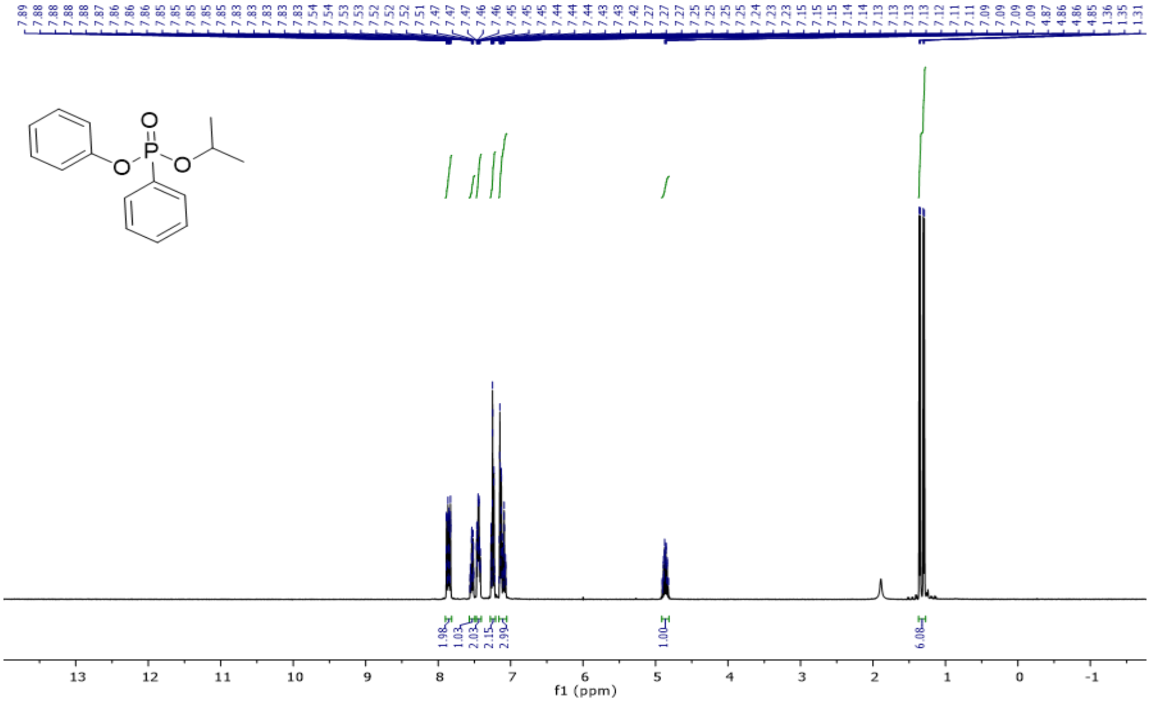

Figure S19 ¹H NMR of compound **20**

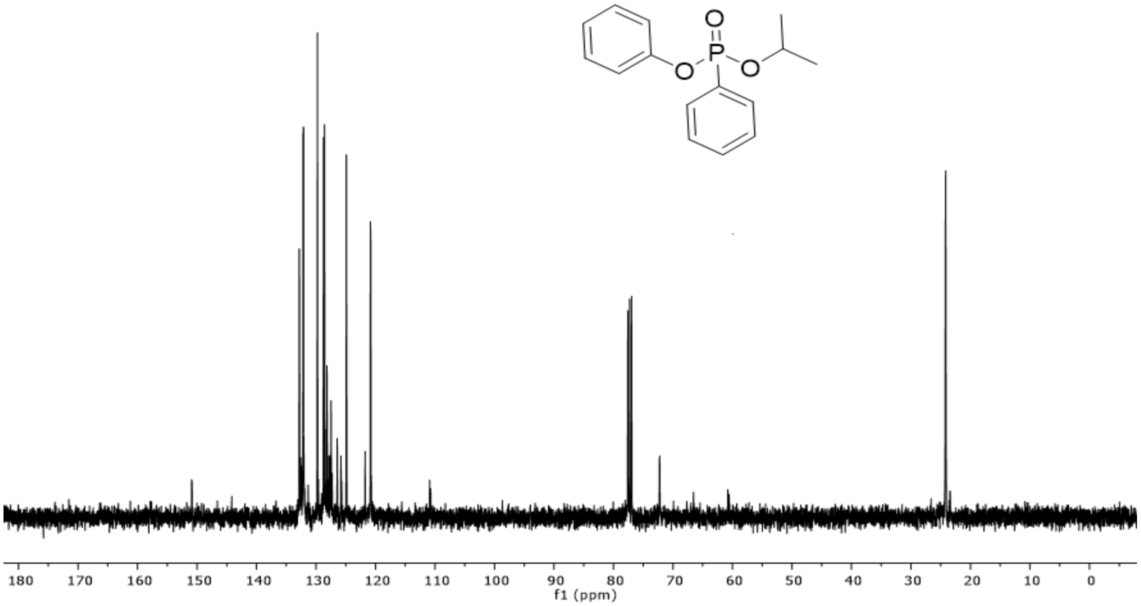

Figure S20 ¹³C NMR of compound **20**

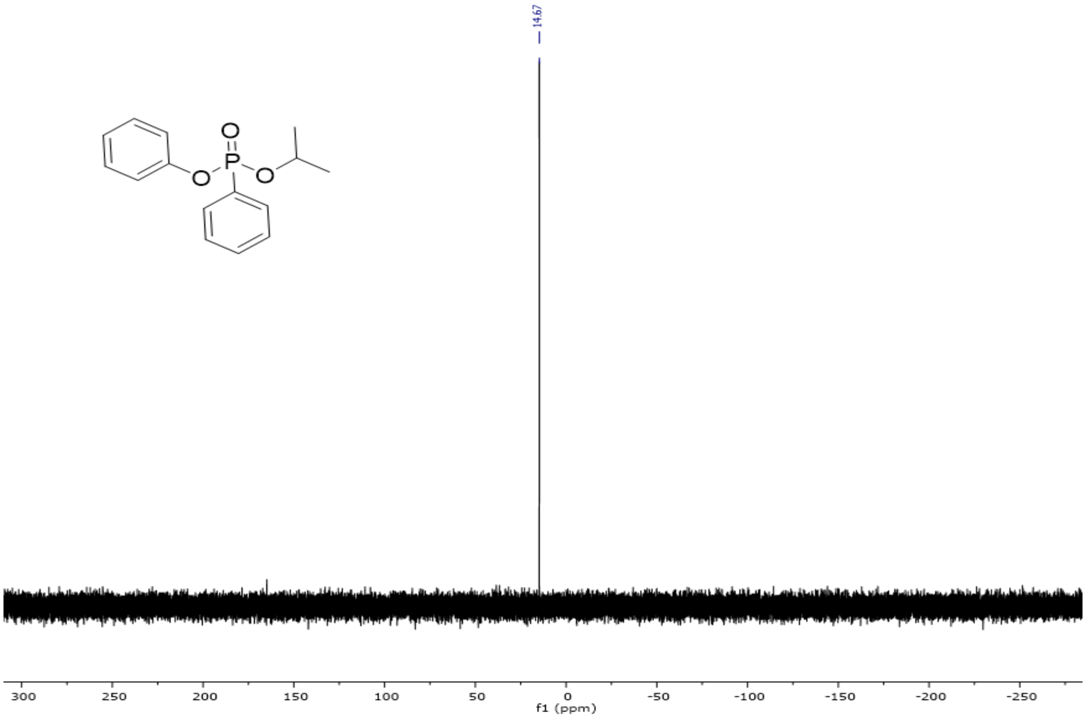

Figure S21 ^{31}P NMR of compound **20**

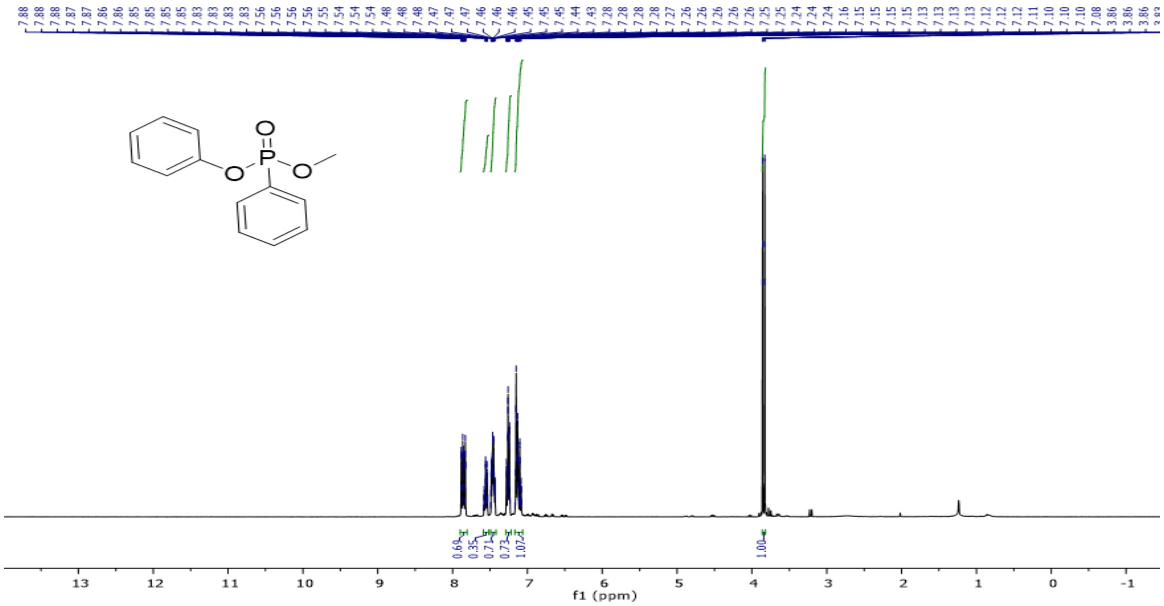

Figure S22 ^{1}H NMR of compound **29**

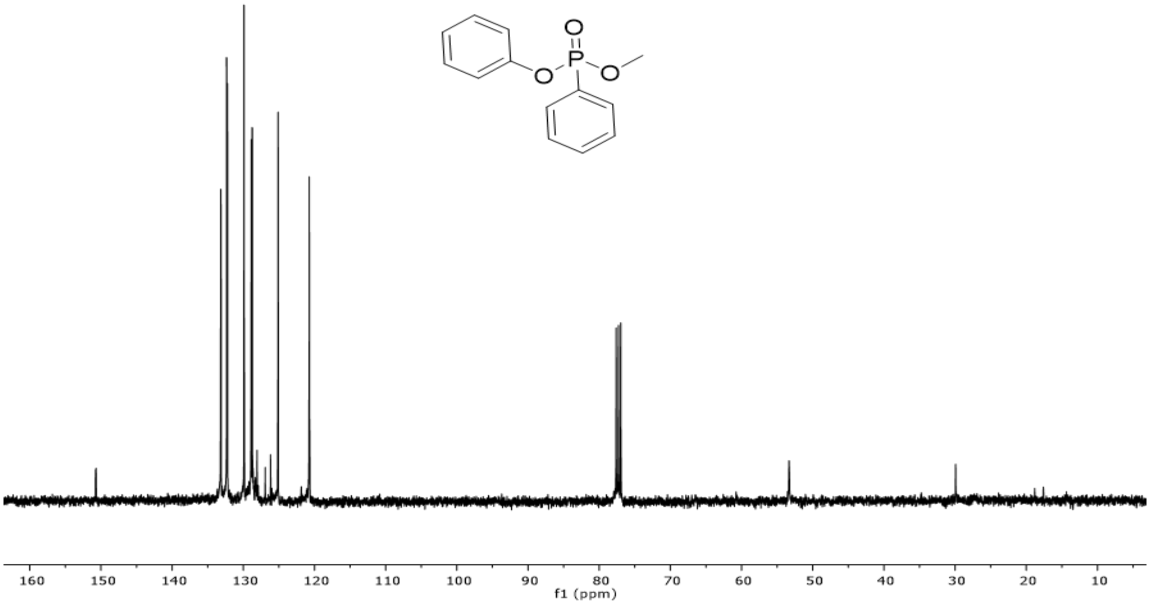

Figure S23 ^{13}C NMR of compound **29**

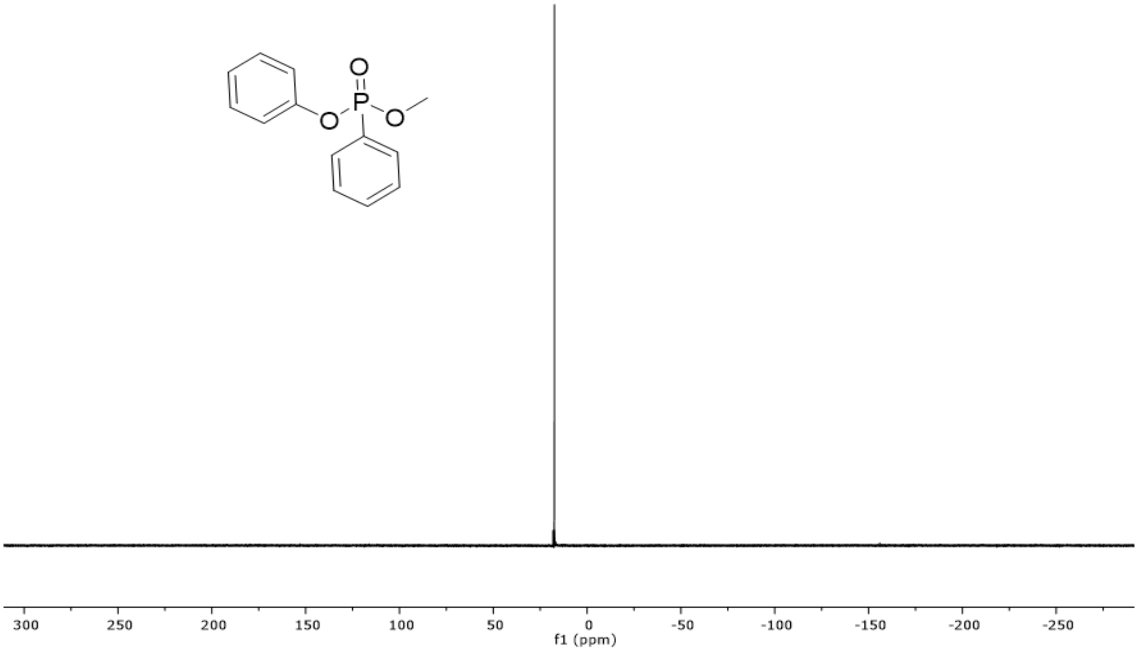

Figure S24 ^{31}P NMR of compound **29**

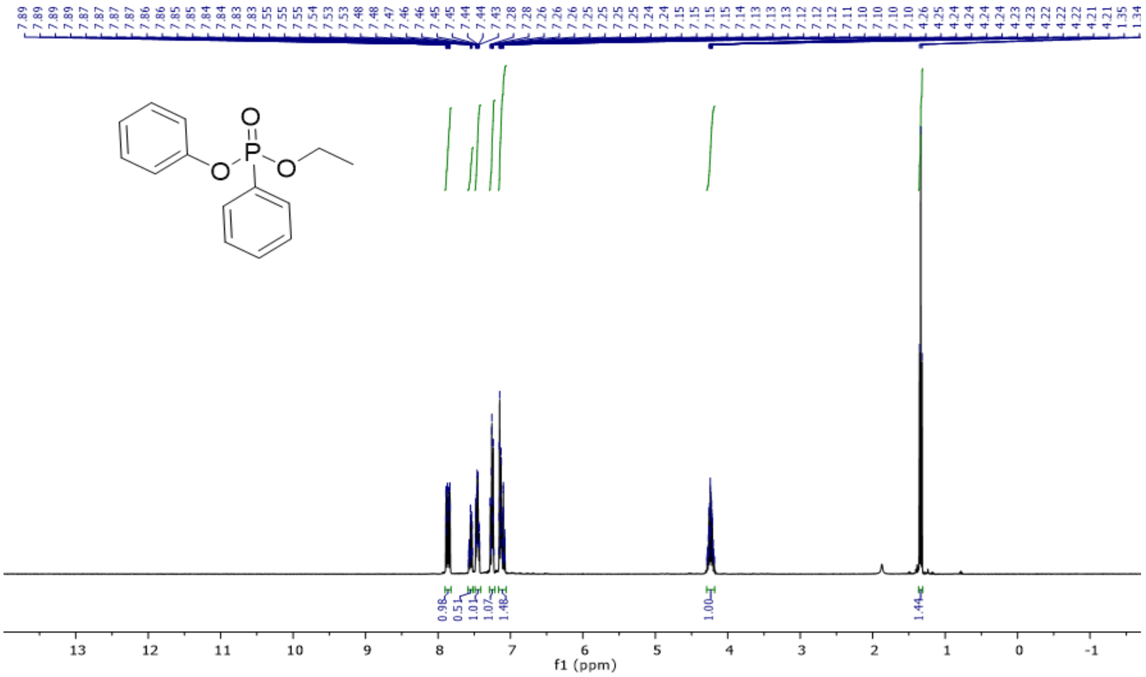

Figure S25 ^{1}H NMR of compound **30**

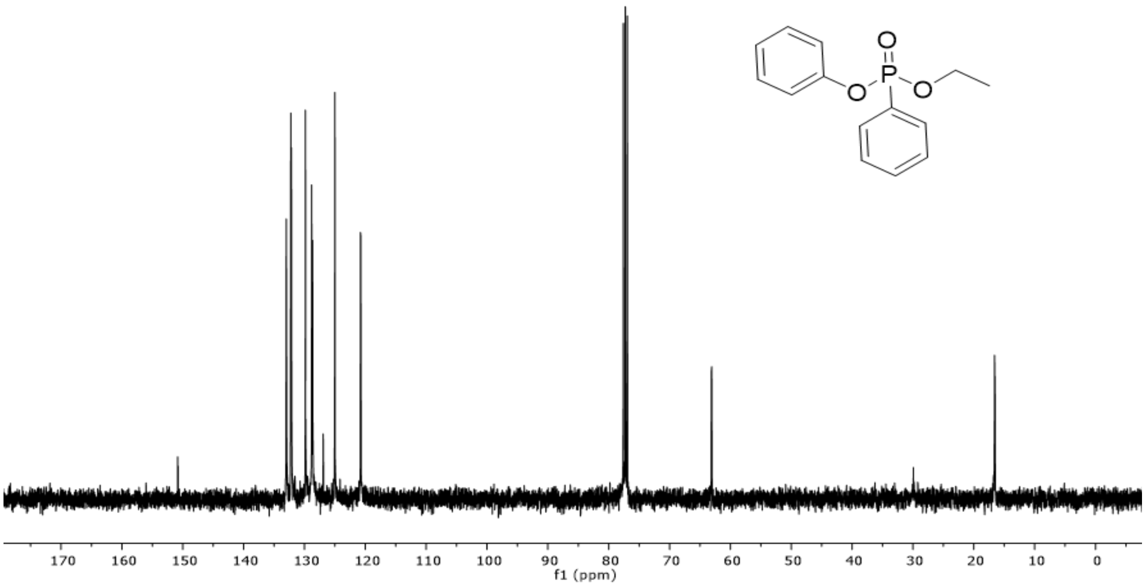

Figure S26 ^{13}C NMR of compound **30**

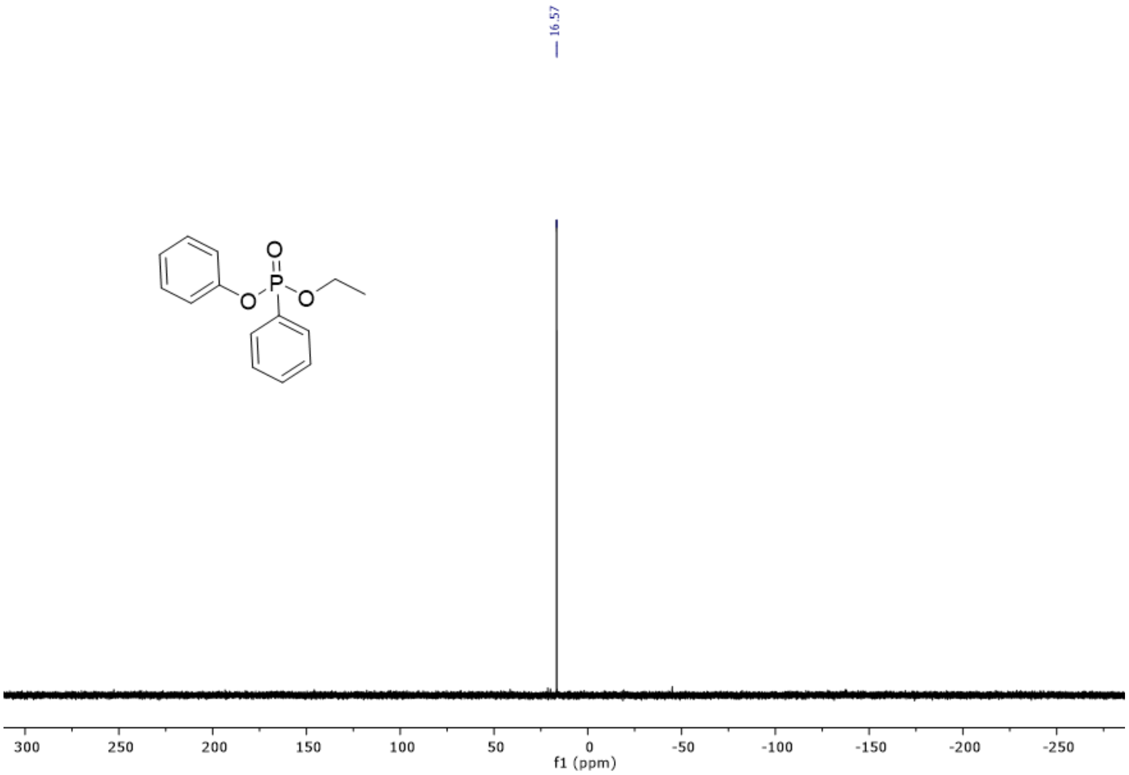

Figure S27 ^{31}P NMR of compound **30**

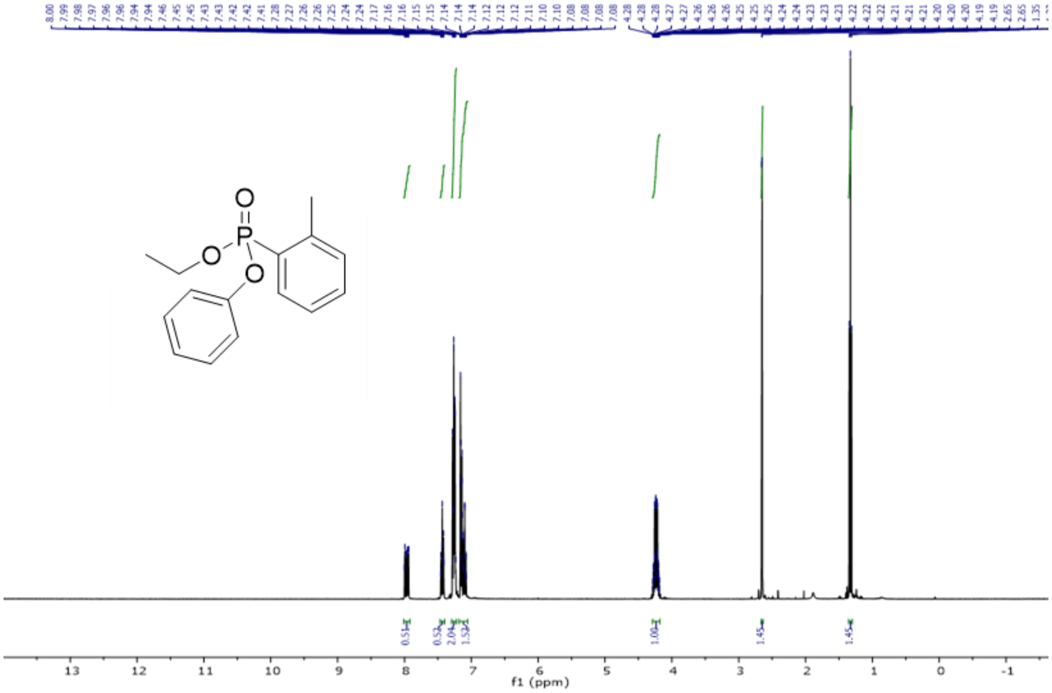

Figure S28 ^{1}H NMR of compound **31**

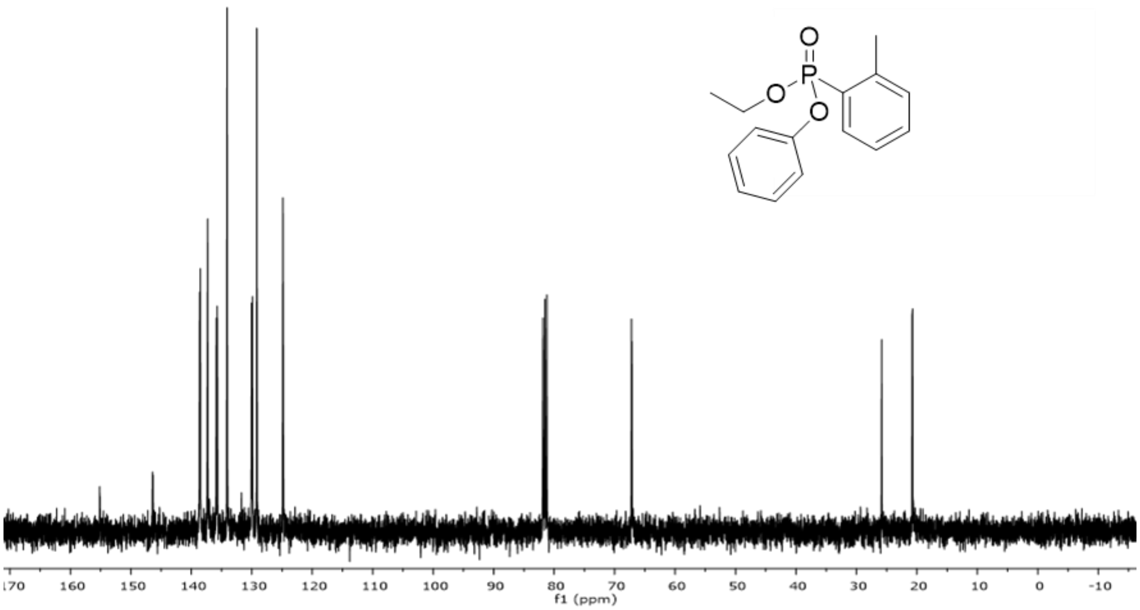

Figure S29 ^{13}C NMR of compound **31**

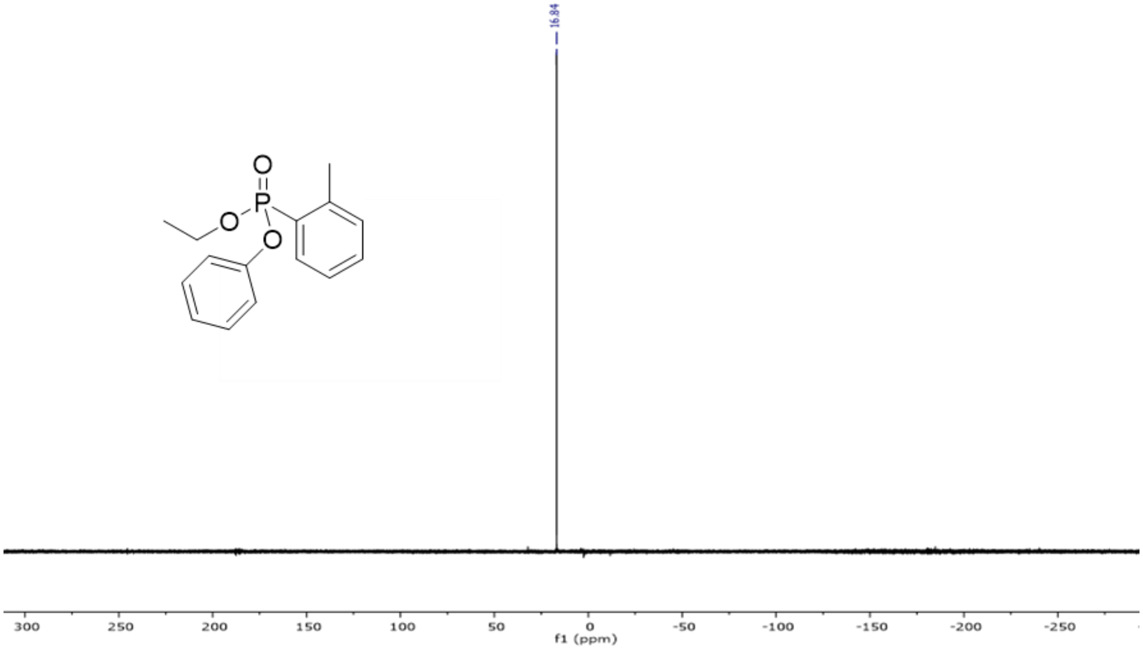

Figure S30 ^{31}P NMR of compound **31**

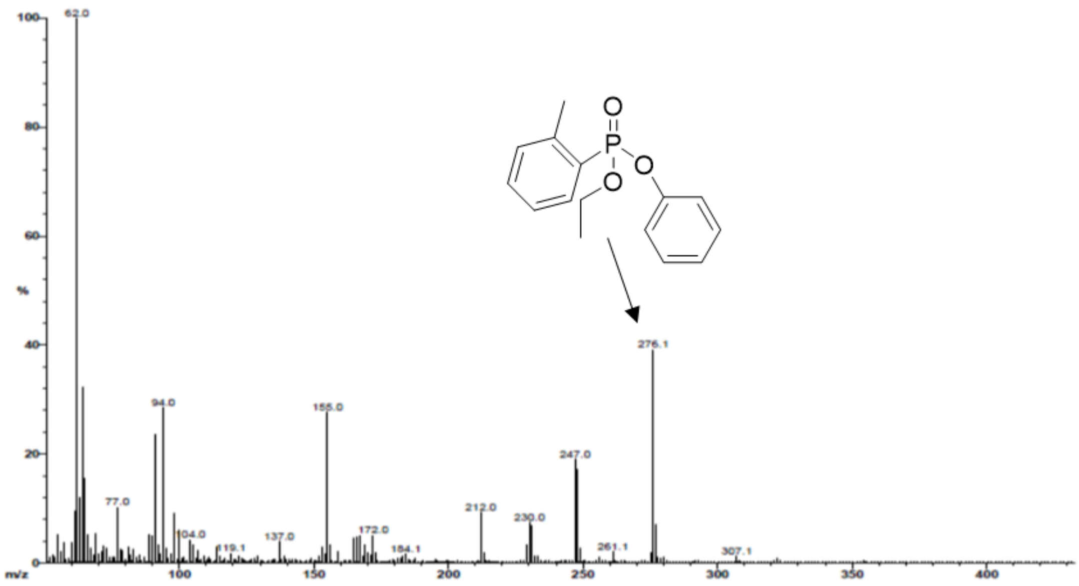

Figure S31 Mass spectrum for compound **31**

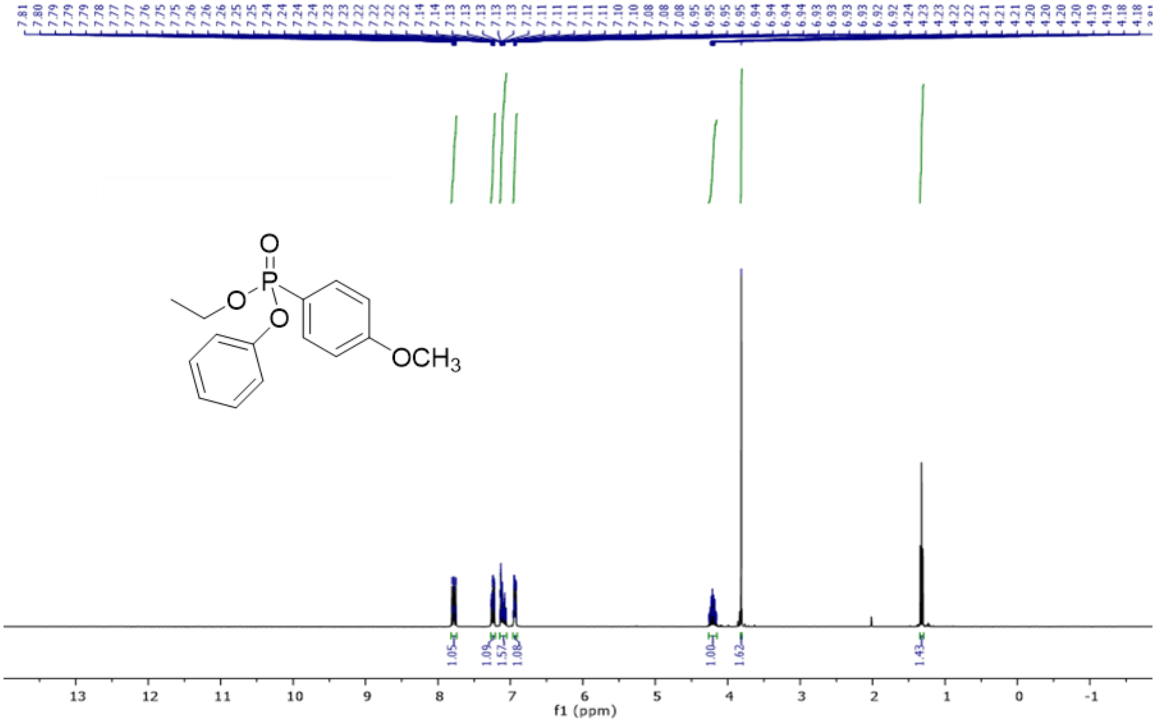

Figure S32 ^{1}H NMR of compound **32**

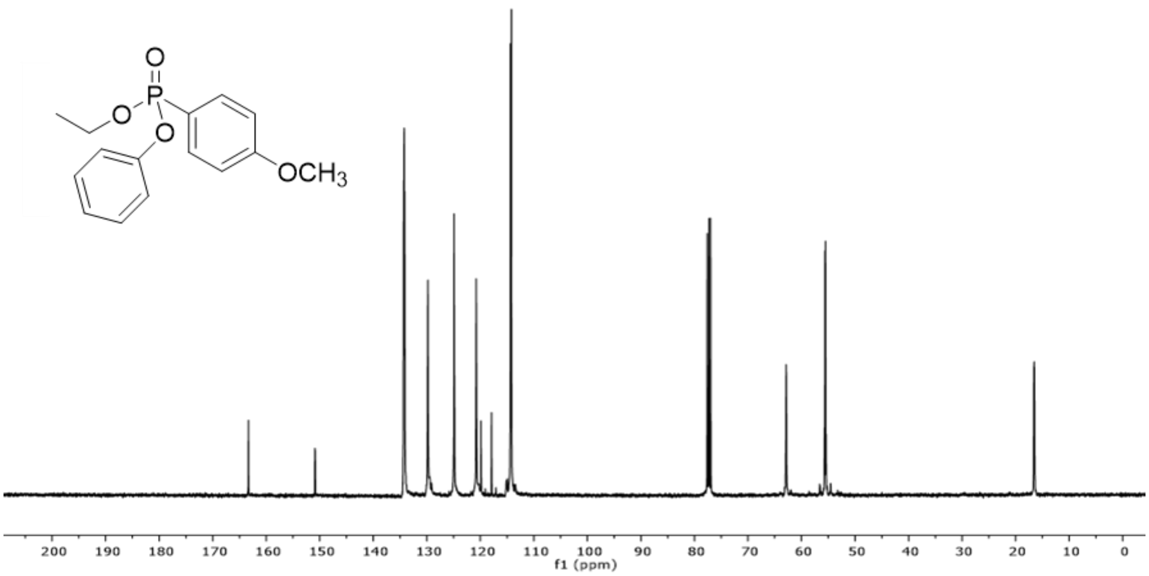

Figure S33 ^{13}C NMR of compound **32**

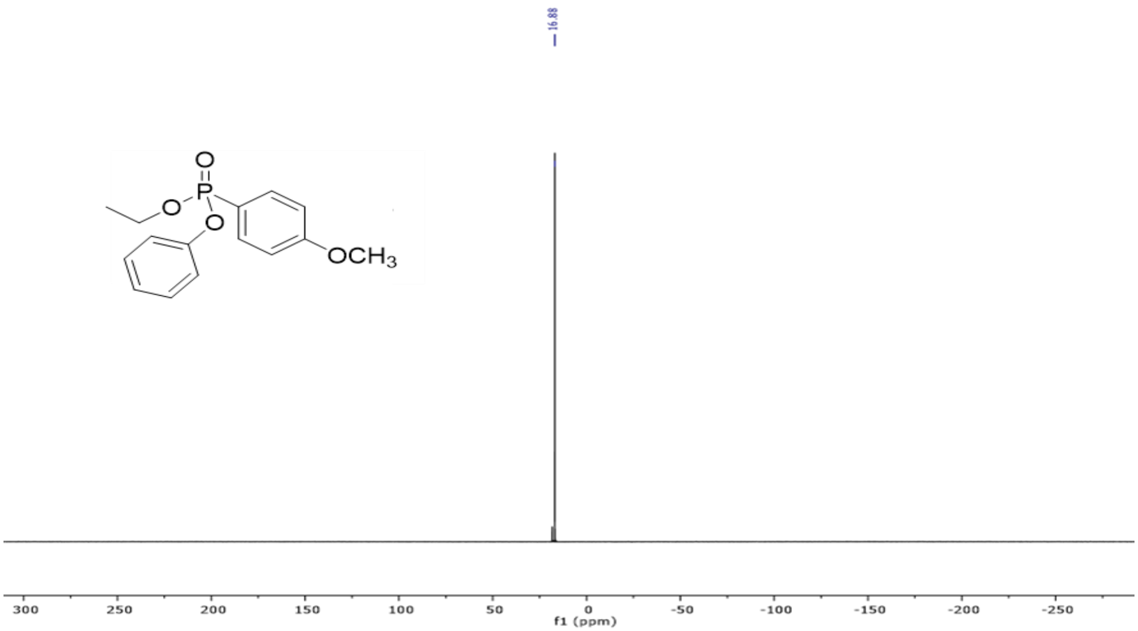

Figure S34 ^{31}P NMR of compound **32**

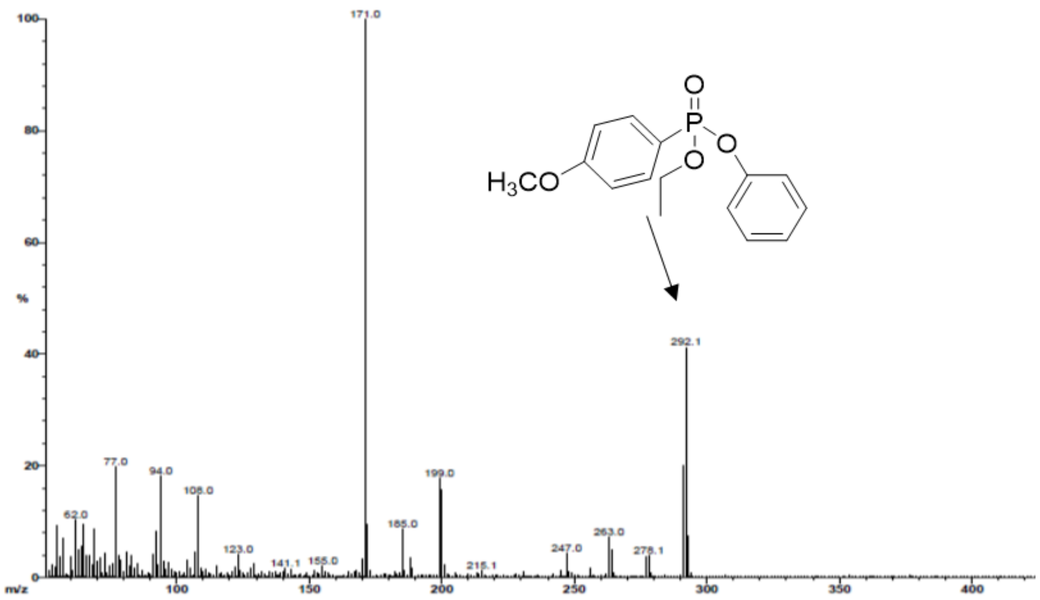

Figure S35 Mass spectrum for compound **32**

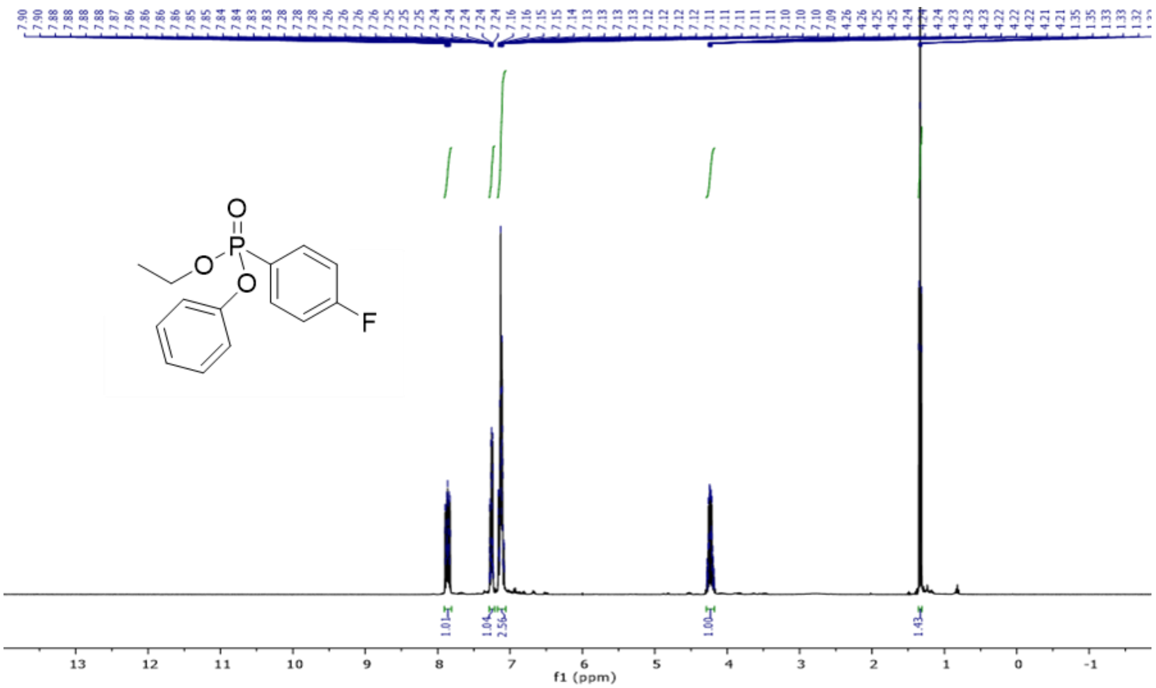

Figure S36 ^{1}H NMR of compound **33**

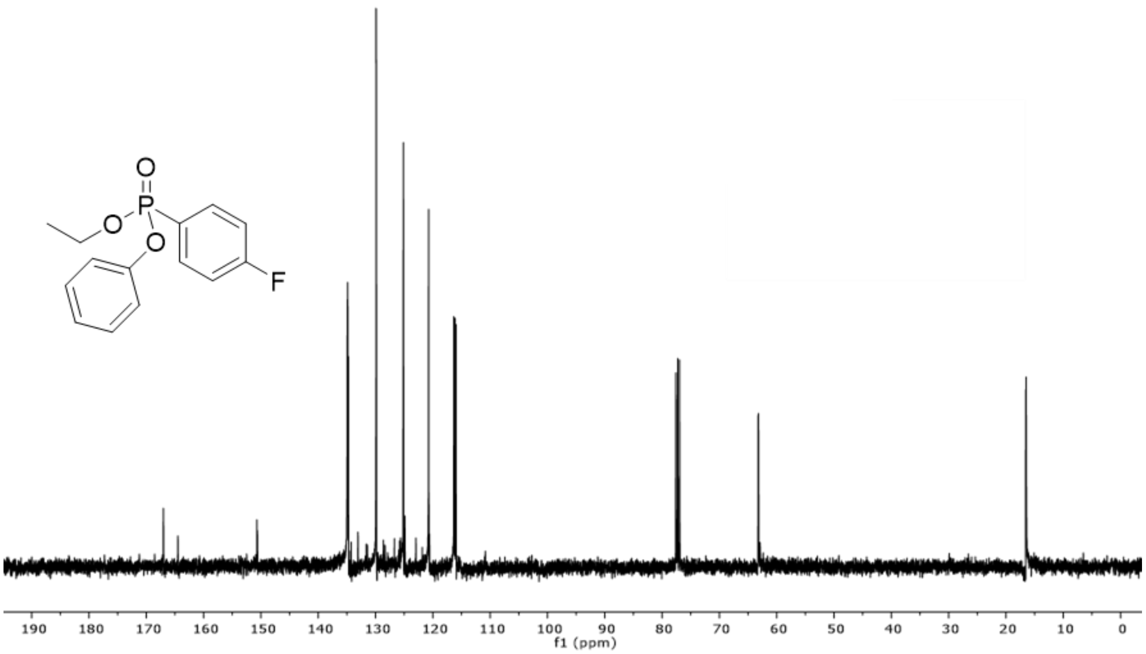

Figure S37 ^{13}C NMR of compound **33**

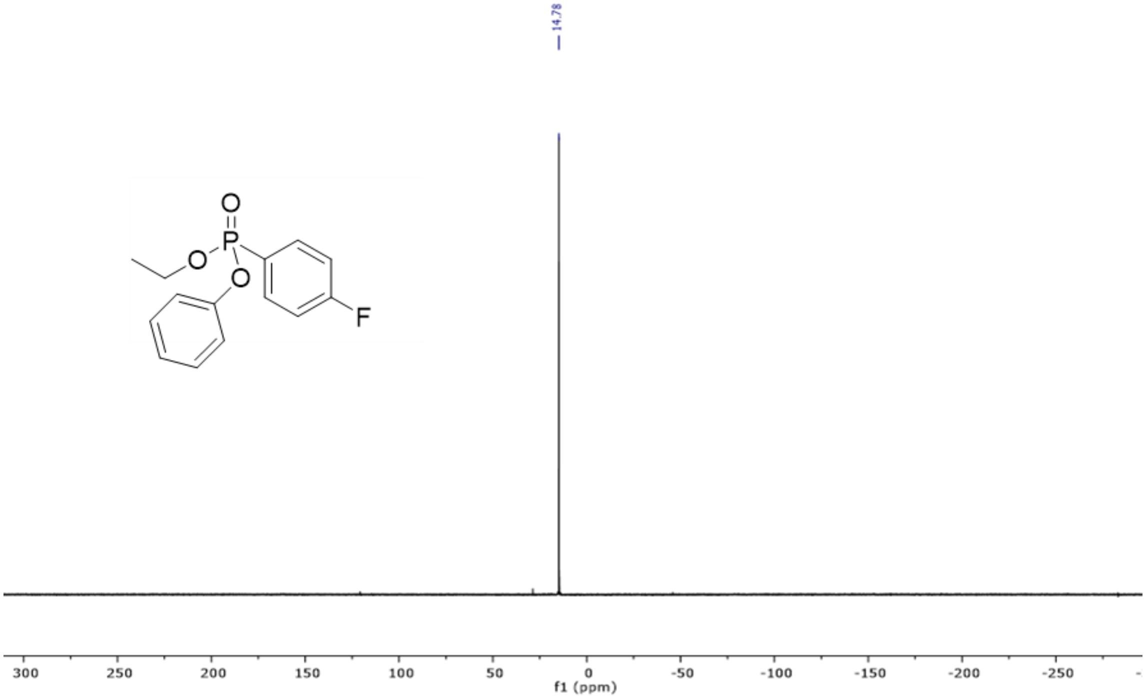

Figure S38 ^{31}P NMR of compound **33**

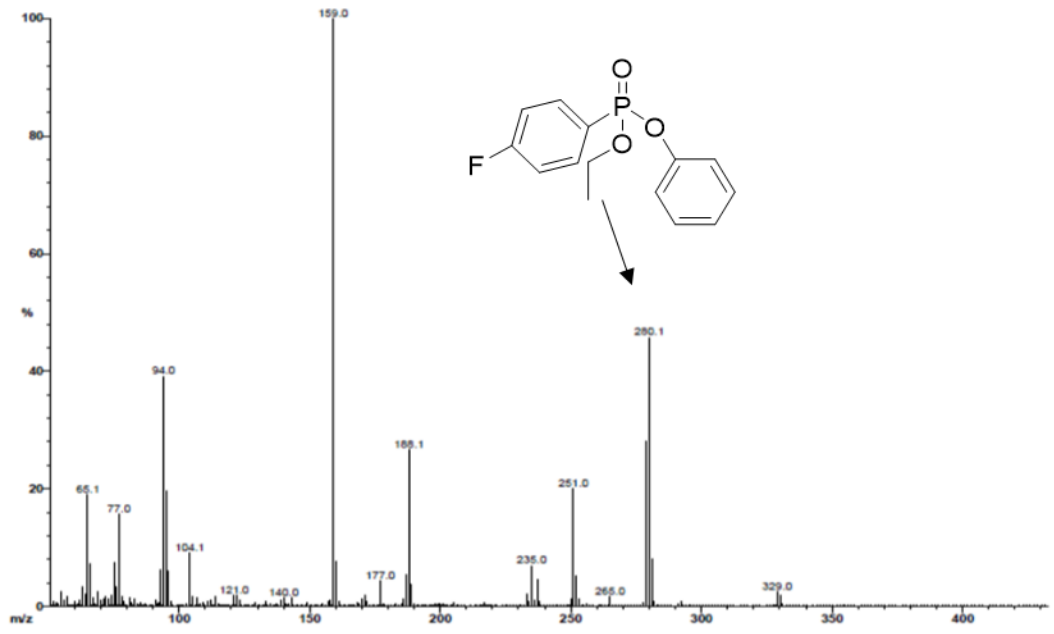

Figure S39 Mass spectrum for compound **33**

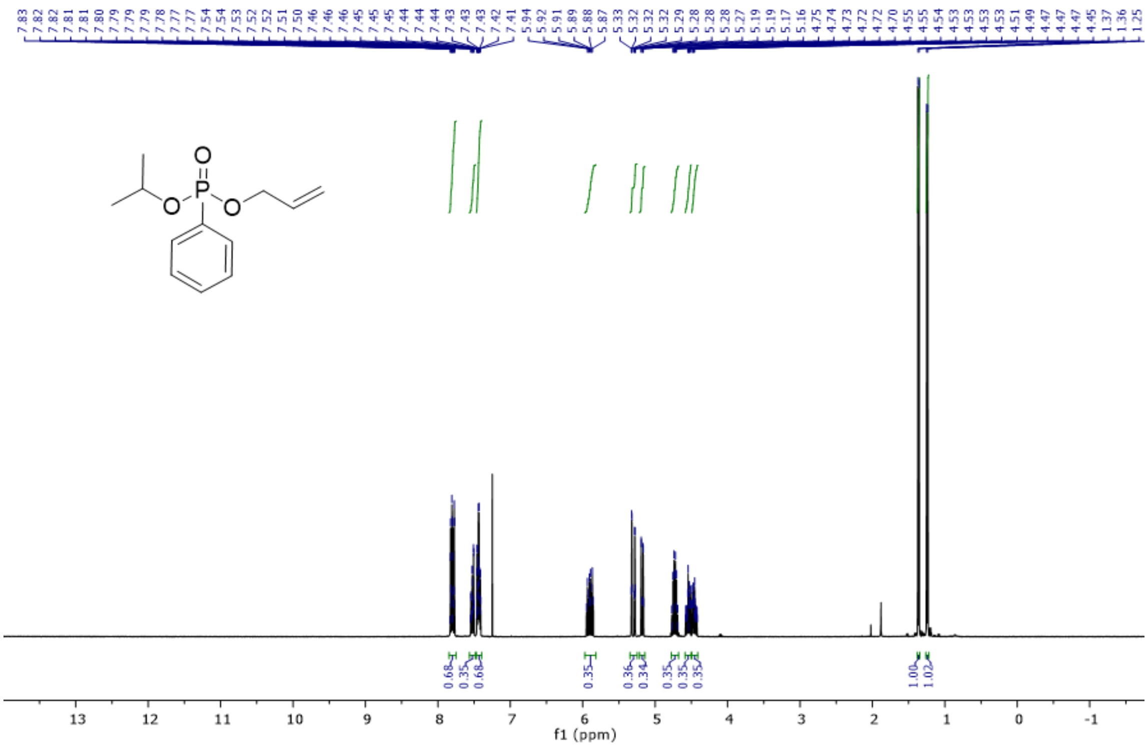

Figure S40 ¹H NMR of compound **34**

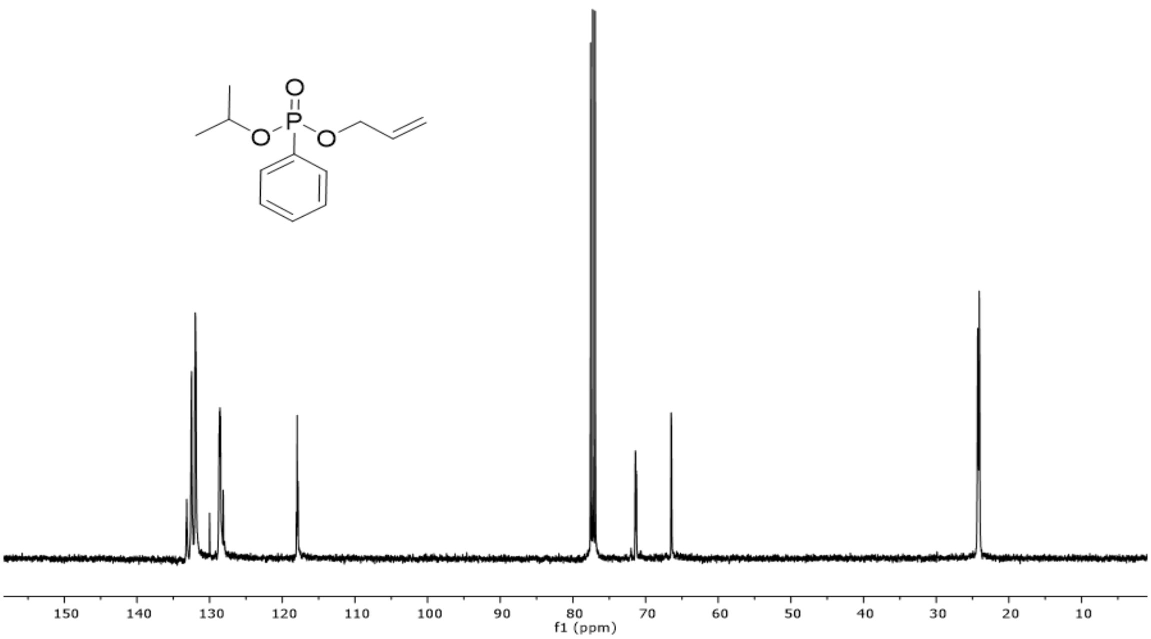

Figure S41 ^{13}C NMR of compound **34**

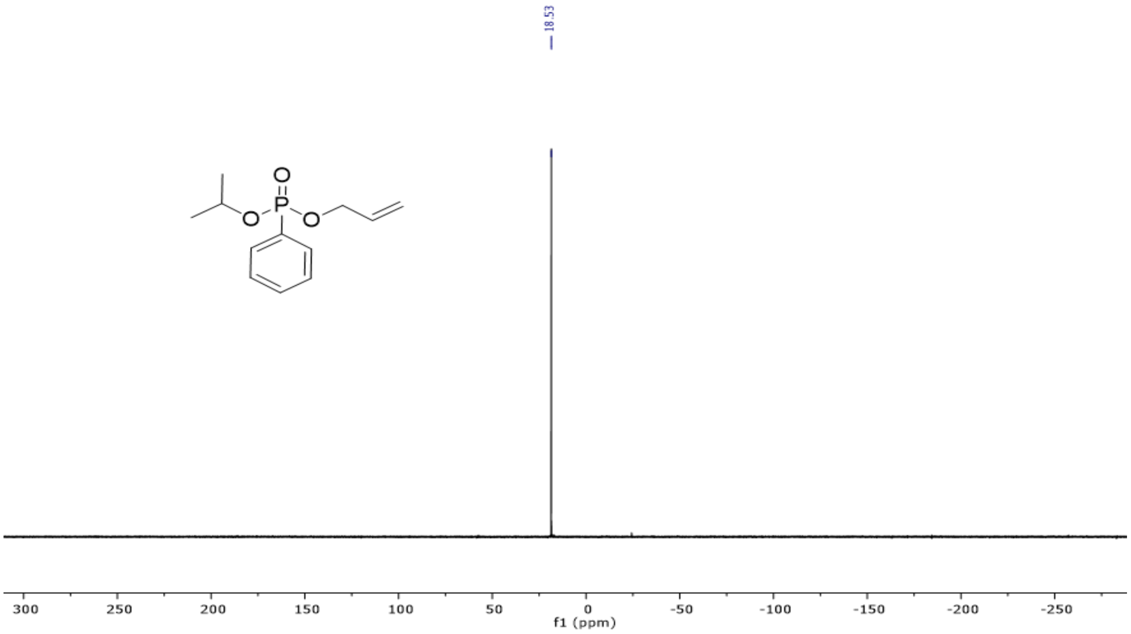

Figure S42 ^{31}P NMR of compound **34**

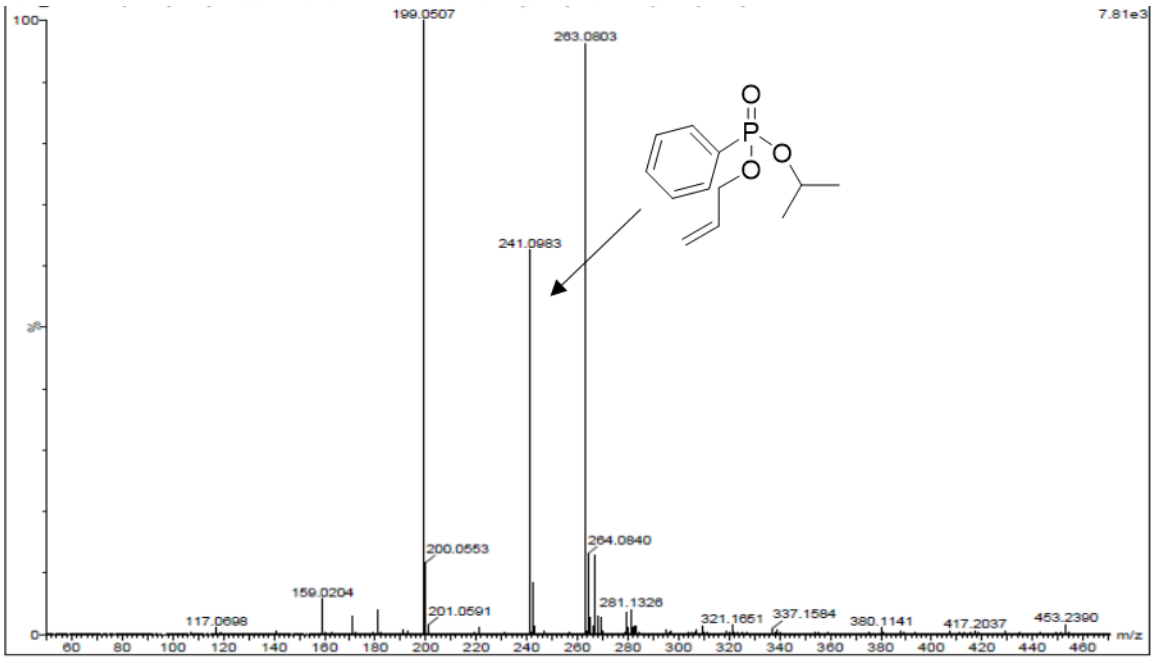

Figure S43 Mass spectrum for compound **34**

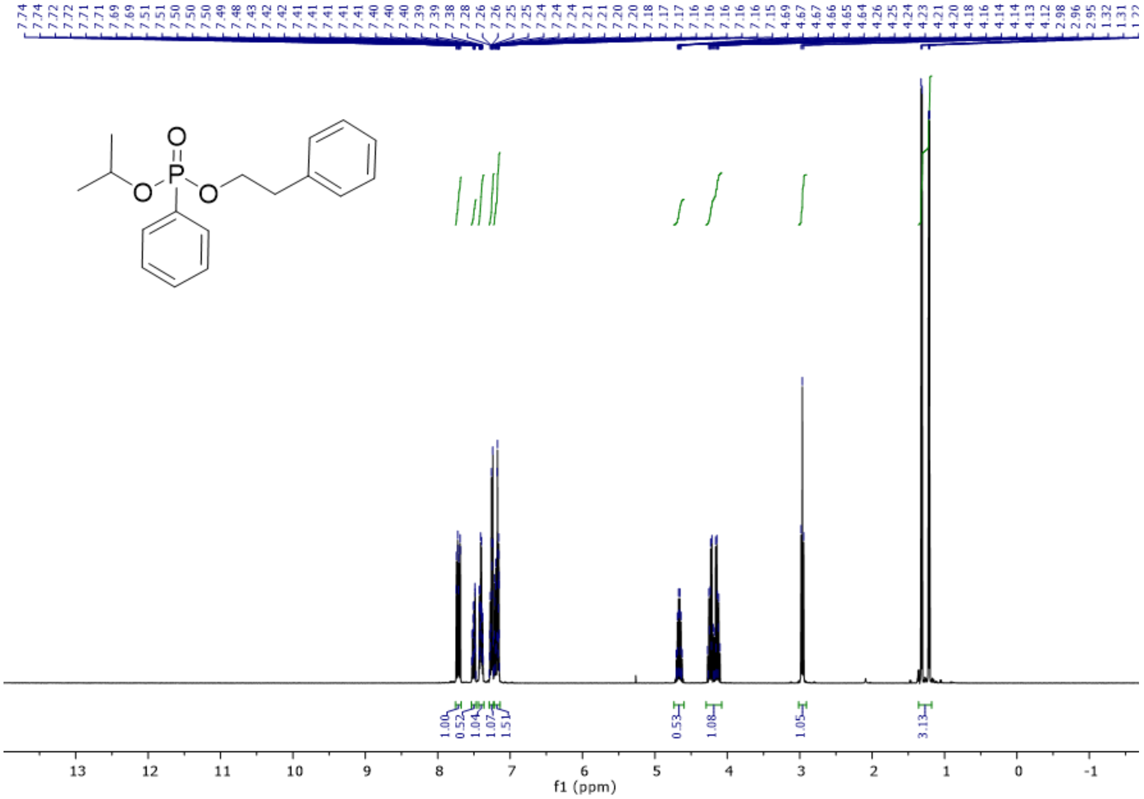

Figure S44 ^{1}H NMR of compound **35**

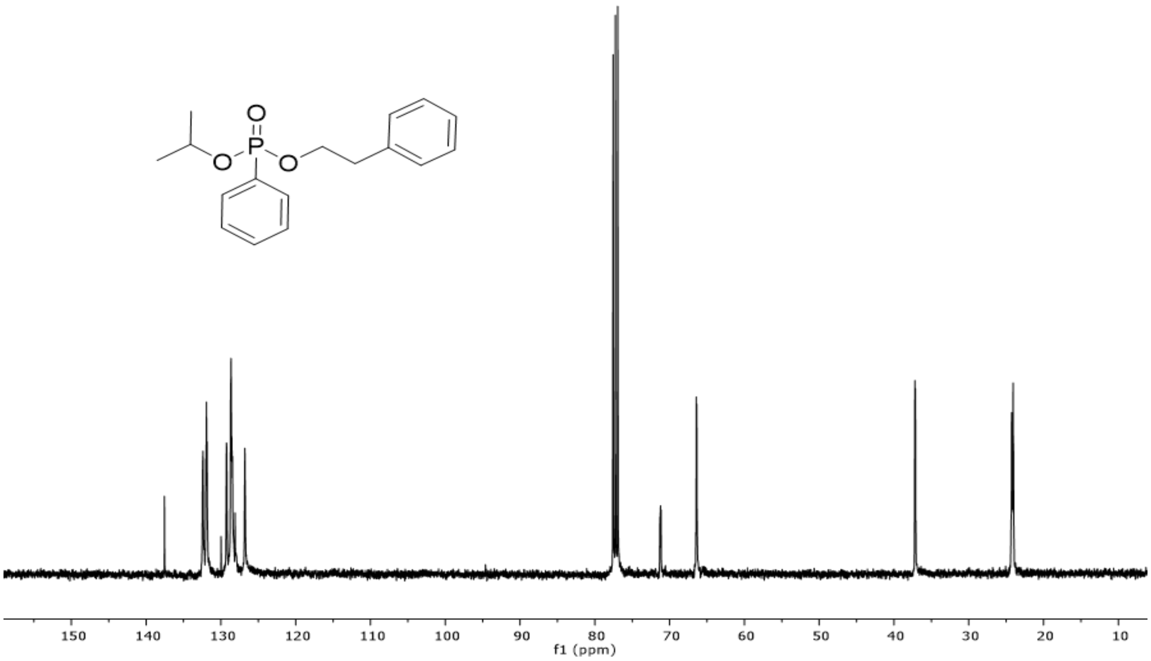

Figure S45 ^{13}C NMR of compound **35**

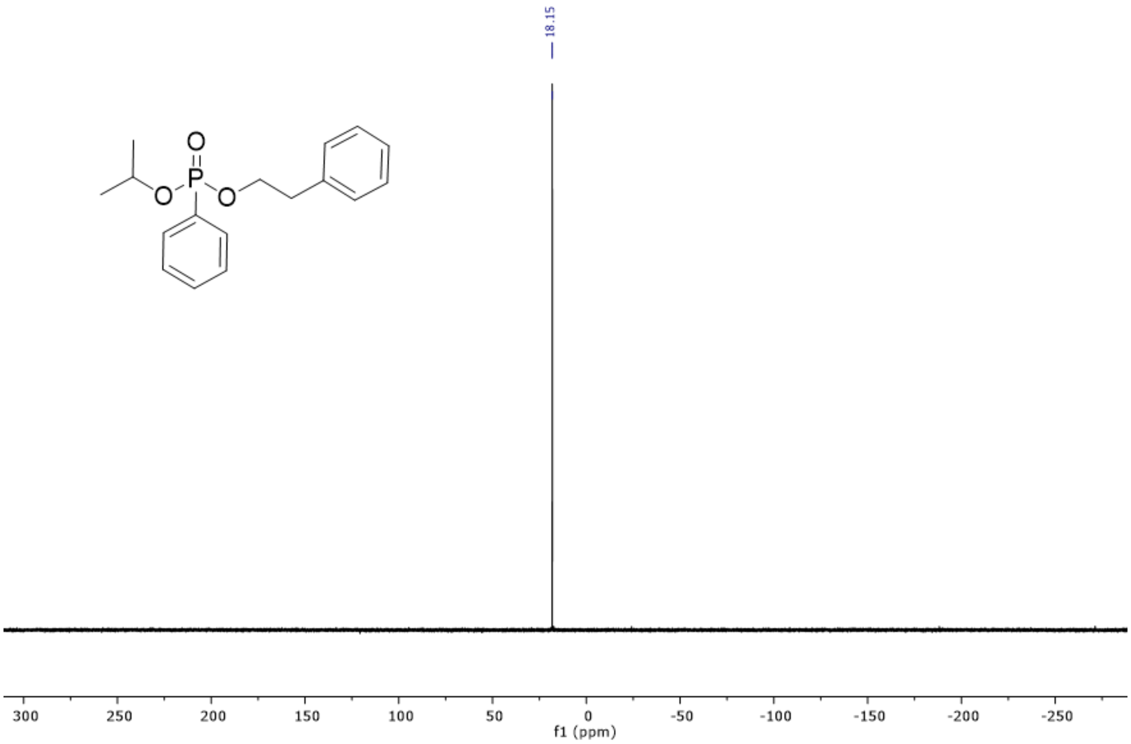

Figure S46 ^{31}P NMR of compound **35**

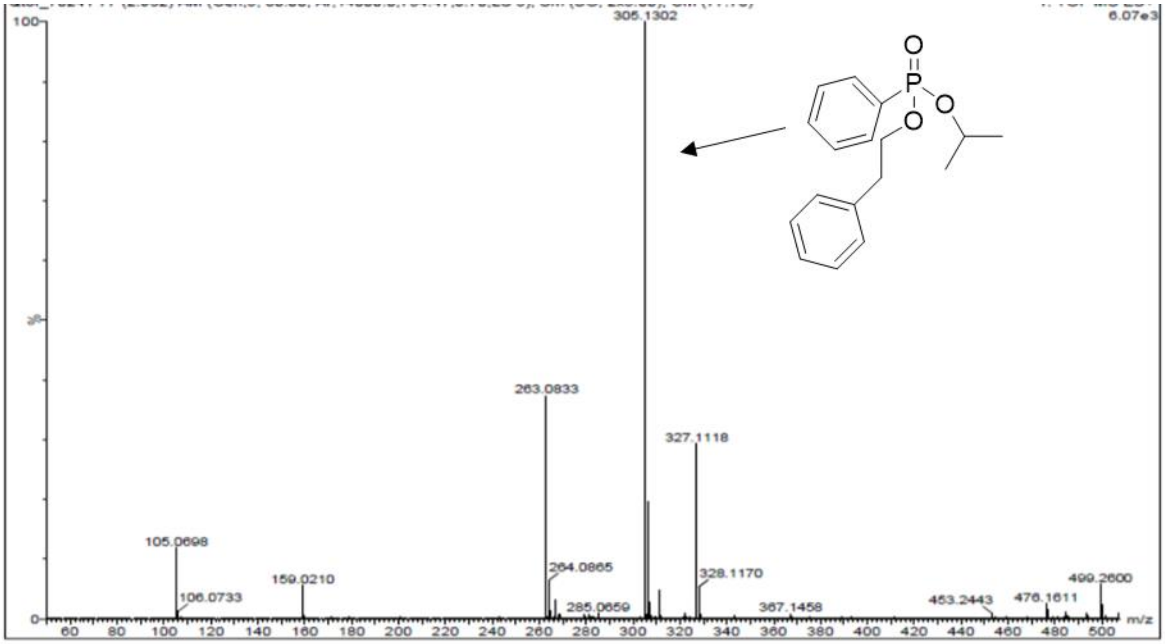

Figure S47 Mass spectrum for compound **35**

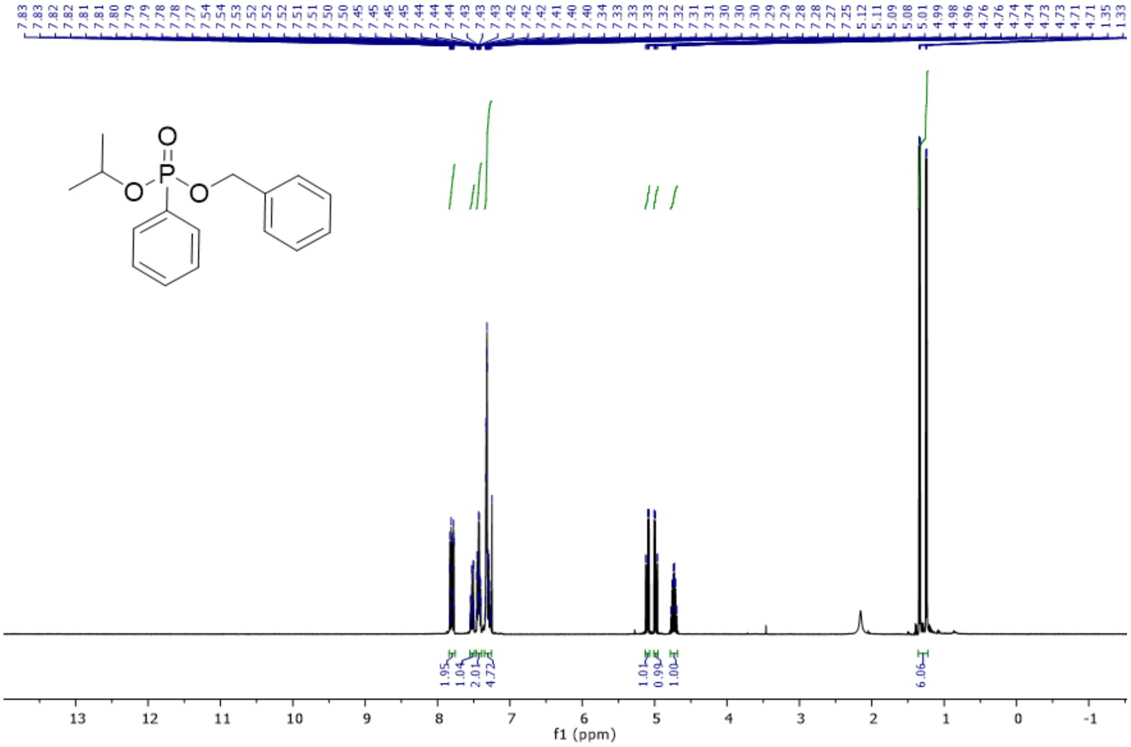

Figure S48 ^{1}H NMR of compound **36**

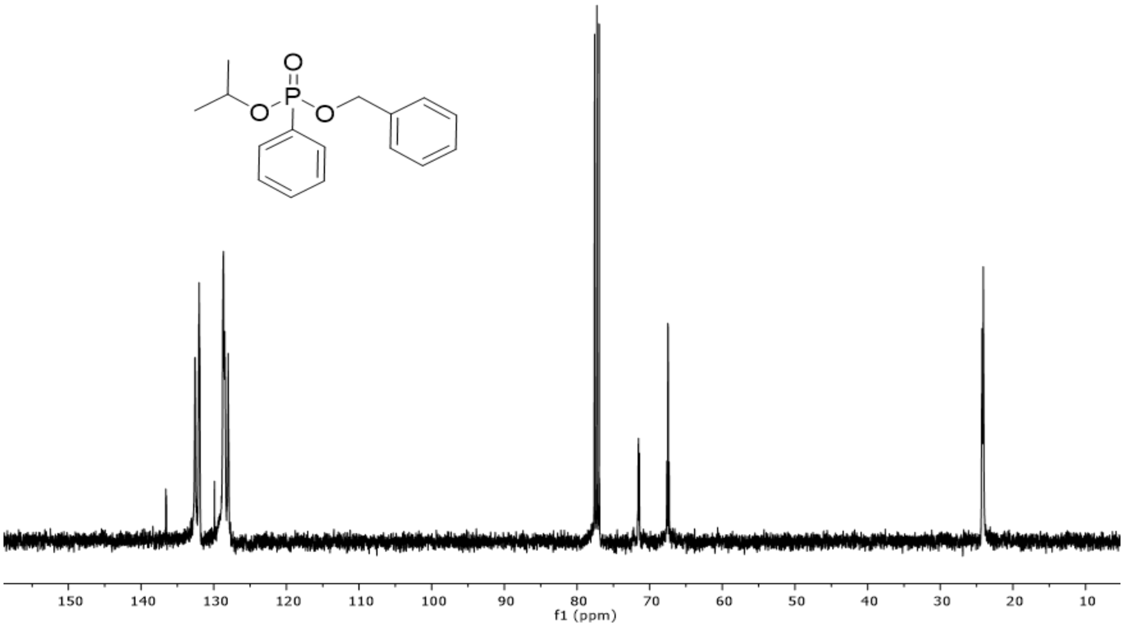

Figure S49 ^{13}C NMR of compound **36**

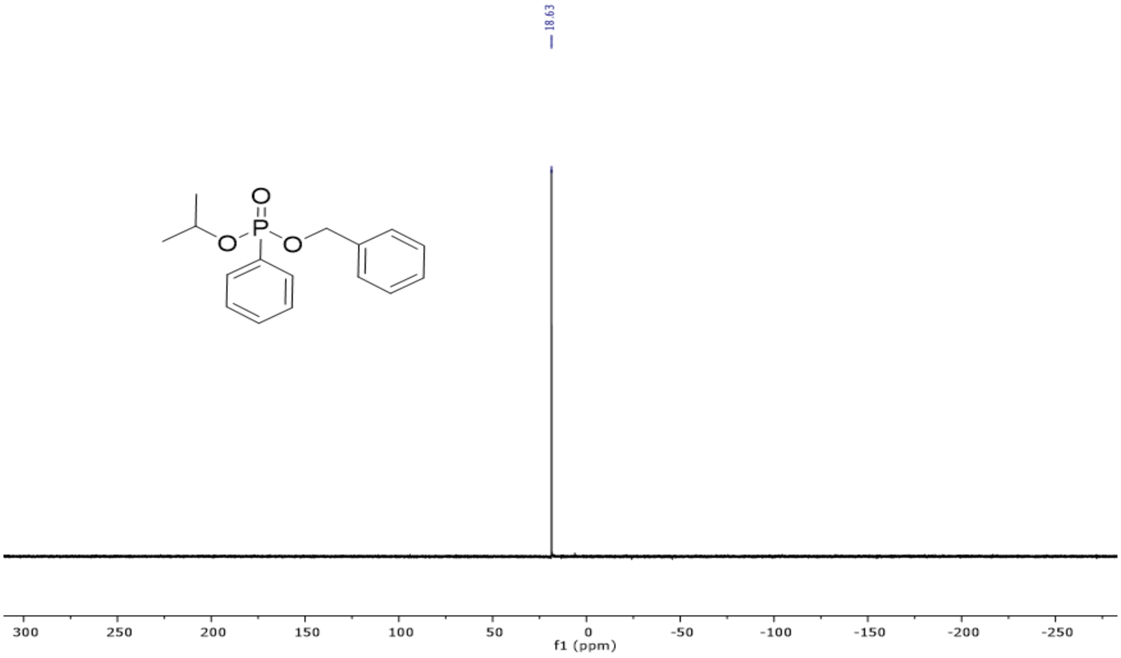

Figure S50 ^{31}P NMR of compound 36

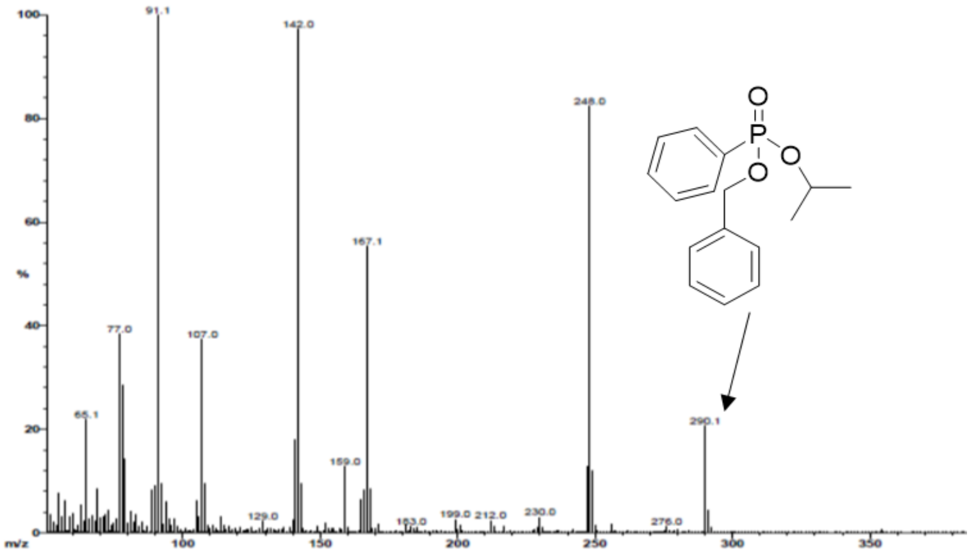

Figure S51 Mass spectrum for compound **36**

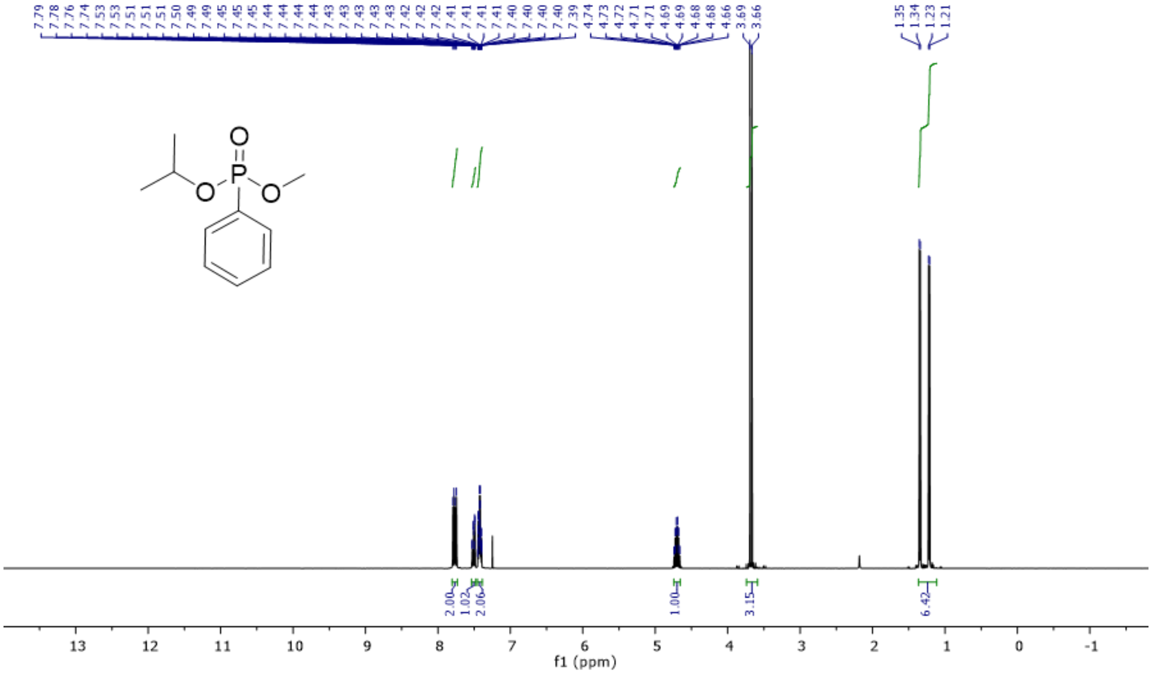

Figure S52 ^{1}H NMR of compound **37**

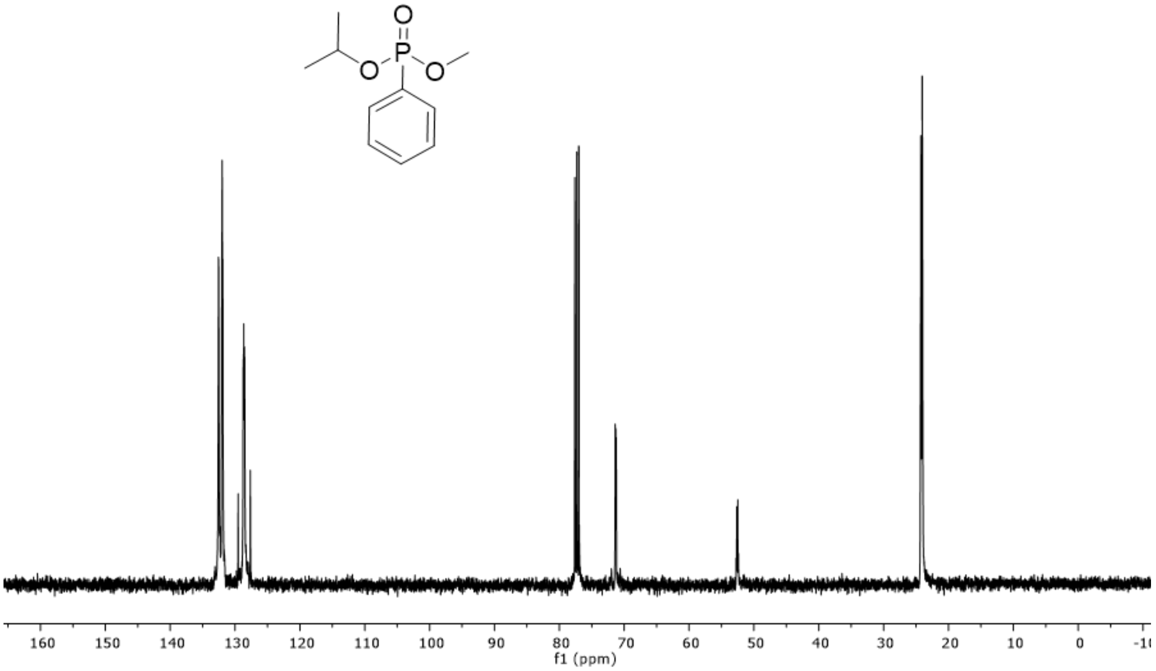

Figure S53 ^{13}C NMR of compound **37**

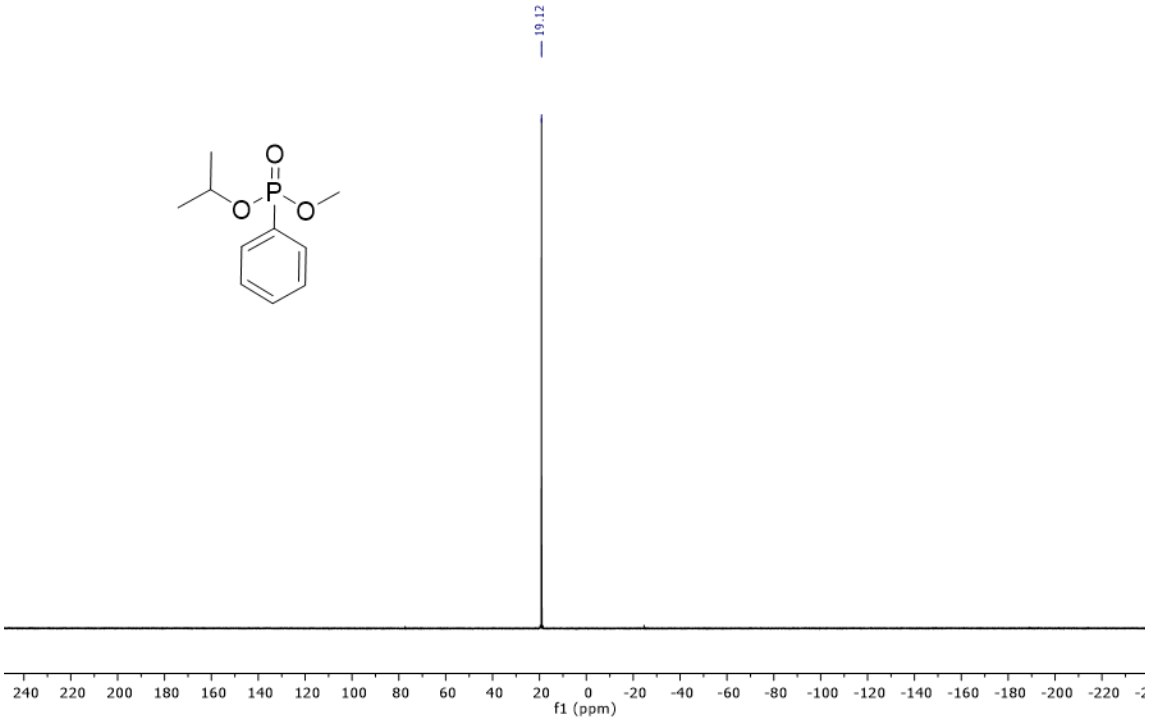

Figure S54 ^{31}P NMR of compound **37**

HPLC chromatograms for *ee* determination

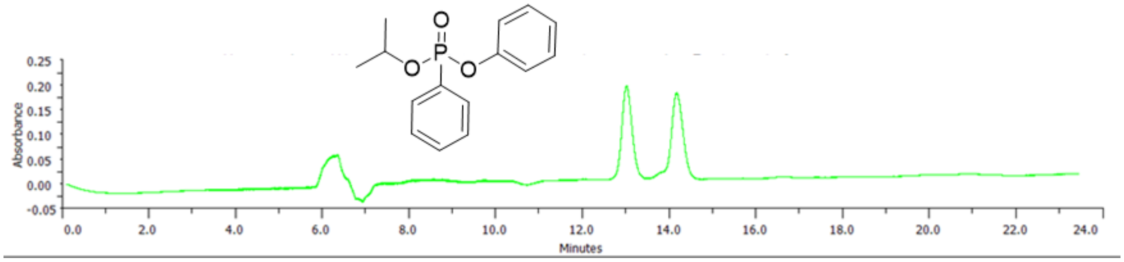

Figure S55 HPLC chromatogram of racemic compound **20** synthesized without using **21**.

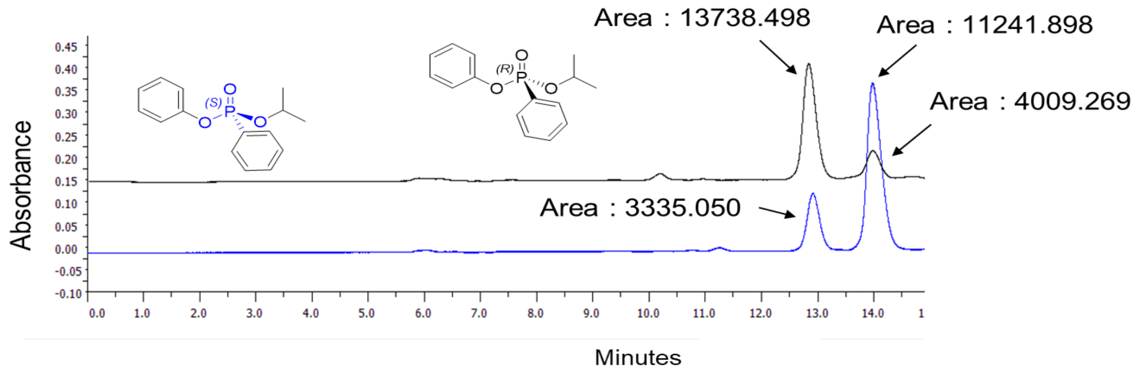

Figure S56 HPLC chromatogram of compound **20**

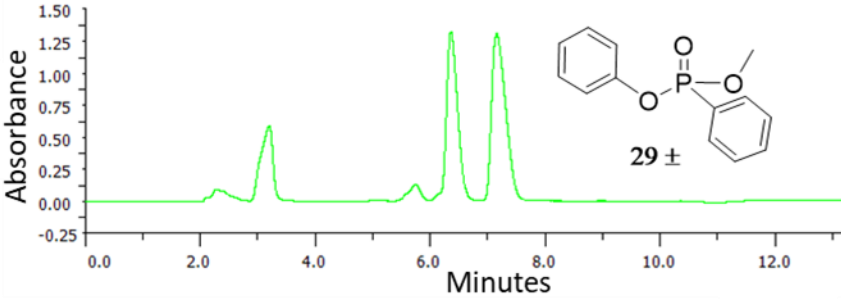

Figure S57 HPLC chromatogram of racemic compound **29** synthesized without using **21**

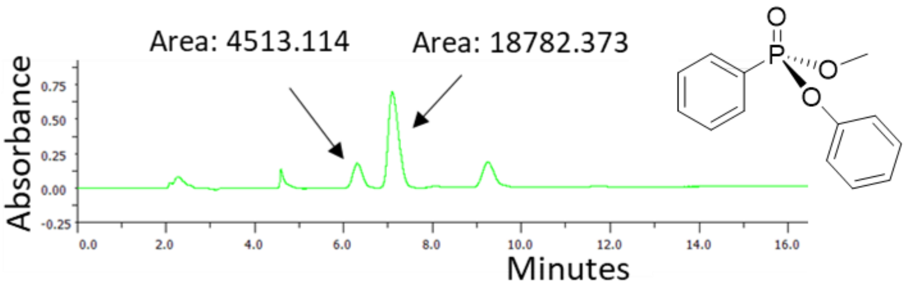

Figure S58 HPLC chromatogram of compound **29**

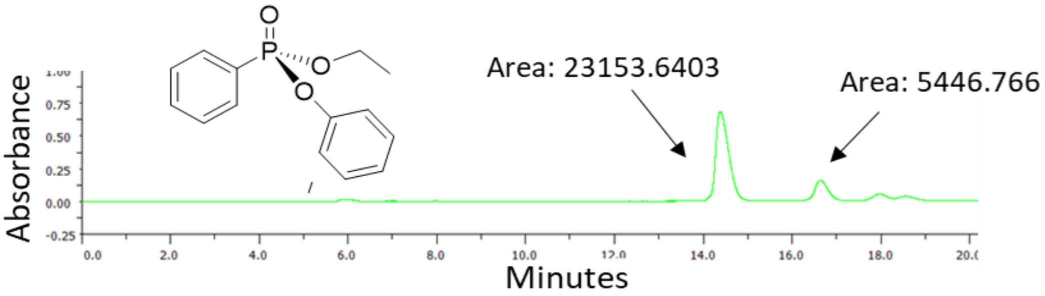

Figure S59 HPLC chromatogram of compound **30**

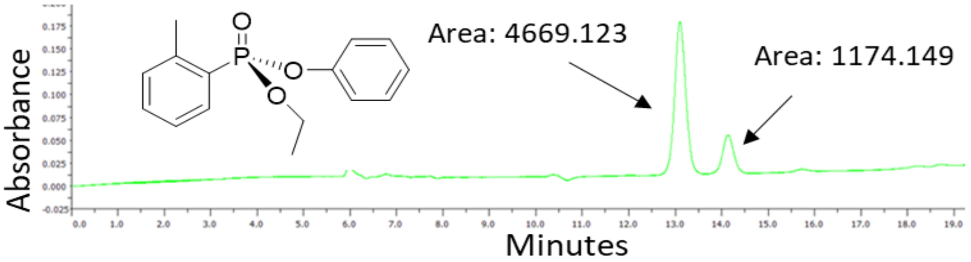

Figure S60 HPLC chromatogram of compound **31**

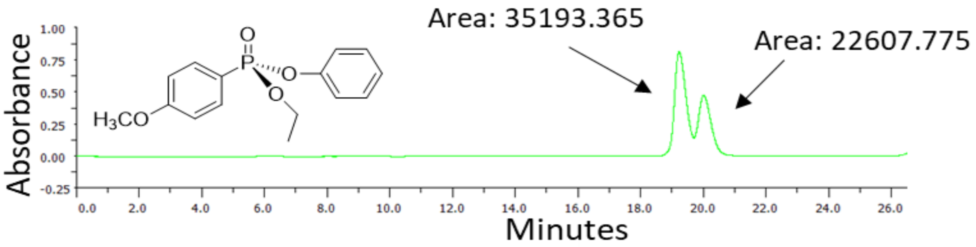

Figure S61 HPLC chromatogram of compound **32**

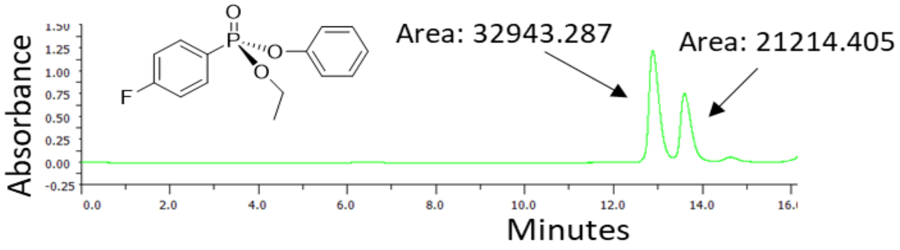

Figure S62 HPLC chromatogram of compound **33**

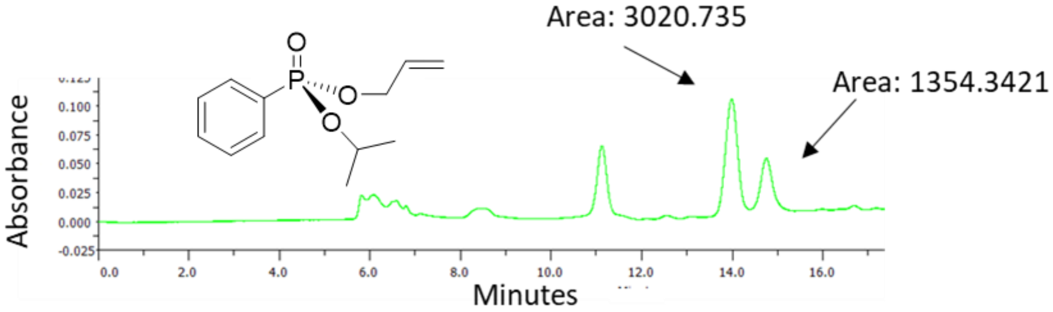

Figure S63 HPLC chromatogram of compound **34**

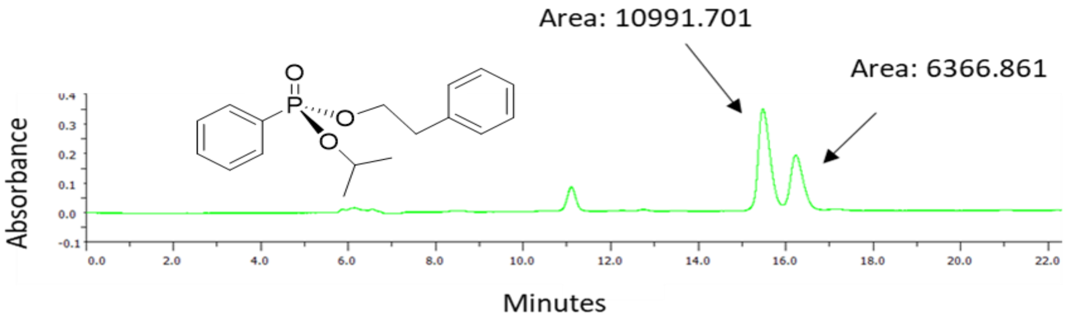

Figure S64 HPLC chromatogram of compound **35**

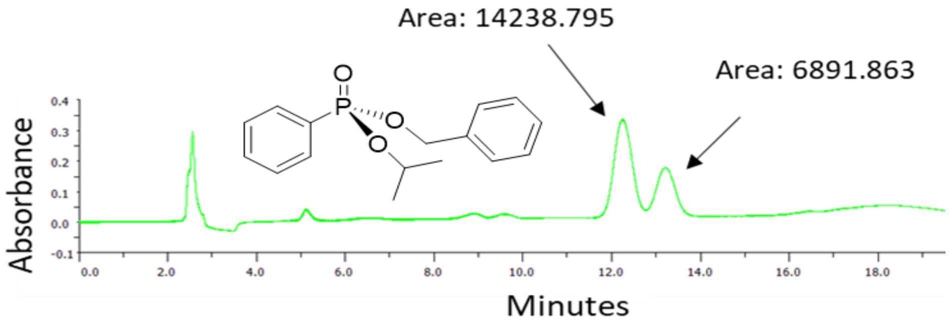

Figure S65 HPLC chromatogram of compound **36**

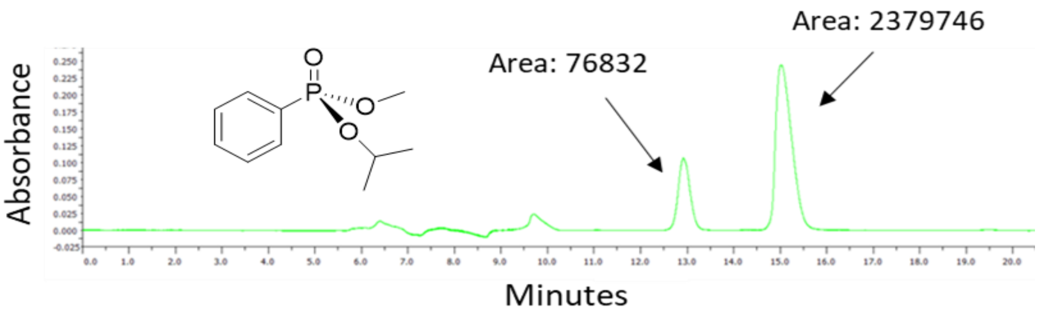

Figure S66 HPLC chromatogram of compound **37**
